# Rhodium-Catalyzed Tandem Asymmetric Allylic Decarboxylative Addition and Cyclization of Vinylethylene Carbonates with *N*-Nosylimines

**DOI:** 10.3390/molecules29051019

**Published:** 2024-02-26

**Authors:** Xiao-Lin Wang, Hai-Bin Jiang, Sheng-Cai Zheng, Xiao-Ming Zhao

**Affiliations:** School of Chemical Science and Engineering, Tongji University, 1239 Siping Road, Shanghai 200092, China; 1810923@tongji.edu.cn (X.-L.W.); haibin@tongji.edu.cn (H.-B.J.)

**Keywords:** rhodium, tridentate ligand, tandem reaction, allylic decarboxylation, cycloaddition, C−N bond formation

## Abstract

A enantioselective tandem transformation, concerning asymmetric allylic decarboxylative addition and cyclization of *N*-nosylimines with vinylethylene carbonates (VECs), in the presence of [Rh(C_2_H_4_)_2_Cl]_2_, chiral sulfoxide-*N*-olefin tridentate ligand has been developed. The reaction of VECs with various substituted *N*-nosylimines proceeded smoothly under mild conditions, providing highly functionalized oxazolidine frameworks in good to high yields with good to excellent enantioselectivity.

## 1. Introduction

**Scheme 1:**
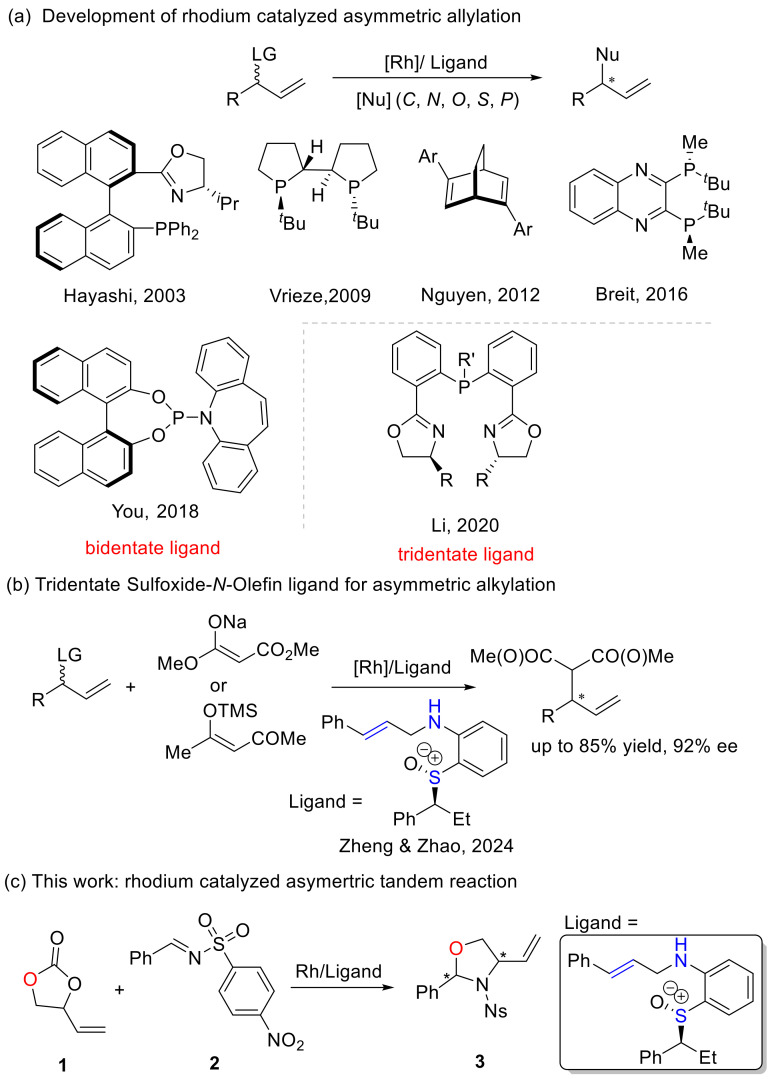
Background for rhodium catalyzed allylic substitution [6,7,9,13,14,15,22]. Note: The carbon with * represents a chiral center.

Rhodium-catalyzed asymmetric allylic substitution, among the efficient transition-metal catalysts, has gained increasing attention due to the development of chiral ligands in recent years [1,2,3,4,5]. Since Hayashi [6] reported the first Rh-catalyzed asymmetric allylic alkylation employing a bidentate *N*,*P*-ligand in 2003, a variety of bidentate ligands has been discovered or designed to succeed in the asymmetric allylic substitution reaction [7,8,9,10,11,12,13,14]. Recently, Li [15,16,17,18,19] developed a Rh/*N*,*P*,*N*-tridentate ligand [20] catalysis system, which has proven to be a powerful tool for catalyzing asymmetric allylic alkylation (AAA) reaction. A broad spectrum of nucleophiles including C, N, O, S, and even P [21] are compatible, showing a vigorousness of the tridentate ligand for allylic reaction (Scheme 1a). Very recently, we designed a tridentate sulfoxide-*N*-olefin hybrid Ligands and applied them in Rh-catalyzed asymmetric allylic alkylation [22] (Scheme 1b). In this study, the coordination of the olefin to rhodium is crucial to the enantioselectivity but becomes hemilabile once the catalytic cycle is finished.

Tandem reactions involving transition metal catalyzed decarboxylative addition and cyclozation of vinylethylene carbonates (VECs) are efficient ways to construct heterocycles. The key to these conversions is reckoned the in situ formation of zwitterionic *π*-allyl palladium intermediates during CO_2_ extrusion [23]. These intermediates consistently act as efficient C, O-dipoles, facilitating [3 + n] [24,25,26,27,28,29,30,31] and [5 + n] [32,33,34,35,36,37,38,39] annulation reactions for the construction of structurally diverse five- to medium-membered rings. However, Rh has rarely been reported in such enantioselective transformations despite the blooming allylic substitution reaction developed. As a continuous interest in the tridentate ligand applied in Rh-catalysis, herein we report a rhodium-catalyzed tandem asymmetric allylic decarboxylative addition and allylic substitution of vinylethylene carbonates with *N*-nosylimines (Scheme 1c).

## 2. Results and Discussion

We performed our initial study using 4-Vinyl-1,3-dioxolan-2-one **1a** with *N*-nosylimines **2a** for this reaction. In the presence of 1 mmol% of [Rh(C_2_H_4_)_2_Cl]_2_, 2 mmol% of Sulfoxide ligand **L1** and Cs_2_CO_3_ in dichloromethane (DCM) at 25 °C, the expected cycloadduct **3** as diastereomers could be obtained in 32% yield with 41%/37% ee (Table 1, entry 1). And the diastereomers **3a** and **3a′** can be separated through a column chromatograph.

Solvents, such as toluene, THF, MeCN and DCE (1,2-dichloroethane), were next screened (entries 2–5). This investigation led to the finding that DCE was optimal in terms of reactivity and enantioselectivity, giving the desired product **3** as in a 35% total yield, 53%/73% ee and 1.2/1 dr (entry 5 vs. entries 1–5). The base played an important role in the cycloaddition, and a series of bases including CsF, K_2_CO_3_, CsCl, Et_3_N, 1,8-diazabicyclo [5.4.0]-7-undecene (DBU) and *N,N,N,N*′-tetramethyl ethylenediamine (TMEDA) were tested (entries 5–11), CsF gave **3a/3a**′ in a poor yield but with good enantioselectivity and diastereoselectivity (entry 6). Et_3_N was identified as the optimal base that could dramatically improve the yield and enantioselectivity (entry 9). The reaction was performed at a temperature ranging from −25 °C to 25 °C (entries 9, 12–14), and we found that the reaction at −10 °C gave superior results, giving rise to desired products in 61% yield with with good ee values (entry 13). Rhodium salts, such as Rh(acac)(C_2_H_4_)_2_, and [Rh(cod)Cl]_2_, were surveyed. Rh(acac)(C_2_H_4_)_2_ gave **3a/3a**′ in a poor yield and low ee value (entry 15). While [Rh(cod)Cl]_2_ was not suitable for this reaction (entry 16).

**Figure 1:**
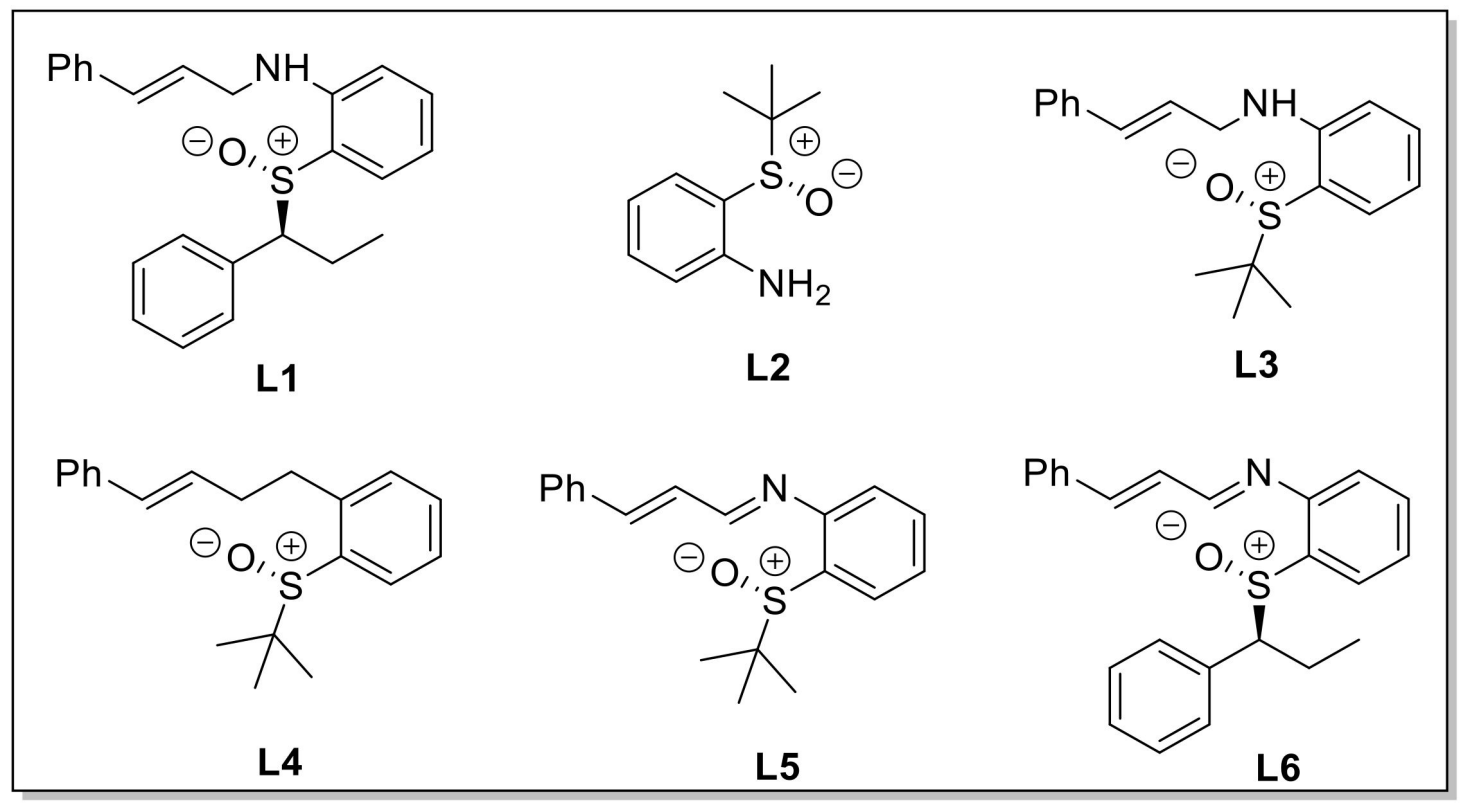
Ligands used in this reaction.

When the catalyst loading was increased to 2 mol%, the yield and enantioselectivity of the reaction was increased (entry 17). Ligand is crucial to asymmetric allylic substitution. Therefore, structurally varying Sulfoxide ligands such as **L1**, **L2**, **L3**, **L4**, **L5,** and **L6** were explored (Figure 1). The bidentate sulfoxide ligands **L2** and **L4** were less reactive, and the compounds **3a/3a′** were obtained with low yield and enantioselectivity (entry 18 and entry 20). Chirality only at sulfoxide (**L3**) led to decreased yield and enantioselectivity (entry 19). When the sulfoxide ligands containing imine fragments was used, the enantioselectivity dropped despite of the good yield (entry 21 and 22).

**Scheme 2:**
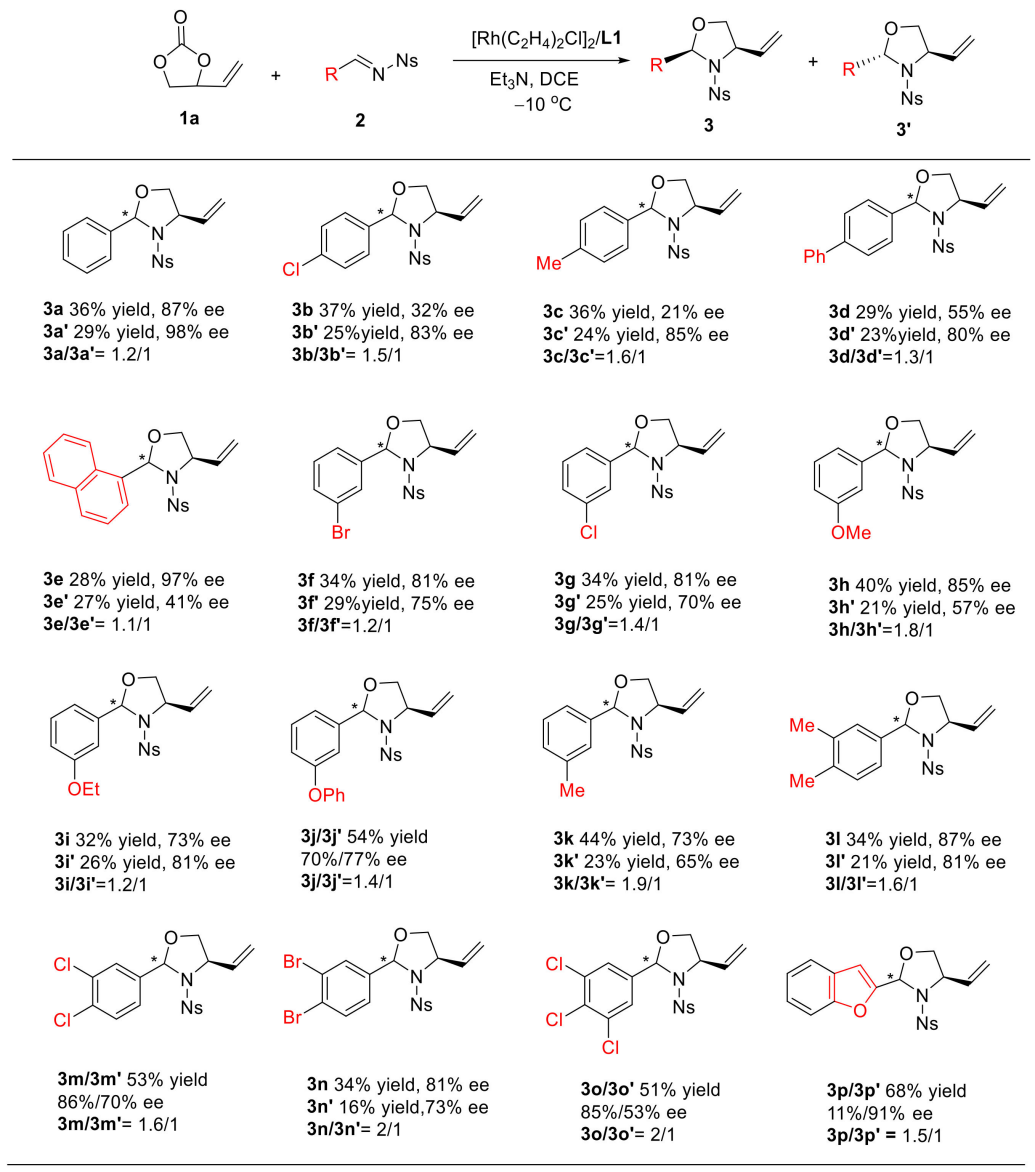
Scope of the allylic substrates **2** ^ab^. ^a^: Reaction conditions: [Rh(C_2_H_4_)_2_Cl]_2_ (0.004 mmol), **L1** (0.008 mmol), **1a** (0.40 mmol), **2** (0.20 mmol), Et_3_N (0.30 mmol), and DCE (2.0 mL). ^b^: Yield referred to isolated yield and ee was determined by a chiral HPLC. Note: The carbon with * represents a chiral center.

Having optimized reaction conditions in hand, the generality of the Rh-catalyzed asymmetric cycloaddition was examined using various *N*-nosylimines **2** (Scheme 2). The substrates **2b**, **2f**, **2g** containing the electron-withdrawing substituent (e.g., *p*-Cl, *m*-Br and *m*-Cl) on the phenyl ring resulted in allyl products **3b′**, **3f/3f′** and **3g/3g′** in moderate to good yields with slight decreased ee values. Products **3c′**, **3d′** were all isolated in good enantioselectivity. The para phenyl substituent led to a decreased yield (**3d/3d′**). 1-naphthyl substituted imine was also suited for the protocol, and **3e/3e′** were garnered in a 55% total yield with 97% ee and 41% ee, respectively. Similarly, *N*-nosylphenylimines bearing ether substituents (–OMe, –OEt, –OPh) on the phenyl ring were well tolerated under these catalytic conditions, leading to **3h**–**3j** in good yields (up to 61%) and enantioselectivities (up to 85% ee). In contrast, the *N*-nosylimines bearing a *m*–Me substituent showed low enantioselectivities but provided good yield in 67%. Interestingly, attaching multi-substituted groups on the phenyl ring, **3m/3m′**, **3n/3n′** and **3o** were also achieved in good to high yield with a high level of enantioselectivities. Heteroaryl- substrates **2p** gave **3p/3p′** in an excellent yield and enantioselectivity (Scheme 2).

**Figure 2:**
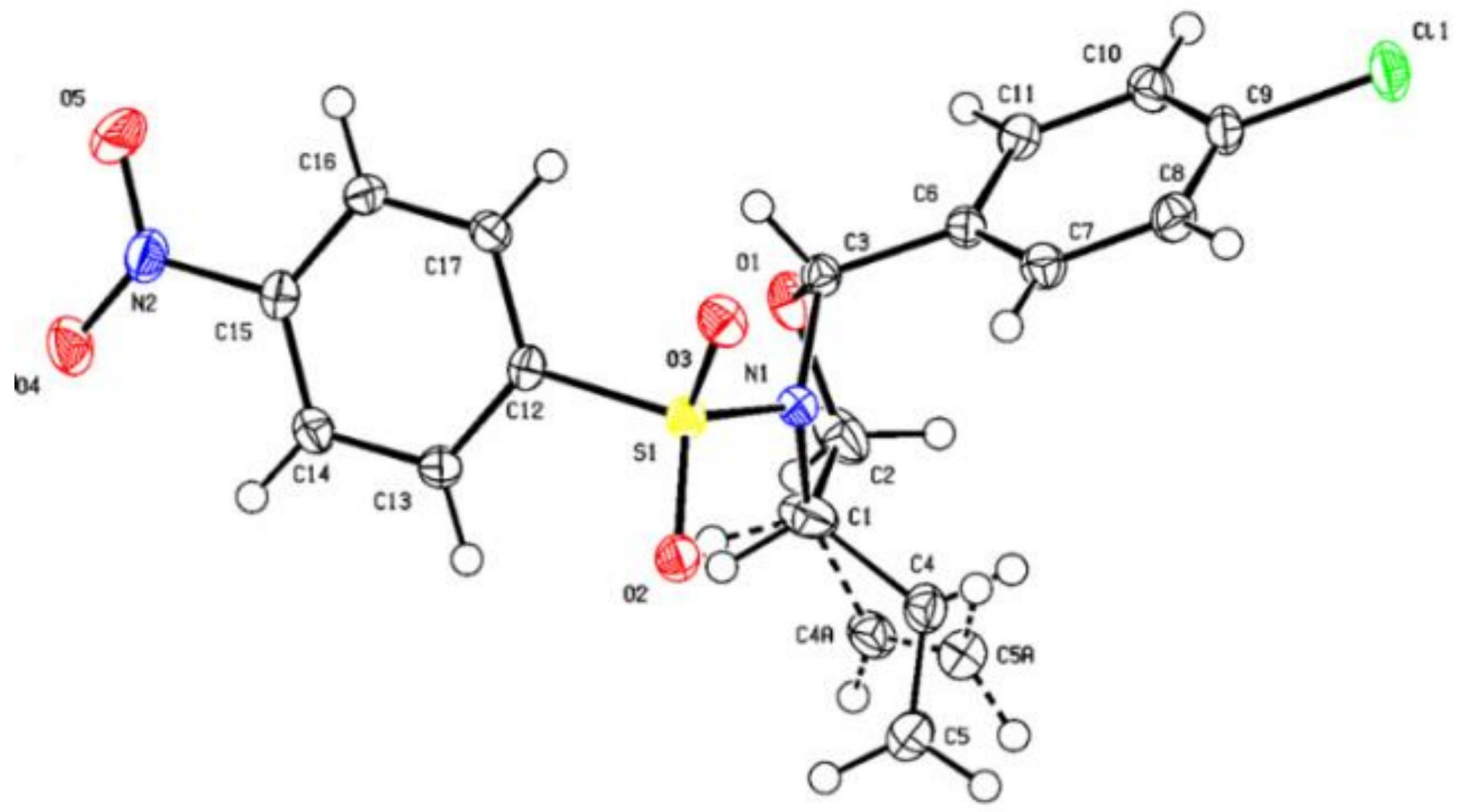
X-ray structure of **3b**.

An X-ray diffraction analysis of the compound **3b** enabled the determination of the absolute configuration of the two newly formed stereocenters [40] (Figure 2).

**Scheme 3:**
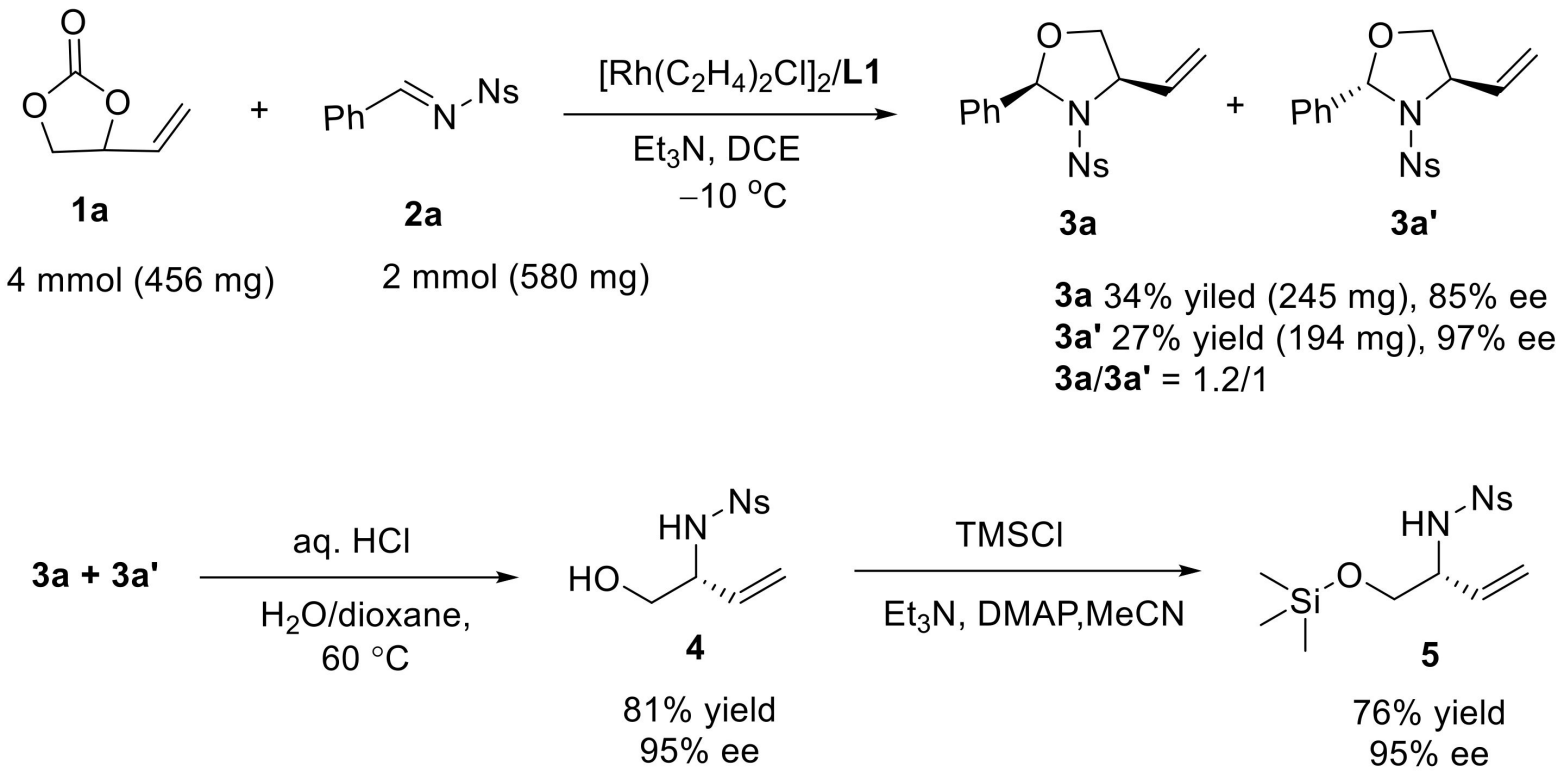
Scale-up synthesis of **3a/3a′** and the transformation for oxazolidines.

The scale-up synthesis of chiral oxazolidine products **3a/3a′** was conducted under the optimal conditions as shown in Scheme 3. **1a** (456 mg, 4 mmol) and **2a** (580 mg, 2 mmol) were employed and **3a/3a′** (440 mg, 61% yield, 85%/97% ee and 1.2/1 dr) was obtained (Scheme 3). To further demonstrate the synthetic utility of this approach, oxazolidine **3a**/**3a′** was successfully converted into the corresponding β-*tert*-β-amino alcohol under established conditions [41]. Direct hydrolysis of **3a**/**3a′** under acidic conditions yielded amino alcohol **4** with 81% yield and 95% ee. Subsequent protection of the alcohol with chlorotrimethylsilane in acetonitrile gave **5** (76% yield and 95% ee) [42].

**Scheme 4:**
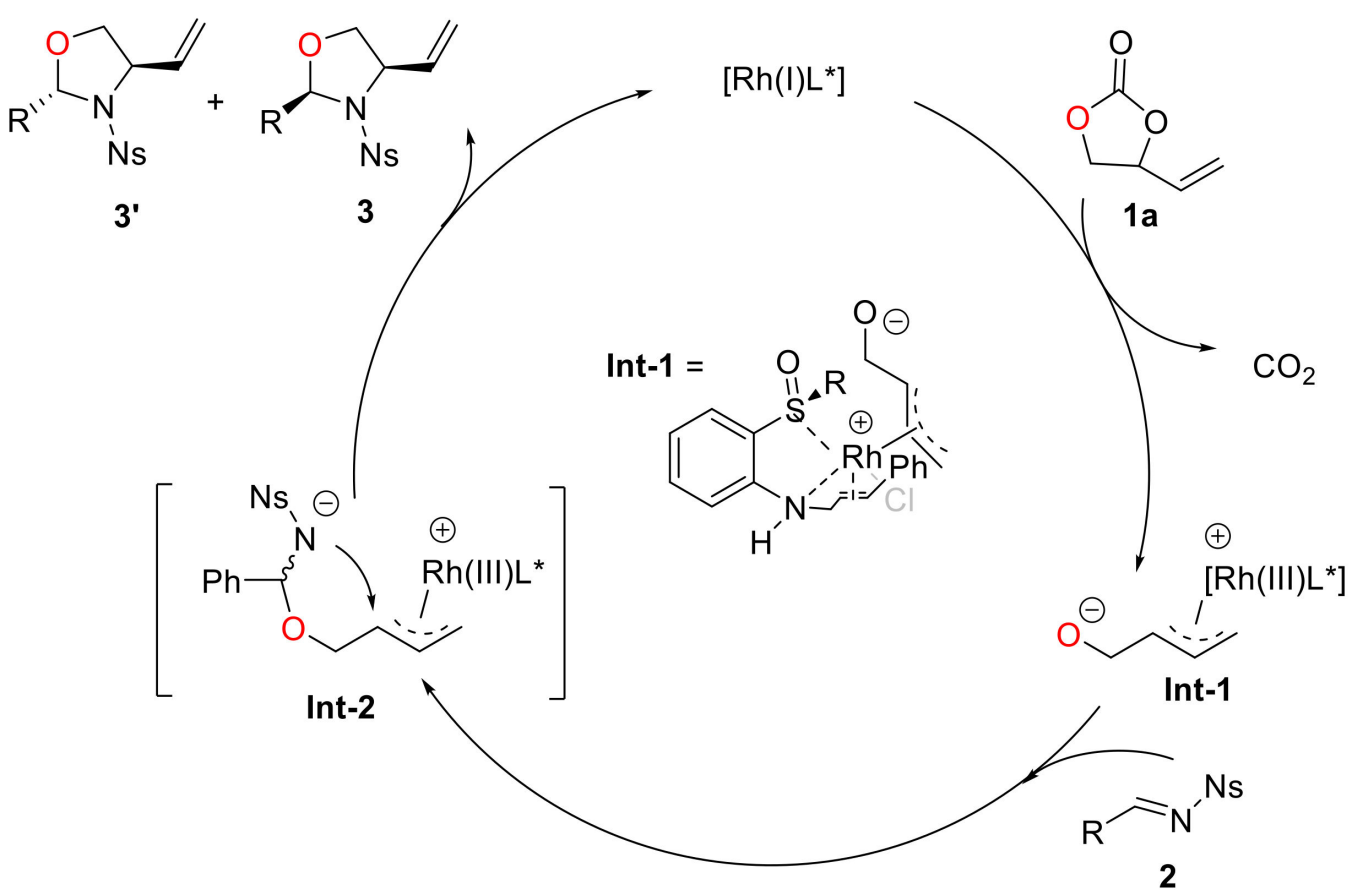
Possible mechanism. Note: L* represents chiral sulfoxide-*N*-olefin tridentate ligand.

Based on the previous reports [13,20], a possible mechanism was proposed. The reaction starts from the ring opening of VECs **1a** by oxidative addition with a Rh(I) catalyst with the assistance of the tridentate ligand. The formed π-allyl-Rh(III) intermediate(**Int-1**), which already recieved 18 valance electrons and no vacant coordination for imine, could only attack the imine by oxide anion. This is a possible reason for the low diastereoselectivity. Subsequential *N* anion after the addition continued to proceed nucleophilic attack back to Rh(allyl) intermediate **Int-2**, providing allylic oxazolidines and regenerating the Rh(I) catalyst to furnish the catalytic cycle (Scheme 4).

## 3. Materials and Methods

### 3.1. Reagents and General Methods

All manipulations were carried out under the argon atmosphere using standard Schlenk techniques. All glassware were oven or flame dried immediately prior to use. All solvents were purified and dried according to standard methods prior to use, unless stated otherwise. ^1^H NMR spectra were obtained at 400 MHz or 600 MHz and recorded relative to the tetramethylsilane signal (0 ppm) or residual protio-solvent (7.26 ppm for CDCl_3_, 1.94 ppm for CD_3_CN). ^13^C NMR spectra were obtained at 100 MHz or 150 MHz, and chemical shifts were recorded relative to the solvent resonance (CDCl_3_, 77.16 ppm, CD_3_CN, 1.32 ppm). Data for NMR are recorded as follows: chemical shift (δ, ppm), multiplicity (s = singlet, d = doublet, t = triplet, m = multiplet or unresolved, br = broad singlet, coupling constant(s) in Hz, integration). Infrared spectra were recorded on Nicolet FT-IR spectrometers. The accurate masses were measured by ESI-TOF using QTOF Ultima, G2-xs TOF from Waters (Milford, MA, USA), and microflex LRF MALDI-TOF. Optical rotations αD were obtained with AUTOPOL VI from rudolph-research-analytical. HPLC using chiral stationary phase columns by comparing the samples with the appropriate racemic samples, column and elution details specified in each entry.

### 3.2. Synthetic Procedures

General Procedure for the Synthesis of **3** and **3′**.

In a screw-cap Schlenk tube filled with argon, [Rh(C_2_H_4_)_2_Cl]_2_ (0.004 mmol, 2 mol%), Sulfoxide ligand **L1** (0.008 mmol, 4 mol%), *N*-nosylimine **2** (0.20 mmol) were added. after that, 4-Vinyl-1,3-dioxolan-2-one **1** (0.40 mmol) and 1,2-Dichloroethane (DCE) (2.0 mL) were added. Finally, triethylamine (0.30 mmol) was added. After 24 h stirring at −10 °C, the reaction mixture was filtered over silica (CH_2_Cl_2_) and concentrated under reduced pressure to afford the crude product. The crude residue was purified by flash column chromatography (petroleum ether/ethyl acetate) to give the desired products **3** and **3′**.

Representative **3** and **3′**

*(2R,4R)-3-((4-nitrophenyl)sulfonyl)-2-phenyl-4-vinyloxazolidine* (**3a**): White solid; **m.p.**: 101–104 °C; 36% yield (26.0 mg); **HPLC** ee: 87% [Daicel CHIRALPAK AD-H (0.46 cm × 25 cm); *n*-hexane/2-propanol = 90/10; flow rate = 1.0 mL/min; detection wavelength = 214 nm; t_R_ = 18.02 (major), 22.33 (minor) min]. **[α]^20^_D_** = +16.9 (c 1.0, CHCl_3_). **^1^H NMR** (600 MHz, CDCl_3_) δ 8.24 (d, *J* = 8.8 Hz, 2H), 7.79 (d, *J* = 8.8 Hz, 2H), 7.44 (d, *J* = 6.7 Hz, 2H), 7.38–7.32 (m, 3H), 6.21 (s, 1H), 5.76 (ddd, *J* = 17.9, 10.1, 8.0 Hz, 1H), 5.33 (d, *J* = 17.1 Hz, 1H), 5.23 (d, *J* = 10.2 Hz, 1H), 4.50 (q, *J* = 7.3 Hz, 1H), 4.10 (dd, *J* = 9.1, 7.0 Hz, 1H), 3.82 (dd, *J* = 9.1, 5.0 Hz, 1H). **^13^C NMR** (151 MHz, CDCl_3_) δ 150.1, 144.7, 136.6, 135.4, 129.5, 128.9, 128.6, 127.5, 124.2, 119.3, 92.5, 71.2, 62.0. **IR** (CH_2_Cl_2_): ν_max_ (cm^−1^) = 3055, 3005, 2972, 2875, 2304, 1715, 1530, 1347, 1274, 1262, 1171, 900, 767, 747, 735, 687. **HRMS** (ESI^+^) calcd for C_17_H_16_N_2_NaO_5_S [M + Na]^+^: 383.0672, Found: 383.0665.

*(2S,4R)-3-((4-nitrophenyl)sulfonyl)-2-phenyl-4-vinyloxazolidine* (**3a′**): White solid; **m.p.**: 101–104 °C; 29% yield (20.8 mg); **HPLC** ee: 98% [Daicel CHIRALPAK AD-H (0.46 cm × 25 cm); *n*-hexane/2-propanol = 90/10; flow rate = 1.0 mL/min; detection wavelength = 214 nm; t_R_ = 17.01 (major), 20.61 (minor) min]. **[α]^20^_D_** = −37.3 (c 1.0, CHCl_3_). **^1^H NMR** (400 MHz, CDCl_3_) δ 8.10 (d, *J* = 8.8 Hz, 2H), 7.49 (d, *J* = 8.8 Hz, 2H), 7.39–7.31 (m, 1H), 7.30–7.22 (m, 4H), 6.17 (s, 1H), 5.91 (ddd, *J* = 17.0, 10.1, 8.6 Hz, 1H), 5.42 (d, *J* = 17.0 Hz, 1H), 5.31 (d, *J* = 10.1 Hz, 1H), 4.44 (dt, *J* = 8.6, 5.7 Hz, 1H), 4.30 (dd, *J* = 8.7, 6.0 Hz, 1H), 3.86 (dd, *J* = 8.7, 5.4 Hz, 1H). **^13^C NMR** (101 MHz, CDCl_3_) δ 149.7, 146.1, 136.7, 134.4, 129.7, 128.4, 128.3, 128.1, 123.7, 119.3, 92.4, 71.7, 62.6. **IR** (CH_2_Cl_2_): ν_max_ (cm^−1^) = 3053, 3009, 2977, 2871, 2301, 1713, 1531, 1341, 1273, 1262, 1174, 901, 769, 750, 741, 681. **HRMS** (ESI^+^) calcd for C_17_H_16_N_2_NaO_5_S [M + Na]^+^: 383.0672, Found: 383.0665.

*(2R,4R)-2-(4-chlorophenyl)-3-((4-nitrophenyl)sulfonyl)-4-vinyloxazolidine* (**3b**): White solid; **m.p.**: 149–151 °C; 37% yield (29.1 mg); **HPLC** ee: 32% [Daicel CHIRALPAK AD-H (0.46 cm × 25 cm); *n*-hexane/2-propanol = 90/10; flow rate = 0.5 mL/min; detection wavelength = 214 nm; t_R_ = 39.58 (major), 46.06 (minor) min]. **[α]^20^_D_** = +14.8 (c 1.0, CHCl_3_). **^1^H NMR** (600 MHz, CDCl_3_) δ 8.32 (d, *J* = 8.8 Hz, 2H), 7.89 (d, *J* = 8.8 Hz, 2H), 7.42 (d, *J* = 8.3 Hz, 2H), 7.33 (d, *J* = 8.3 Hz, 2H), 6.20 (s, 1H), 5.70 (ddd, *J* = 17.5, 10.2, 7.7 Hz, 1H), 5.31 (d, *J* = 17.0 Hz, 1H), 5.23 (d, *J* = 10.2 Hz, 1H), 4.41 (q, *J* = 6.8 Hz, 1H), 4.06 (dd, *J* = 9.1, 7.1 Hz, 1H), 3.75 (dd, *J* = 9.3, 5.4 Hz, 1H). **^13^C NMR** (151 MHz, CDCl_3_) δ 150.4, 144.1, 135.6, 135.5, 135.0, 129.0, 128.8, 128.8, 124.4, 119.5, 91.7, 71.0, 61.9. **IR** (CH_2_Cl_2_): ν_max_ (cm^−1^) = 3105, 3064, 2963, 2925, 2872, 2298, 1763, 1603, 1536, 1350, 1265, 1174, 1118, 856, 757, 697, 632, 576. **HRMS** (ESI^+^) calcd for C_17_H_15_ClN_2_NaO_5_S [M + Na]^+^: 417.0282, Found: 417.0294.

*(2S,4R)-2-(4-chlorophenyl)-3-((4-nitrophenyl)sulfonyl)-4-vinyloxazolidine* (**3b′**): White solid; **m.p.**: 149–151 °C; 25% yield (19.7 mg); **HPLC** ee: 83% [Daicel CHIRALPAK AD-H (0.46 cm × 25 cm); *n*-hexane/2-propanol = 90/10; flow rate = 0.5 mL/min; detection wavelength = 214 nm; t_R_ = 42.76 (major), 54.28 (minor) min]. **[α]^20^_D_** = −22.5 (c 1.0, CHCl_3_).**^1^H NMR** (600 MHz, CDCl_3_) δ 8.19 (d, *J* = 8.5 Hz, 2H), 7.61 (d, *J* = 8.5 Hz, 2H), 7.26 (d, *J* = 2.2 Hz, 5H), 6.15 (s, 1H), 5.87–5.77 (m, 1H), 5.42 (d, *J* = 17.0 Hz, 1H), 5.30 (d, *J* = 10.0 Hz, 1H), 4.48–4.41 (m, 1H), 4.25 (dd, *J* = 8.9, 6.1 Hz, 1H), 3.84 (dd, *J* = 8.9, 5.2 Hz, 1H).**^13^C NMR** (151 MHz, CDCl_3_) δ 149.9, 146.1, 135.8, 135.5, 133.9, 129.4, 128.6, 128.6, 123.9, 119.9, 91.5, 71.6, 62.5. **IR** (CH_2_Cl_2_): ν_max_ (cm^−1^) = 3101, 3067, 2966, 2922, 2871, 2293, 1761, 1600, 1532, 1356, 1264, 1172, 1120, 851, 754, 699, 638, 577. **HRMS** (ESI^+^) calcd for C_17_H_15_ClN_2_NaO_5_S [M + Na]^+^: 417.0282, Found: 417.0294.

*(2R,4R)-3-((4-nitrophenyl)sulfonyl)-2-(p-tolyl)-4-vinyloxazolidine* (**3c**): White solid; **m.p.**: 124–126 °C; 36% yield (26.9 mg); **HPLC** ee: 21% [Daicel CHIRALPAK IA-H (0.46 cm × 25 cm); *n*-hexane/2-propanol = 95/5; flow rate = 1.0 mL/min; detection wavelength = 254 nm; t_R_ = 21.33 (major), 29.94 (minor) min]. **[α]^20^_D_** = +62.7 (c 1.0, CHCl_3_). **^1^H NMR** (600 MHz, CDCl_3_) δ 8.24 (d, *J* = 8.5 Hz, 2H), 7.79 (d, *J* = 8.5 Hz, 2H), 7.30 (d, *J* = 7.8 Hz, 2H), 7.12 (d, *J* = 7.8 Hz, 2H), 6.17 (s, 1H), 5.77 (ddd, *J* = 17.6, 10.1, 8.0 Hz, 1H), 5.33 (d, *J* = 17.0 Hz, 1H), 5.23 (d, *J* = 10.1 Hz, 1H), 4.48 (td, *J* = 7.4, 4.9 Hz, 1H), 4.08 (dd, *J* = 9.1, 7.0 Hz, 1H), 3.82 (dd, *J* = 9.1, 4.9 Hz, 1H), 2.35 (s, 3H). **^13^C NMR** (151 MHz, CDCl_3_) δ 150.1, 144.8, 139.6, 135.5, 133.6, 129.2, 128.9, 127.5, 124.1, 119.2, 92.5, 71.1, 61.9, 21.3. **IR** (CH_2_Cl_2_): ν_max_ (cm^−1^) = 3101, 3034, 2973, 2915, 2842, 2278, 1721, 1608, 1516, 1349, 1263, 1171, 876, 751, 687, 639. **HRMS** (ESI^+^) calcd for C_18_H_18_N_2_NaO_5_S [M + Na]^+^: 397.0829, Found: 397.08421.

*(2S,4R)-3-((4-nitrophenyl)sulfonyl)-2-(p-tolyl)-4-vinyloxazolidine* (**3c′**): White solid; **m.p.**: 124–126 °C; 24% yield (17.9 mg); **HPLC** ee: 85% [Daicel CHIRALPAK IA-H (0.46 cm × 25 cm); *n*-hexane/2-propanol = 95/5; flow rate = 1.0 mL/min; detection wavelength = 254 nm; t_R_ = 23.94 (minor), 25.75 (major) min]. **[α]^20^_D_** = −20.6 (c 1.0, CHCl_3_). **^1^H NMR** (600 MHz, CDCl_3_) δ 8.10 (d, *J* = 8.8 Hz, 2H), 7.51 (d, *J* = 8.8 Hz, 2H), 7.14 (d, *J* = 7.8 Hz, 2H), 7.03 (d, *J* = 7.7 Hz, 2H), 6.12 (s, 1H), 5.91 (dt, *J* = 18.1, 9.1 Hz, 1H), 5.42 (d, *J* = 17.0 Hz, 1H), 5.31 (d, *J* = 10.1 Hz, 1H), 4.45 (dt, *J* = 8.5, 5.8 Hz, 1H), 4.29 (dd, *J* = 8.8, 6.1 Hz, 1H), 3.85 (dd, *J* = 8.8, 5.4 Hz, 1H), 2.35 (s, 3H). **^13^C NMR** (151 MHz, CDCl_3_) δ 149.6, 146.2, 139.9, 134.5, 133.8, 128.9, 128.5, 128.1, 123.6, 119.3, 92.3, 71.7, 62.6, 21.3. **IR** (CH_2_Cl_2_): ν_max_ (cm^−1^) = 3110, 3031, 2979, 2911, 2848, 2281, 1722, 1608, 1522, 1350, 1269, 1172, 878, 752, 688, 644. **HRMS** (ESI^+^) calcd for C_18_H_18_N_2_NaO_5_S [M + Na]^+^: 397.0829, Found: 397.08421.

*(2R,4R)-2-([1,1′-biphenyl]-4-yl)-3-((4-nitrophenyl)sulfonyl)-4-vinyloxazolidine* (**3d**): White solid; **m.p.**: 140–143 °C; 29% yield (25.2 mg); **HPLC** ee: 55% [Daicel CHIRALPAK AD-H (0.46 cm × 25 cm); *n*-hexane/2-propanol = 90/10; flow rate = 1.0 mL/min; detection wavelength = 214 nm; t_R_ = 25.06 (major), 29.58 (minor) min]. **[α]^20^_D_** = +13.2 (c 1.0, CHCl_3_). **^1^H NMR** (600 MHz, CDCl_3_) δ 8.26 (d, *J* = 8.8 Hz, 2H), 7.84 (d, *J* = 8.8 Hz, 2H), 7.58–7.55 (m, 4H), 7.51 (d, *J* = 8.2 Hz, 2H), 7.46 (t, *J* = 7.6 Hz, 2H), 7.38 (t, *J* = 7.4 Hz, 1H), 6.26 (s, 1H), 5.84–5.77 (m, 1H), 5.36 (d, *J* = 16.9 Hz, 1H), 5.26 (d, *J* = 10.2 Hz, 1H), 4.50 (td, *J* = 7.6, 5.3 Hz, 1H), 4.12 (dd, *J* = 9.1, 7.0 Hz, 1H), 3.85 (dd, *J* = 9.2, 5.1 Hz, 1H). **^13^C NMR** (151 MHz, CDCl_3_) δ 150.2, 144.6, 142.5, 140.4, 135.6, 135.4, 129.0, 128.9, 128.0, 127.8, 127.3, 127.2, 124.2, 119.4, 92.3, 71.2, 62.0. **IR** (CH_2_Cl_2_): ν_max_ (cm^−1^) = 3109, 3064, 2993, 2911, 2815, 2235, 1712, 1635, 1509, 1356, 1234, 1163, 888, 788, 654, 616. **HRMS** (ESI^+^) calcd for C_23_H_20_N_2_NaO_5_S [M + Na]^+^: 459.0985, Found: 459.0990.

*(2S,4R)-2-([1,1′-biphenyl]-4-yl)-3-((4-nitrophenyl)sulfonyl)-4-vinyloxazolidine* (**3d′**): White solid; **m.p.**: 140–143 °C; 23% yield (20.0 mg); **HPLC** ee: 80% [Daicel CHIRALPAK AD-H (0.46 cm × 25 cm); *n*-hexane/2-propanol = 90/10; flow rate = 1.0 mL/min; detection wavelength = 214 nm; t_R_ = 23.81 (minor), 26.79 (major) min]. **[α]^20^_D_** = −8.9 (c 1.0, CHCl_3_). **^1^H NMR** (400 MHz, CDCl_3_) δ 8.11 (d, *J* = 8.8 Hz, 2H), 7.59–7.51 (m, 4H), 7.50–7.43 (m, 4H), 7.39 (t, *J* = 7.2 Hz, 1H), 7.35 (d, *J* = 8.2 Hz, 2H), 6.21 (s, 1H), 5.97–5.87 (m, 1H), 5.44 (d, *J* = 17.1 Hz, 1H), 5.33 (d, *J* = 10.1 Hz, 1H), 4.49 (dt, *J* = 8.6, 5.7 Hz, 1H), 4.33 (dd, *J* = 8.7, 6.1 Hz, 1H), 3.89 (dd, *J* = 8.8, 5.4 Hz, 1H). **^13^C NMR** (101 MHz, CDCl_3_) δ 149.8, 146.2, 142.9, 140.2, 135.6, 134.4, 129.1, 128.6, 128.5, 128.0, 127.2, 127.0, 123.8, 119.4, 92.2, 71.8, 62.7. **IR** (CH_2_Cl_2_): ν_max_ (cm^−1^) = 3109, 3060, 2999, 2910, 2819, 2232, 1721, 1633, 1505, 1353, 1234, 1161, 881, 785, 659, 610. **HRMS** (ESI^+^) calcd for C_23_H_20_N_2_NaO_5_S [M + Na]^+^: 459.0985, Found: 459.0990.

*(2R,4R)-2-(naphthalen-1-yl)-3-((4-nitrophenyl)sulfonyl)-4-vinyloxazolidine* (**3e**): White solid; **m.p.**: 127–129 °C; 28% yield (22.9 mg); **HPLC** ee: 97% [Daicel CHIRALPAK IC-H (0.46 cm × 25 cm); *n*-hexane/2-propanol = 90/10; flow rate = 1.0 mL/min; detection wavelength = 214 nm; t_R_ = 32.07 (major), 37.52 (minor) min]. **[α]^20^_D_** = +28.1 (c 1.0, CHCl_3_). **^1^H NMR** (400 MHz, CDCl_3_) δ 8.23 (t, *J* = 9.0 Hz, 3H), 7.93–7.79 (m, 4H), 7.67 (d, *J* = 7.1 Hz, 1H), 7.58–7.47 (m, 2H), 7.42 (t, *J* = 7.7 Hz, 1H), 6.88 (s, 1H), 6.06 (ddd, *J* = 17.0, 10.2, 8.1 Hz, 1H), 5.49 (d, *J* = 17.1 Hz, 1H), 5.40 (d, *J* = 10.2 Hz, 1H), 4.56 (q, *J* = 7.1 Hz, 1H), 4.15 (dd, *J* = 9.2, 7.0 Hz, 1H), 3.76 (dd, *J* = 9.2, 6.5 Hz, 1H). **^13^C NMR** (101 MHz, CDCl_3_) δ 150.5, 143.9, 138.1, 134.8, 133.9, 132.4, 129.0, 127.6, 126.1, 125.2, 124.5, 124.0, 119.7, 91.0, 71.1, 62.0. **IR** (CH_2_Cl_2_): ν_max_ (cm^−1^) = 3111, 3054, 2983, 2922, 2831, 2286, 1729, 1611, 1519, 1346, 1269, 1183, 877, 793, 691, 613. **HRMS** (ESI^+^) calcd for C_21_H_18_N_2_NaO_5_S [M + Na]^+^: 433.0829, Found: 433.0835.

*(2S,4R)-2-(naphthalen-1-yl)-3-((4-nitrophenyl)sulfonyl)-4-vinyloxazolidine* (**3e′**): White solid; **m.p.**: 127–129 °C; 27% yield (22.1 mg); **HPLC** ee: 41% [Daicel CHIRALPAK IC-H (0.46 cm × 25 cm); *n*-hexane/2-propanol = 90/10; flow rate = 1.0 mL/min; detection wavelength = 214 nm; t_R_ = 48.99 (minor), 52.06 (major) min]. **[α]^20^_D_** = −42.4 (c 1.0, CHCl_3_). **^1^H NMR** (400 MHz, CDCl_3_) δ 8.06–7.99 (m, 1H), 7.95 (d, *J* = 8.6 Hz, 2H), 7.86 (d, *J* = 8.2 Hz, 1H), 7.82–7.76 (m, 1H), 7.66 (d, *J* = 7.1 Hz, 1H), 7.55 (d, *J* = 8.7 Hz, 2H), 7.48–7.41 (m, 3H), 6.75 (s, 1H), 5.99–5.84 (m, 1H), 5.52 (d, *J* = 17.0 Hz, 1H), 5.34 (d, *J* = 10.1 Hz, 1H), 4.82–4.73 (m, 1H), 4.34 (dd, *J* = 9.0, 6.3 Hz, 1H), 3.92 (dd, *J* = 9.0, 4.1 Hz, 1H). **^13^C NMR** (101 MHz, CDCl_3_) δ 149.5, 145.5, 134.9, 133.9, 131.0, 130.7, 130.6, 128.8, 128.6, 127.7, 126.6, 126.0, 124.7, 123.6, 123.3, 119.5, 91.2, 71.1, 62.8. **IR** (CH_2_Cl_2_): ν_max_ (cm^−1^) = 3108, 3052, 2988, 2929 2830, 2288, 1722, 1610, 1522, 1349, 1266, 1190, 870, 791, 694, 615. **HRMS** (ESI^+^) calcd for C_21_H_18_N_2_NaO_5_S [M + Na]^+^: 433.0829, Found: 433.0835.

*(2R,4R)-2-(3-bromophenyl)-3-((4-nitrophenyl)sulfonyl)-4-vinyloxazolidine* (**3f**): Pale yellow solid; **m.p.**: 117–119 °C; 34% yield (29.7 mg); **HPLC** ee: 81% [Daicel CHIRALPAK AD-H (0.46 cm × 25 cm); *n*-hexane/2-propanol = 90/10; flow rate = 1.0 mL/min; detection wavelength = 254 nm; t_R_ = 21.37 (major), 24.18(minor) min]. **[α]^20^_D_** = + 24.1 (c 1.0, CHCl_3_). **^1^H NMR** (600 MHz, CDCl_3_) δ 8.31 (d, *J* = 8.6 Hz, 2H), 7.86 (d, *J* = 8.6 Hz, 2H), 7.51 (s, 1H), 7.48 (d, *J* = 8.0 Hz, 1H), 7.42 (d, *J* = 7.7 Hz, 1H), 7.24 (t, *J* = 7.8 Hz, 1H), 6.16 (s, 1H), 5.78–5.69 (m, 1H), 5.35 (d, *J* = 17.0 Hz, 1H), 5.27 (d, *J* = 10.2 Hz, 1H), 4.46 (q, *J* = 6.9 Hz, 1H), 4.14–4.07 (m, 1H), 3.78 (dd, *J* = 9.0, 5.4 Hz, 1H). **^13^C NMR** (151 MHz, CDCl_3_) δ 150.3, 144.2, 139.1, 135.0, 132.6, 130.3, 130.2, 128.9, 126.3, 124.3, 122.7, 119.6, 91.5, 71.2, 62.0. **IR** (CH_2_Cl_2_): ν_max_ (cm^−1^) = 3120, 3033, 2977, 2918, 2825, 2274, 1719, 1610, 1515, 1342, 1266, 1188, 870, 799, 684, 609. **HRMS** (ESI^+^) calcd for C_17_H_15_BrN_2_NaO_5_S [M + Na]^+^: 460.9777, Found: 460.9787.

*(2S,4R)-2-(3-bromophenyl)-3-((4-nitrophenyl)sulfonyl)-4-vinyloxazolidine* (**3f′**): Pale yellow solid; **m.p.**: 117–119 °C; 29% yield (25.3 mg); **HPLC** ee: 75% [Daicel CHIRALPAK AD-H (0.46 cm × 25 cm); *n*-hexane/2-propanol = 90/10; flow rate = 1.0 mL/min; detection wavelength = 254 nm; t_R_ = 17.63 (major), 26.39 (minor) min]. **[α]^20^_D_** = −4.5 (c 1.0, CHCl_3_).**^1^H NMR** (400 MHz, CDCl_3_) δ 8.19 (d, *J* = 8.3 Hz, 2H), 7.56 (d, *J* = 8.4 Hz, 2H), 7.51–7.44 (m, 1H), 7.38–7.31 (m, 1H), 7.27–7.13 (m, 2H), 6.11 (s, 1H), 6.01–5.87 (m, 1H), 5.47 (d, *J* = 17.0 Hz, 1H), 5.36 (d, *J* = 10.1 Hz, 1H), 4.49 (dt, *J* = 9.0, 5.5 Hz, 1H), 4.31 (dd, *J* = 8.9, 5.7 Hz, 1H), 3.89 (dd, *J* = 8.9, 5.3 Hz, 1H). **^13^C NMR** (101 MHz, CDCl_3_) δ 149.9, 145.9, 138.8, 134.2, 132.8, 130.8, 130.0, 128.3, 127.3, 123.9, 122.5, 119.6, 91.4, 71.8, 62.7. **IR** (CH_2_Cl_2_): ν_max_ (cm^−1^) = 3117, 3029, 2973, 2918, 2820, 2277, 1720, 1611, 1519, 1346, 1262, 1189, 871, 7990, 682, 610. **HRMS** (ESI^+^) calcd for C_17_H_15_BrN_2_NaO_5_S [M + Na]^+^: 460.9777, Found: 460.9787.

## 4. Conclusions

In conclusion, we have utilized rhodium/tridentate sulfoxide-*N*-olefin hybrid Ligand catalyzed asymmetric allylic substitution to furnish a tandem reaction of VECs and various substituted N-nosylimines. Chiral oxazolidines diastereomers as (3 + 2) cycloadducts were synthesized in good to high yield, with moderate to excellent enantioselectivity. The outcome for the low diastereoselectivity is discussed, along with a proposed mechanism. More tandem reaction based on these results are undergoing in our lab.

## Figures and Tables

**Table 1:** Optimization Study ^a^.

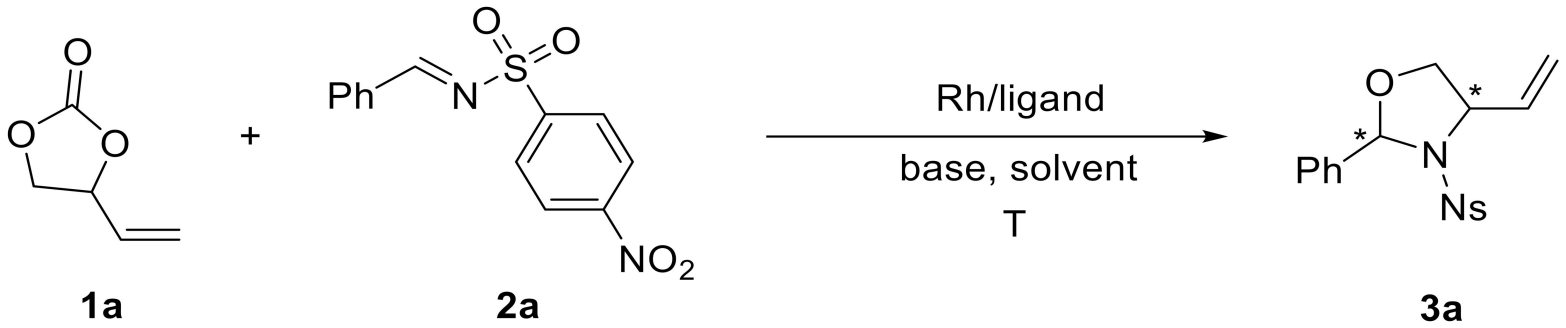								
Entry	Rh	L	Base	Solvent	T (°C)	Yield ^b^ (%)	ee ^c^ (%)	dr ^d^
1	[Rh(C_2_H_4_)_2_Cl]_2_	**L1**	Cs_2_CO_3_	DCM	rt	32	41/37	1.1/1
2	[Rh(C_2_H_4_)_2_Cl]_2_	**L1**	Cs_2_CO_3_	Tol.	rt	9	50/51	1.8/1
3	[Rh(C_2_H_4_)_2_Cl]_2_	**L1**	Cs_2_CO_3_	THF	rt	47	12/23	1.1/1
4	[Rh(C_2_H_4_)_2_Cl]_2_	**L1**	Cs_2_CO_3_	MeCN	rt	24	16/31	2/1
5	[Rh(C_2_H_4_)_2_Cl]_2_	**L1**	Cs_2_CO_3_	DCE	rt	35	53/73	1.2/1
6	[Rh(C_2_H_4_)_2_Cl]_2_	**L1**	CsF	DCE	rt	24	60/77	2/1
7	[Rh(C_2_H_4_)_2_Cl]_2_	**L1**	K_2_CO_3_	DCE	rt	29	31/35	2.2/1
8	[Rh(C_2_H_4_)_2_Cl]_2_	**L1**	CsCl	DCE	rt	19	53/51	1.5/1
9	[Rh(C_2_H_4_)_2_Cl]_2_	**L1**	Et_3_N	DCE	rt	65	45/89	1.2/1
10	[Rh(C_2_H_4_)_2_Cl]_2_	**L1**	DBU	DCE	rt	61	23/25	2/1
11	[Rh(C_2_H_4_)_2_Cl]_2_	**L1**	TMEDA	DCE	rt	39	65/69	1.4/1
12	[Rh(C_2_H_4_)_2_Cl]_2_	**L1**	Et_3_N	DCE	0	65	60/88	1.2/1
13	[Rh(C_2_H_4_)_2_Cl]_2_	**L1**	Et_3_N	DCE	−10	61	81/91	1.2/1
14	[Rh(C_2_H_4_)_2_Cl]_2_	**L1**	Et_3_N	DCE	−25	52	57/91	1.2/1
15	Rh(acac)(C_2_H_4_)_2_	**L1**	Et_3_N	DCE	−10	42	15/65	2/1
16	[Rh(cod)Cl]_2_	**L1**	Et_3_N	DCE	−10	nr	-	-
17 ^e^	[Rh(C_2_H_4_)_2_Cl]_2_	**L1**	Et_3_N	DCE	−10	65	87/98	1.2/1
18	[Rh(C_2_H_4_)_2_Cl]_2_	**L2**	Et_3_N	DCE	−10	32	7/5	2/1
19	[Rh(C_2_H_4_)_2_Cl]_2_	**L3**	Et_3_N	DCE	−10	26	7/9	1.4/1
20	[Rh(C_2_H_4_)_2_Cl]_2_	**L4**	Et_3_N	DCE	−10	25	rac	1.7/1
21	[Rh(C_2_H_4_)_2_Cl]_2_	**L5**	Et_3_N	DCE	−10	51	59/61	1.4/1
22	[Rh(C_2_H_4_)_2_Cl]_2_	**L6**	Et_3_N	DCE	−10	66	33/37	1.5/1

^a^ Reaction conditions: Rh salt (0.001 mmol), ligand (0.002 mmol), **1a** (0.20 mmol), **2a** (0.10 mmol), base (0.15 mmol), and solvent (1 mL). ^b^ Isolated yield. ^c^ Determined by a chiral HPLC. ^d^ Determined by ^1^H NMR analysis of the crude product. ^e^ Rh salt (0.002 mmol), ligand (0.004 mmol). Note: The carbon with * represents a chiral center.

## Data Availability

Data are contained within the article and Supplementary Materials.

## Supplementary Materials

The following supporting information can be downloaded at: https://www.mdpi.com/article/10.3390/molecules29051019/s1, Experimental details, NMR spectra, X-ray Crystallographic Information, HPLC Spectra, HRMS data for new products. Refs. [43,44,45,46,47] are cited in Supplementary Materials.
