# Supplementary material for: Rhodium-Catalyzed Tandem Asymmetric Allylic Decarboxylative Addition and Cyclization of Vinylethylene Carbonates with N-Nosylimines
Source: Molecules. 2024 Feb 26;29(5):1019. doi: 10.3390/molecules29051019 (PMC10934018; doi:10.3390/molecules29051019)
Supplement: Supplementary file 1 [file molecules-29-01019-s001.zip › molecules-2862249-supplementary.pdf]

Supporting Information

Rhodium-Catalyzed Tandem Asymmetric Allylic Decarboxylative Addition and Cyclization of Vinylethylene Carbonates with N-Nosylimines

Xiao-Lin Wang, Hai-Bin Jiang, Sheng-Cai Zheng * and Xiao-Ming Zhao *

School of Chemical Science and Engineering, Tongji University, 1239 Siping Road, Shanghai 200092, China; 1810923@tongji.edu.cn (X.-L.W.); haibin@tongji.edu.cn (H.-B.J.)

* Correspondence: zhengsc@tongji.edu.cn (S.-C.Z.); xmzhao08@mail.tongji.edu.cn (X.-M.Z.)

Table of Contents

1. General S2
2. Preparation of Ligand **L1-L5** S2
3. General procedures for the synthesis of *N*-nosylimine **2** S6
4. General procedure for the synthesis of **3** S6
5. General procedure for the synthesis of **4** and **5** S12
6. X-ray crystallographic information of **3b** S13
7. Copies of NMR spectra of compounds **L1-L6, 3/3'**, **4** and **5** S15
8. HPLC Spectra of compounds **3/3'**, **4** and **5** S51
9. References S73

1. General

All manipulations were carried out under the argon atmosphere using standard Schlenk techniques. All glassware was oven or flame dried immediately prior to use. All solvents were purified and dried according to standard methods prior to use, unless stated otherwise.

^{1}H NMR spectra were obtained at 400 MHz or 600 MHz and recorded relative to the tetramethylsilane signal (0 ppm) or residual protio-solvent (7.26 ppm for $CDCl_3$, 1.94 ppm for CD_3CN). ^{13}C NMR spectra were obtained at 101 MHz or 151 MHz, and chemical shifts were recorded relative to the solvent resonance ($CDCl_3$, 77.16 ppm, CD_3CN, 1.32 ppm). Data for NMR are recorded as follows: chemical shift (δ, ppm), multiplicity (s = singlet, d = doublet, t = triplet, m = multiplet or unresolved, br = broad singlet, coupling constant(s) in Hz, integration). Infrared spectra were recorded on Nicolet FT-IR spectrometers. The accurate masses were measured by ESI-TOF using QTOF Ultima, G2-xs TOF from Waters, and microflex LRF MALDI-TOF. Optical rotations αD were obtained with AUTOPOL VI from rudolph-research-analytical. HPLC using chiral stationary phase columns by comparing the samples with the appropriate racemic samples, column and elution details specified in each entry.

2. Preparation of Ligand L1-L5

1) Procedure for the synthesis of chiral ligands **L1**

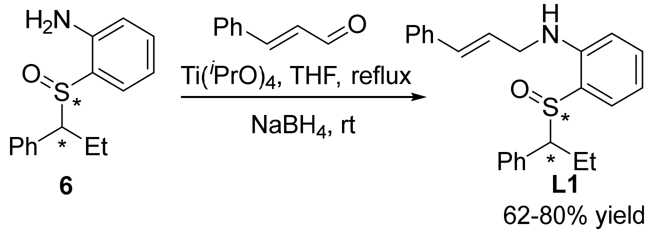

An oven-dried round-bottomed flask equipped with a magnetic stir bar was charged with **6**[1] (0.5 mmol), α, β-unsaturated aldehyde (0.6 mmol, 1.2 equiv.), Ti(iPrO)$_4$ (1.0 mmol, 2.0 equiv.) and anhydrous tetrahydrofuran (5.0 mL) under argon atmosphere. The solution was refluxed in an oil bath for 2h and successively cooled down to room temperature. Then $NaBH_4$ (2.0 mmol, 4.0 equiv.) was added into the mixture. After stirring for 2h, the reaction was quenched with MeOH and H_2O. The organic layer was extracted by ethyl acetate, dried over anhydrous sodium sulfate, filtered, and concentrated in vacuo. The crude residue was purified by flash column chromatography (PE/EA = 10/1) to provide **L1** in 75% yield (140.7 mg).

***N*-Cinnamyl-2-((*S*)-((*S*)-1-phenylpropyl)sulfinyl)aniline** (**L1**): White solid; m.p. 105.0 – 105.6 °C; 75% yield (140.7 mg); 99% *ee*. **[α]$_D^{20}$** = -97.7° (c 1.0, $CHCl_3$). **HPLC**: [Daicel

CHIRALCEL AD-H (0.46 cm x 25 cm); hexane/2-propanol = 90/10; flow rate = 1.2 mL/min; detection wavelength = 214 nm; t_R = 11.286 (major), 21.328(minor) min]. **^{1}H NMR** (600 MHz, $CDCl_3$) δ 7.35 (d, *J* = 7.4 Hz, 2H), 7.33 – 7.27 (m, 5H), 7.26 – 7.21 (m, 2H), 7.18 – 7.12 (m, 2H), 7.03 (d, *J* = 7.4 Hz, 1H), 6.66 – 6.57 (m, 2H), 6.53 (d, *J* = 16.0 Hz, 1H), 6.30 (s, 1H), 6.15 (dt, *J* = 15.8, 5.4 Hz, 1H), 4.10 (dd, *J* = 9.5, 6.2 Hz, 1H), 3.79 – 3.66 (m, 2H), 2.09 – 1.95 (m, 2H), 0.90 (t, *J* = 7.3 Hz, 3H). **^{13}C NMR** (100 MHz, $CDCl_3$) δ 149.5, 136.9, 133.9, 132.5, 131.2, 129.7, 128.6, 128.4, 128.3, 127.8, 127.6, 126.5, 126.4, 119.7, 115.6, 111.9, 69.6, 45.2, 22.5, 11.9. **IR** (KBr): ν_{max} (cm^{-1}) = 3260, 3062, 3006, 2992, 2969, 2882, 1590, 1489, 1293, 1120, 1045, 745, 650. **HRMS** (ESI+) calcd for $C_{24}H_{25}NNaOS$ $[M+Na]^+$: 398.1549, Found: 398.1554.

2) Procedure for the synthesis of chiral ligands **L2**[2]

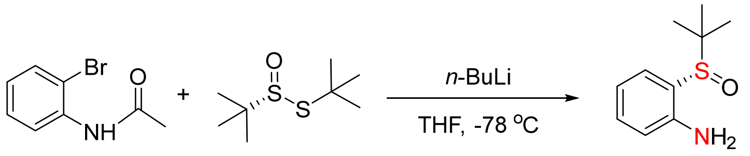

L2

A stirred solution of 2'-bromoacetanilide (428mg, 2 mmol, 1 equiv.) in 3 mL of anhydrous THF was cooled to −78 °C. To this solution was added *n*-BuLi (2.8 mL, 2.2 mmol, 1.55 M in hexane, 2.2 equiv.) dropwise while maintaining the temperature below −70 °C. The resulting yellow mixture was stirred at −78 °C during 2 h, before slow addition of a solution of 2-{[(*R*)-tert-butylsulfinyl]sulfanyl}-2-methylpropane (426 mg, 1.1 mmol, 1.1 equiv.) in 1 mL of anhydrous THF. The resulting strong yellow mixture was further stirred 1h at −78 °C. MeOH (few drops) was added. The mixture was allowed to warm to 0 °C and sat. NH_4Cl saturated solution (3 mL) was added. The mixture was extracted with diethyl ether (5 mL), washed with brine (3 mL), dried (Na_2SO_4), filtered off and evaporated under reduced pressure. The crude material was dissolved in 3 mL of EtOH. KOH (solution in water) (560 mg, 5 mmol, 5 equiv.) was added and the mixture was stirred in a sealed vial overnight at 85 °C. The orange solution was cooled to room temperature and ethanol was evaporated under reduced pressure. Diethyl ether (5 mL) and water (5 mL) were added. The organic layer was extracted, washed with brine (5 mL), dried (Na_2SO_4), filtered off and evaporated under reduced pressure. The crude was purified by column chromatography on silica gel with Petroleum/EtOAc (10/1) to afford **L2** (224 mg, 59 % yield).

(*S*)-2-(tert-Butylsulfinyl) aniline (**L2**):

Yellow solid; 59% yield; *ee*: 99%. **$[\alpha]_D^{20}$** = +58.9 (c 1.0, $CHCl_3$). **HPLC:** [Daicel CHIRALCEL OD-H (0.46 cm × 25 cm); *n*-hexane/2-propanol = 90/10; flow rate = 1.0 mL/min; detection wavelength = 214 nm; t_R = 11.39 (major), 13.28 (minor) min]. **^{1}H NMR (600 MHz,** $CDCl_3$**)** δ 7.19 (t, *J* = 8.3 Hz, 1H), 7.08 (d, *J* = 6.9 Hz, 1H), 6.69 (t, *J* = 7.4 Hz, 1H), 6.61 (d, *J* = 8.2 Hz, 1H), 5.15 (s, 2H), 1.29 (s, 9H). **^{13}C NMR** (150 MHz, $CDCl_3$**)** δ 149.4, 131.8, 128.9, 117.8, 117.6, 116.4, 58.8, 23.5. **IR** (KBr): ν_{max} (cm^{-1}) = 3411, 3143, 3005, 2986, 1642, 1614, 1401, 1281, 1263, 767, 752. **HRMS** (ESI^+) calcd for $C_{10}H_{15}NNaOS$ $[M+Na]^+$: 220.0767, Found: 220.0772.

3) Procedure for the synthesis of chiral ligands **L3**

L3 was similarly prepared from the general procedure for preparing **L1**.

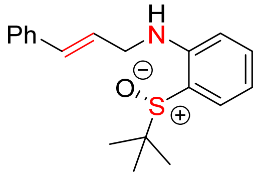

(*S*)-2-(tert-Butylsulfinyl)-*N*-cinnamylaniline (**L3**): Pale yellow wax; 36% yield (22.5 mg); *ee*: 97%. **[α]$_D^{20}$** = -28.8 (c 1.0, $CHCl_3$). **HPLC:** [Daicel CHIRALCEL OD-H (0.46 cm × 25 cm); *n*-hexane/2-propanol = 90/10; flow rate = 1.0 mL/min; detection wavelength = 254 nm; t_R = 11.42 (minor), 13.94 (major) min]. **^{1}H NMR** (400 MHz, $CDCl_3$) δ 7.41 – 7.36 (m, 2H), 7.36 – 7.29 (m, 2H), 7.29 – 7.21 (m, 2H), 7.18 – 7.11 (m, 1H), 7.08 (dd, *J* = 7.7, 1.6 Hz, 1H), 6.71 (dd, *J* = 8.4, 1.1 Hz, 1H), 6.69 – 6.58 (m, 2H), 6.33 – 6.23 (m, 1H), 3.95 (t, *J* = 5.4 Hz, 2H), 1.33 (s, 9H). **^{13}C NMR** (100 MHz, $CDCl_3$) δ 150.8, 136.8, 132.3, 131.2, 129.1, 128.5, 127.4, 126.5, 126.3, 116.6, 114.9, 112.1, 58.8, 45.2, 23.7. **IR** (KBr): ν_{max} (cm^{-1}) = 3410, 3122, 3001, 2917, 1634, 1621, 1411, 1271, 1253, 759, 747. **HRMS** (ESI^+) calcd for $C_{19}H_{23}NNaOS$ [M+Na]$^+$: 336.1393, Found: 336.1380.

4) Procedure for the synthesis of chiral ligands **L4**[3]

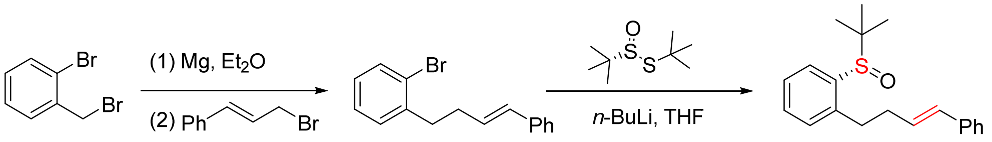

L4

In a three necked flask equipped with a condenser and a dropping funnel are introduced: magnesium (0.389 g; 15.8 mmol), Et_2O (12 mL) and one crystal of I_2. From the dropping funnel was added slowly (a small part and when the reaction started all the solution) 2-bromobenzyl bromide (2.988 g; 11.7 mmol) dissolved in Et_2O (12 mL). The mixture was refluxed for 1h 15 min. Under nitrogen, the Grignard reagent was transferred with a canula to a solution of cinnamyl bromide (2.400 g; 11.8 mmol) in THF (12 mL). After refluxing 2 h 45 min, the reaction mixture was hydrolyzed (HCl 10%), and extracted with Et_2O. The combined extracts were washed with water, dried ($MgSO_4$) and concentrated. The crude residue was purified by flash column chromatography (hexane) to provide (*E*)-1-bromo-2-(4-phenylbut-3-en-1-yl) benzene. The (*E*)-1-bromo-2-(4-phenylbut-3-en-1-yl) benzene (1.1 mmol) is then dissolved in anhydrous tetrahydrofuran (1.5 mL) and the reaction mixture is placed in a low-temperature stirring reactor and cooled to -78 °C. Then, n-butyllithium (2.2 mmol, 1.55 M in hexane) was slowly added to the reaction mixture, and after stirring for 30 minutes, (*R*) -*tert*-butylsulfothiobutyl (1 mmol) dissolved I n 1mL anhydrous tetrahydrofuran was slowly added to the reaction mixture. Continue stirring at -78 °C for 1h. After the reaction, the temperature was slowly raised to 0 °C, the saturated ammonium chloride solution was added, the aqueous phase was extracted by ethyl acetate, the saturated sodium chloride aqueous solution was washed, the organic phase was separated, the anhydrous sodium sulfate was dried, the concentration was reduced pressure, and the crude product was

purified by silica gel column chromatography (pure petroleum ether) to obtain compounds **L4** (84.2 mg, 27 %).

(*S*)-1-(tert-Butylsulfinyl)-2-(4-phenylbut-3-en-1-yl) benzene (**L4**): Chromatography (PE/EA = 20/1). Color-free wax; 30% yield (18.7 mg); *ee*: 96%. **[α]**$_D^{20}$ = +118.2 (c 1.0, $CHCl_3$). **HPLC**: [Daicel CHIRALPAK AD-H (0.46 cm × 25 cm); *n*-hexane/2-propanol = 90/10; flow rate = 1.0 mL/min; detection wavelength = 254 nm; t_R = 8.64 (minor), 10.98 (major) min]. **^{1}H NMR** (400 MHz, $CDCl_3$) δ δ 7.89 – 7.82 (m, 1H), 7.43 – 7.39 (m, 2H), 7.39 – 7.35 (m, 2H), 7.35 – 7.31 (m, 3H), 7.28 – 7.19 (m, 1H), 6.42 (d, *J* = 15.6 Hz, 1H), 6.26 – 6.19 (m, 1H), 3.15 – 3.08 (m, 1H), 2.76 – 2.69 (m, 1H), 2.66 – 2.59 (m, 1H), 2.57 – 2.47 (m, 1H), 1.24 (s, 9H). **^{13}C NMR** (150 MHz, $CDCl_3$) δ 141.2, 138.5, 137.3, 131.1, 131.0, 129.1, 128.9, 128.5, 127.1, 126.6, 126.5, 126.0, 57.5, 34.4, 32.4, 23.0. **IR** (KBr): ν_{max} (cm^{-1}) = 3428, 3131, 3010, 2911, 1633, 1612, 1422, 1268, 1249, 758, 746. **HRMS** (ESI$^+$) calcd for $C_{20}H_{25}OS$ [M+H]$^+$: 313.1621, Found: 313.1617.

5) Procedure for the synthesis of chiral ligands **L5**

L5 was prepared from the procedure for preparing **L3** without reduction step**.**

(1*E*, 2*E*)-N-(2-((*R*)-tert-butylsulfinyl) phenyl)-3-phenylprop-2-en-1-imine (L5)

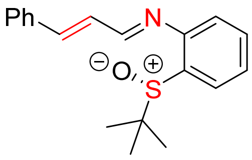

L5

Yellow solid; 87% yield (54.1 mg); **HPLC** *ee*: 99% [Daicel CHIRALCEL OD-H (0.46 cm × 25 cm); *n*-hexane/2-propanol = 90/10; flow rate = 1.0 mL/min; detection wavelength = 214 nm; t_R = 15.58 (minor), 22.29 (major) min]. **[α]**$_D^{20}$ = -31.1 (c 1.0, $CHCl_3$). **^{1}H NMR** (400 MHz, $CDCl_3$) δ 8.25 (d, J = 8.0 Hz, 1H), 7.94 (dd, J = 7.8, 1.6 Hz, 1H), 7.61 – 7.56 (m, 2H), 7.50 (td, J = 7.6, 1.6 Hz, 1H), 7.45 – 7.38 (m, 4H), 7.23 – 7.14 (m, 2H), 7.06 (dd, J = 7.8, 1.2 Hz, 1H), 1.20 (s, 9H). ^{13}C **NMR** (151 MHz, $CDCl_3$) δ 162.6, 150.3, 145.4, 135.3, 135.2, 131.8, 130.0, 128.9, 128.1, 127.7, 127.0, 126.2, 117.4, 58.3, 23.4. **IR** (KBr): ν_{max} (cm^{-1}) = 3543, 3111, 3012, 2912, 1643, 1611, 1421, 1235, 1224, 752, 741. **HRMS** (ESI$^+$) calcd for $C_{19}H_{21}NNaOS$ [M+Na]$^+$: 334.1236, Found: 334.1238.

6) Procedure for the synthesis of chiral ligands **L6**

L6 was prepared from the procedure for preparing **L1** without reduction step**.**

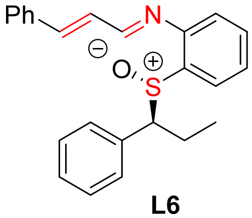

L6

(2*E*)-3-Phenyl-N-(2-((*R*)-((*R*)-1-phenylpropyl) sulfinyl) phenyl) prop-2-en-1-imine (**L6**): Yellow wax; 47% yield (35.1 mg); **[α]**$_D^{20}$ = +0.19 (c 1.0, $CHCl_3$). **^{1}H NMR** (600 MHz,

$CDCl_3$) δ 8.32 (d, *J* = 8.9 Hz, 1H), 7.63 – 7.57 (m, 2H), 7.43 (t, *J* = 7.2 Hz, 2H), 7.40 (d, *J* = 7.0 Hz, 1H), 7.30 (td, *J* = 7.5, 1.7 Hz, 1H), 7.28 – 7.23 (m, 1H), 7.13 (dd, *J* = 15.6, 8.4 Hz, 2H), 7.07 (t, *J* = 7.4 Hz, 2H), 7.04 – 6.96 (m, 3H), 6.80 (d, *J* = 7.2 Hz, 2H), 4.06 (t, *J* = 7.8 Hz, 1H), 2.46 (dt, *J* = 14.4, 7.3 Hz, 1H), 2.24 – 2.13 (m, 1H), 1.15 (t, *J* = 7.4 Hz, 3H). **^{13}C NMR** (150 MHz, $CDCl_3$) δ 161.8, 147.2, 145.6, 136.7, 135.2, 133.4, 130.9, 130.1, 129.0, 128.9, 128.1, 127.7, 127.5, 127.3, 126.3, 125.6, 116.2, 67.5, 23.3, 12.3. **IR** (KBr): ν_{max} (cm^{-1}) = 747, 757, 1226, 1233, 1471 1615, 1698, 2915, 3113, 3233, 3578. **HRMS** (ESI^+) calcd for $C_{24}H_{23}NNaOS$ [M+Na] $^+$: 396.1393, Found: 396.1384.

3. General procedures for the synthesis of N-nosylimine 2[4]

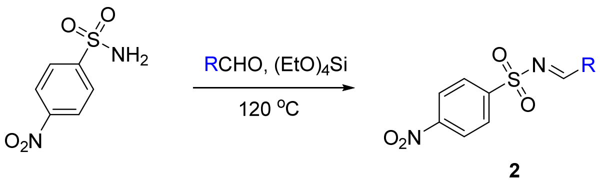

Amixture of 4-nitrobezenesulfonamide (5 mmol), aldehyde (5.5 mmol) and tetraethyl orthosilicate (5.5 mmol) was heated in flask equipped with Dean-Star at 120 ºC for 12h. After cooling at room temperature, the product was crystallized in EtOAc/heptane mixture. The crystals were collected by filtration, washed with pentane and dried under vacuum.

4. General procedure for the synthesis of 3

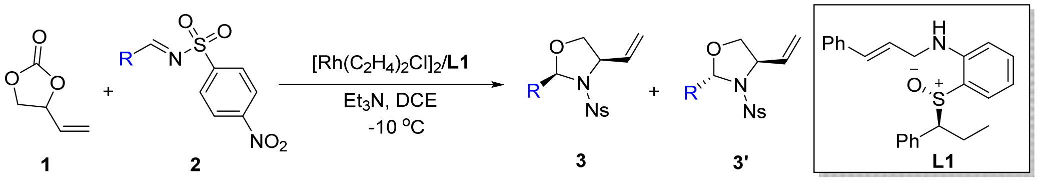

In a screw-cap Schlenk tube filled with argon, $[Rh(C_2H_4)_2Cl]_2$ (0.004 mmol, 2 mol%), Sulfoxide ligand **L1** (0.008 mmol, 4 mol%), N-nosylimine **2** (0.20 mmol) were added. After that, 4-Vinyl-1,3-dioxolan-2-one **1** (0.40 mmol) and 1,2-Dichloroethane (DCE) (2.0 mL) were added. Finally, add Triethylamine (0.3 mmol). After 24 h stirring at -10 °C, the reaction mixture was filtered over silica (CH_2Cl_2) and concentrated under reduced pressure to afford the crude product. The crude residue was purified by flash column chromatography (petroleum ether/ethyl acetate) to give the desired products **3**.

(2*R*,4*R*)-2-(3-chlorophenyl)-3-((4-nitrophenyl)sulfonyl)-4-vinyloxazolidine (3g)

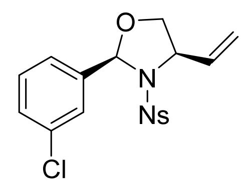

White solid; **m.p.**: 149 - 150 °C; 34% yield (26.7 mg); **HPLC** *ee*: 81% [Daicel CHIRALPAK AD-H (0.46 cm × 25cm); *n*-hexane/2-propanol = 90/10; flow rate = 1.0 mL/min; detection wavelength = 214 nm; t_R =20.36 (major), 23.67 (minor) min]. **[α]$^{20}_{D}$** = + 29.1 (c 1.0, $CHCl_3$). **^{1}H NMR** (400 MHz, $CDCl_3$) δ 8.31 (d, *J* = 8.8 Hz, 2H), 7.87 (d, *J* = 8.9 Hz, 2H), 7.42 – 7.36 (m, 2H), 7.35 – 7.29 (m, 2H), 6.18 (s, 1H), 5.77 – 5.68 (m, 1H), 5.34 (d, *J* = 17.0 Hz, 1H), 5.26 (d, *J* = 10.1 Hz, 1H), 4.45 (q, *J* = 7.1 Hz, 1H), 4.08 (dd, *J* = 9.1, 7.1 Hz, 1H), 3.78 (dd, *J* = 9.1, 5.4 Hz, 1H). **^{13}C NMR** (101 MHz, $CDCl_3$) δ 150.4, 144.3, 139.0, 135.1, 134.7, 130.0, 129.6, 129.0, 127.5, 125.7, 124.3, 119.6, 91.6, 71.2, 62.0. **IR** (CH_2Cl_2): ν_{max} (cm^{-1}) = 3104, 3061, 2960, 2922, 2871, 2299, 1759, 1600, 1532, 1351, 1266, 1174, 1120, 850, 755, 692, 631, 574. **HRMS** (ESI^+) calcd for $C_{17}H_{15}ClN_2NaO_5S$ $[M+Na]^+$: 417.0282, Found: 417.0294.

(2*S*,4*R*)-2-(3-chlorophenyl)-3-((4-nitrophenyl)sulfonyl)-4-vinyloxazolidine (3g')

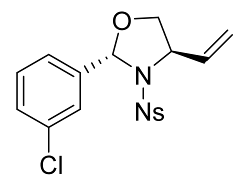

White solid; **m.p.**: 149 - 150 °C; 25% yield (19.7 mg); **HPLC** *ee*: 70% [Daicel CHIRALPAK AD-H (0.46 cm × 25cm); *n*-hexane/2-propanol = 90/10; flow rate = 1.0 mL/min; detection wavelength = 214 nm; t_R =17.26 (major), 25.36 (minor) min]. **[α]$^{20}_{D}$** = - 10.9 (c 1.0, $CHCl_3$). **^{1}H NMR** (400 MHz, $CDCl_3$) δ 8.19 (d, *J* = 8.9 Hz, 2H), 7.58 (d, *J* = 8.9 Hz, 2H), 7.37 – 7.31 (m, 1H), 7.14 – 7.10 (m, 1H), 6.14 (s, 1H), 5.92 (ddd, *J* = 17.0, 10.1, 8.6 Hz, 1H), 5.46 (d, *J* = 17.0 Hz, 1H), 5.35 (d, *J* = 10.1 Hz, 1H), 4.48 (dt, *J* = 8.6, 5.6 Hz, 1H), 4.30 (dd, *J* = 8.8, 6.0 Hz, 1H), 3.88 (dd, *J* = 8.8, 5.3 Hz, 1H). **^{13}C NMR** (101 MHz, $CDCl_3$) δ 149.9, 146.0, 138.7, 134.5, 134.1, 129.8, 129.7, 128.4, 128.0, 126.7, 123.9, 119.6, 91.5, 71.8, 62.7. **IR** (CH_2Cl_2): ν_{max} (cm^{-1}) = 3113, 3067, 2961, 2928, 2873, 2300, 1763, 1607, 1531, 1355, 1268, 1177, 1121, 858, 759, 691, 638, 579. **HRMS** (ESI^+) calcd for $C_{17}H_{15}ClN_2NaO_5S$ $[M+Na]^+$: 417.0282, Found: 417.0294.

(2*R*,4*R*)-2-(3-methoxyphenyl)-3-((4-nitrophenyl)sulfonyl)-4-vinyloxazolidine (3h)

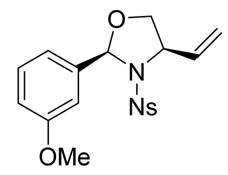

Pale yellow solid; **m.p.**: 108 - 110 °C; 40% yield (31.2 mg); **HPLC** *ee*: 85% [Daicel CHIRALPAK IA-H (0.46 cm × 25cm); *n*-hexane/2-propanol = 90/10; flow rate = 1.0 mL/min; detection wavelength = 254 nm; t_R =23.56 (major), 29.08 (minor) min]. **[α]$^{20}_{D}$** = + 77.2 (c 1.0, $CHCl_3$). **^{1}H NMR** (600 MHz, $CDCl_3$) δ 8.12 (d, *J* = 8.8 Hz, 2H), 7.53 (d, *J* = 8.8 Hz, 2H), 7.19 (t, *J* = 7.9 Hz, 1H), 6.90 (d, *J* = 7.6 Hz, 1H), 6.86 (dd, *J* = 8.2, 1.7 Hz, 1H), 6.68 (t, *J* = 2.1 Hz, 1H), 6.12 (s, 1H), 5.92 (ddd, *J* = 17.0, 10.1, 8.6 Hz, 1H), 5.43 (d, *J* = 17.0 Hz, 1H), 5.31 (d, *J* = 10.1 Hz, 1H), 4.45 (dt, *J* = 8.6, 5.7 Hz, 1H), 4.30 (dd, *J* = 8.8, 6.1 Hz, 1H), 3.86 (dd, *J* = 8.8, 5.4 Hz, 1H), 3.70 (s, 3H). **^{13}C NMR** (101 MHz, $CDCl_3$) δ 159.6, 149.7, 146.1, 138.1, 134.5, 129.4, 128.4, 123.7, 120.7, 119.3, 114.8, 113.9, 92.2, 71.8, 62.6, 55.3. **IR** (CH_2Cl_2): ν_{max} (cm^{-1}) = 3109, 3066, 2972, 2916, 2855, 2271, 1739, 1626, 1531, 1327, 1244, 1176, 871, 786, 698, 643. **HRMS** (ESI^+) calcd for $C_{18}H_{18}N_2NaO_6S$ $[M+Na]^+$: 413.0778, Found: 413.0782.

(2*S*,4*R*)-2-(3-methoxyphenyl)-3-((4-nitrophenyl)sulfonyl)-4-vinyloxazolidine (3h')

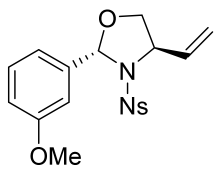

Pale yellow solid; **m.p.**: 108 - 110 °C; 21% yield (16.3 mg); **HPLC** *ee*: 57% [Daicel CHIRALPAK IA-H (0.46 cm × 25cm); n-hexane/2-propanol = 90/10; flow rate = 1.0 mL/min; detection wavelength = 254 nm; t_R =23.43 (major), 26.60 (minor) min]. $[\alpha]^{20}_D$ = - 26.5 (c 1.0, $CHCl_3$). **^{1}H NMR** (600 MHz, $CDCl_3$) δ 8.12 (d, *J* = 8.4 Hz, 2H), 7.53 (d, *J* = 8.7 Hz, 2H), 7.19 (t, *J* = 7.9 Hz, 1H), 6.90 (d, *J* = 7.6 Hz, 1H), 6.88 – 6.83 (m, 1H), 6.68 (t, *J* = 2.0 Hz, 1H), 6.12 (s, 1H), 5.92 (dt, *J* = 17.4, 9.3 Hz, 1H), 5.43 (d, *J* = 17.0 Hz, 1H), 5.32 (d, *J* = 10.1 Hz, 1H), 4.45 (dt, *J* = 8.6, 5.8 Hz, 1H), 4.30 (dd, *J* = 8.8, 6.0 Hz, 1H), 3.86 (dd, *J* = 8.6, 5.2 Hz, 1H), 3.71 (s, 3H). **^{13}C NMR** (151 MHz, $CDCl_3$) δ 159.5, 149.7, 146.0, 138.0, 134.4, 129.4, 128.4, 123.7, 120.7, 119.3, 114.8, 113.8, 92.2, 71.8, 62.6, 55.3. **IR** (CH_2Cl_2): ν_{max} (cm^{-1}) = 3103, 3059, 2962, 2909, 2851, 2274, 1733, 1624, 1537, 1322, 1241, 1175, 866, 781, 671, 649. **HRMS** (ESI^+) calcd for $C_{18}H_{18}N_2NaO_6S$ $[M+Na]^+$: 413.0778, Found: 413.0782.

(2*R*,4*R*)-2-(3-ethoxyphenyl)-3-((4-nitrophenyl)sulfonyl)-4-vinyloxazolidine (3i)

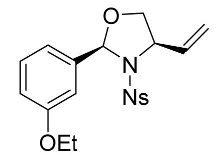

Yellow solid; **m.p.**: 100 - 102 °C; 32% yield (25.8 mg); **HPLC** *ee*: 73% [Daicel CHIRALPAK IC-H (0.46 cm × 25cm); *n*-hexane/2-propanol = 95/5; flow rate = 1.0 mL/min; detection wavelength = 254 nm; t_R =48.18 (major), 51.53 (minor) min]. $[\alpha]^{20}_D$ = + 15.9 (c 1.0, $CHCl_3$). **^{1}H NMR** (600 MHz, $CDCl_3$) δ 8.26 (d, *J* = 8.8 Hz, 2H), 7.82 (d, *J* = 8.8 Hz, 2H), 7.24 (t, *J* = 7.9 Hz, 1H), 7.01 (d, *J* = 7.6 Hz, 1H), 6.91 (t, *J* = 2.1 Hz, 1H), 6.86 (dd, *J* = 8.1, 2.4 Hz, 1H), 6.17 (s, 1H), 5.80 – 5.73 (m, 1H), 5.34 (d, *J* = 17.0 Hz, 1H), 5.24 (d, *J* = 10.2 Hz, 1H), 4.51 – 4.46 (m, 1H), 4.09 (dd, *J* = 9.1, 7.0 Hz, 1H), 4.01 – 3.94 (m, 2H), 3.81 (dd, *J* = 9.1, 5.2 Hz, 1H), 1.39 (t, *J* = 7.0 Hz, 3H). **^{13}C NMR** (151 MHz, $CDCl_3$) δ 159.1, 150.1, 144.6, 138.1, 135.4, 129.7, 128.9, 124.1, 119.6, 119.3, 115.4, 113.5, 92.3, 71.1, 63.6, 62.0, 14.8. **IR** (CH_2Cl_2): ν_{max} (cm^{-1}) = 3058, 2960, 2928, 2869, 2307, 1771, 1603, 1539, 1353, 1268, 1174, 857, 756, 703, 620. **HRMS** (ESI^+) calcd for $C_{19}H_{20}N_2NaO_6S$ $[M+Na]^+$: 427.0934, Found: 427.0928.

(2*S*,4*R*)-2-(3-ethoxyphenyl)-3-((4-nitrophenyl)sulfonyl)-4-vinyloxazolidine (3i′)

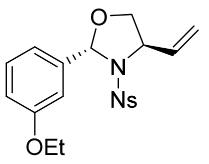

Yellow solid; **m.p.**: 100 - 102 °C; 26% yield (21.0 mg); **HPLC** *ee*: 81% [Daicel CHIRALPAK IC-H (0.46 cm × 25cm); *n*-hexane/2-propanol = 95/5; flow rate = 1.0 mL/min; detection wavelength = 254 nm; t_R =63.56 (minor), 70.99 (major) min]. $[\alpha]^{20}_D$ = - 9.2 (c 1.0, $CHCl_3$). **^{1}H NMR** (400 MHz, $CDCl_3$) δ 8.13 (d, *J* = 8.9 Hz, 2H), 7.53 (d, *J* = 8.8 Hz, 2H), 7.19 (t, *J* = 7.9 Hz, 1H), 6.93 – 6.83 (m, 2H), 6.66 (t, *J* = 2.1 Hz, 1H), 6.12 (s, 1H), 5.96 (ddd, *J* = 17.1, 10.1, 8.5 Hz, 1H), 5.45 (d, *J* = 17.0 Hz, 1H), 5.34 (d, *J* = 10.1 Hz, 1H), 4.47 (dt, *J* = 8.5, 5.7 Hz, 1H), 4.32 (dd, *J* = 8.7, 6.0 Hz, 1H), 3.97 – 3.91 (m, 1H), 3.91 – 3.83 (m, 2H), 1.38 (t, *J* = 7.0 Hz, 3H). **^{13}C NMR** (101 MHz, $CDCl_3$) δ 158.9, 149.7, 146.1, 137.9, 134.5, 129.4, 128.4, 123.7, 120.6, 119.2, 115.3, 114.4, 92.2, 71.8, 63.6, 62.7, 14.8. **IR** (CH_2Cl_2): ν_{max} (cm^{-1}) = 3055, 2962, 2930, 2869, 2307, 1773, 1601, 1537, 1352, 1269, 1177, 860, 755, 701, 621. **HRMS** (ESI^+) calcd for $C_{19}H_{20}N_2NaO_6S$ $[M+Na]^+$: 427.0934, Found: 427.0928.

(2,4*R*)-3-((4-nitrophenyl)sulfonyl)-2-(3-phenoxyphenyl)-4-vinyloxazolidine (3j/3j′)

(*: representative signals for the minor diastereoisomer)

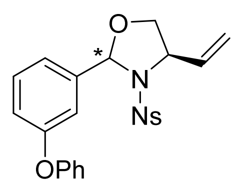

White solid; **m.p.**: 118 - 120 °C; 54% yield (48.7 mg); **HPLC** *ee*: 70% /77% [Daicel CHIRALCEL OD-H (0.46 cm × 25cm); *n*-hexane/2-propanol = 95/5; flow rate = 1.0 mL/min; detection wavelength = 254 nm; t_R =42.89 (major), 56.432 (minor) min, t_R =48.41 (major), 67.63 (minor) min,]. $[\alpha]^{20}_D$ = + 75.8 (c 1.0, $CHCl_3$). **^{1}H NMR** (400 MHz, $CDCl_3$) δ 8.27 (d, *J* = 8.9 Hz, 2H), 8.17* (d, *J* = 8.9 Hz, 1H), 7.84 (d, *J* = 8.8 Hz, 2H), 7.59* (d, *J* = 8.8 Hz, 1H), 7.37 – 7.31 (m, 3H), 7.30 (d, *J* = 7.9 Hz, 1H), 7.25 – 7.19 (m, 2H), 7.15 – 7.10 (m, 2H), 7.08 (dt, *J* = 7.7, 1.3 Hz, 1H), 7.03 (t, *J* = 2.1 Hz, 1H), 6.98 (dd, *J* = 2.5, 1.1 Hz, 1H), 6.97 – 6.92 (m, 4H), 6.86 (t, *J* = 2.0 Hz, 1H), 6.20 (s, 1H), 6.13 * (s, 1H), 5.88* (ddd, *J* = 17.1, 10.1, 8.6 Hz, 1H), 5.68 (ddd, *J* = 17.1, 10.2, 7.8 Hz, 1H), 5.45 – 5.35 * (m, 1H), 5.31 (s, 1H), 5.28* (d, *J* = 6.5 Hz, 1H), 5.19 (dt, *J* = 10.1, 1.0 Hz, 1H), 4.51 – 4.44 (m, 1H), 4.44 – 4.39 * (m, 1H), 4.27* (dd, *J* = 8.8, 6.0 Hz, 1H), 4.07 (dd, *J* = 9.1, 7.0 Hz, 1H), 3.84* (dd, *J* = 8.8, 5.1 Hz, 1H), 3.78 (dd, *J* = 9.1, 5.2 Hz, 1H). **^{13}C NMR** (101 MHz, $CDCl_3$) δ 157.7, 157.4*, 156.7, 156.57* , 150.3, 149.8*, 146.1*, 144.6, 138.9, 138.8*, 135.1, 134.3*, 130.0, 129.9, 129.7, 128.9, 128.5*, 124.2, 123.8, 123.8, 122.9*, 122.3, 119.6*, 119.5, 119.3, 119.2, 119.1*, 118.1*, 117.4, 92.0, 91.9*, 71.7*, 71.1, 62.6*, 61.9. **IR** (CH_2Cl_2): ν_{max} (cm^{-1}) = 3107, 3055, 2985, 2927, 2830, 2281, 1726, 1616, 1522, 1344, 1267, 1182, 878, 789, 690, 621. **HRMS** (ESI^+) calcd for $C_{23}H_{20}N_2NaO_6S$ $[M+Na]^+$: 475.0934, Found: 475.0927.

(2*R*,4*R*)-3-((4-nitrophenyl)sulfonyl)-2-(m-tolyl)-4-vinyloxazolidine (3k)

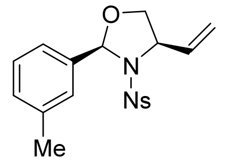

Pale yellow solid; **m.p.**: 105 - 107 °C; 44% yield (32.8 mg); **HPLC** *ee*: 73% [Daicel CHIRALPAK AD-H (0.46 cm × 25cm); *n*-hexane/2-propanol = 90/10; flow rate = 1.0 mL/min; detection wavelength = 254 nm; t_R =15.36 (major), 18.38 (minor) min]. $[\alpha]^{20}_D$ = +31.2 (c 1.0, $CHCl_3$). **^{1}H NMR** (600 MHz, $CDCl_3$) δ 8.24 (d, *J* = 8.4 Hz, 2H), 7.79 (d, *J* = 8.5 Hz, 2H), 7.25 – 7.17 (m, 3H), 7.16 (d, *J* = 6.6 Hz, 1H), 6.16 (s, 1H), 5.82 – 5.74 (m, 1H), 5.35 (d, *J* = 17.1 Hz, 1H), 5.25 (d, *J* = 10.1 Hz, 1H), 4.54 – 4.46 (m, 1H), 4.09 (dd, *J* = 9.1, 7.0 Hz, 1H), 3.82 (dd, *J* = 9.2, 4.9 Hz, 1H), 2.30 (s, 3H). **^{13}C NMR** (101 MHz, $CDCl_3$) δ 150.2, 144.8, 138.3, 136.5, 135.5, 130.2, 128.9, 128.5, 128.1, 124.7, 124.1, 119.2, 92.6, 71.2, 62.0, 21.4. **IR** (CH_2Cl_2): ν_{max} (cm^{-1}) = 3109, 3058, 2981, 2923, 2830, 2289, 1724, 1613, 1516, 1342, 1261, 1177, 880, 781, 686, 621. **HRMS** (ESI^+) calcd for $C_{18}H_{18}N_2NaO_5S$ $[M+Na]^+$: 397.0829, Found: 397.0842.

(2*S*,4*R*)-3-((4-nitrophenyl)sulfonyl)-2-(m-tolyl)-4-vinyloxazolidine (3k′)

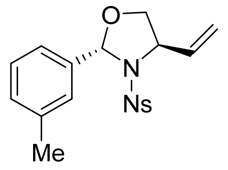

Pale yellow solid; **m.p.**: 105 - 107 °C; 23% yield (17.2 mg); **HPLC** *ee*: 65% [Daicel CHIRALPAK AD-H (0.46 cm × 25cm); *n*-hexane/2-propanol = 90/10; flow rate = 1.0 mL/min; detection wavelength = 214 nm; t_R =14.15 (major), 19.33 (minor) min]. $[\alpha]^{20}_D$ = - 16.1 (c 1.0, $CHCl_3$). **^{1}H NMR** (600 MHz, $CDCl_3$) δ 8.10 (d, *J* = 8.9 Hz, 2H), 7.48 (d, *J* = 8.8 Hz, 2H), 7.15 (d, *J* = 6.8 Hz, 2H), 7.12 – 7.07 (m, 1H), 6.96 (s, 1H), 6.11 (s, 1H), 5.97 – 5.90 (m, 1H), 5.43 (d, *J* = 17.0 Hz, 1H), 5.32 (d, *J* = 10.1 Hz, 1H), 4.45 (dt, *J* = 8.7, 5.8 Hz, 1H), 4.30 (dd, *J* = 8.8, 6.1 Hz, 1H), 3.86 (dd, *J* = 8.8, 5.5 Hz, 1H), 2.21 (s, 3H). **^{13}C NMR** (101 MHz, $CDCl_3$) δ 149.7, 146.1, 138.1, 136.5, 134.5, 130.5, 128.6, 128.5, 128.3, 125.6, 123.6, 119.2, 92.5, 71.8, 62.7, 21.3. **IR** (CH_2Cl_2): ν_{max} (cm^{-1}) = 3109, 3058, 2981, 2923, 2830, 2289, 1724, 1613, 1516,

1342, 1261, 1177, 880, 781, 686, 621. **HRMS** (ESI+) calcd for $C_{18}H_{18}N_2NaO_5S$ [M+Na]+: 397.0829, Found: 397.0842.

(2*R*,4*R*)-2-(3,4-dimethylphenyl)-3-((4-nitrophenyl)sulfonyl)-4-vinyloxazolidine (3l)

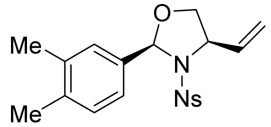

White solid; **m.p.**: 110 - 113 °C; 34% yield (26.3 mg); **HPLC** *ee*: 87% [Daicel CHIRALPAK AD-H (0.46 cm × 25cm); *n*-hexane/2-propanol = 98/2; flow rate = 1.0 mL/min; detection wavelength = 214 nm; t_R =39.19 (major), 54.61 (minor) min]. **[α]$^{20}_D$** = +32.9 (c 1.0, $CHCl_3$). **^{1}H NMR** (600 MHz, $CDCl_3$) δ 8.22 (d, *J* = 8.6 Hz, 2H), 7.78 (d, *J* = 8.7 Hz, 2H), 7.14 (d, *J* = 7.7 Hz, 1H), 7.11 (s, 1H), 7.07 (d, *J* = 7.7 Hz, 1H), 6.12 (s, 1H), 5.79 (ddd, *J* = 17.7, 10.1, 8.1 Hz, 1H), 5.35 (d, *J* = 17.1 Hz, 1H), 5.25 (d, *J* = 10.1 Hz, 1H), 4.49 (q, *J* = 7.3 Hz, 1H), 4.08 (dd, *J* = 8.9, 7.1 Hz, 1H), 3.82 (dd, *J* = 9.1, 4.8 Hz, 1H), 2.25 (s, 3H), 2.19 (s, 3H). **^{13}C NMR** (151 MHz, $CDCl_3$) δ 150.0, 144.8, 138.2, 136.9, 135.5, 133.8, 129.7, 128.9, 128.6, 125.1, 124.0, 119.3, 92.5, 71.1, 61.9, 19.9, 19.7. **IR** (CH_2Cl_2): ν_{max} (cm^{-1}) = 3110, 3059, 2979, 2928, 2830, 2280, 1725, 1613, 1517, 1341, 1269, 1181, 873, 788, 682, 675, 620. **HRMS** (ESI+) calcd for $C_{19}H_{20}N_2NaO_5S$ [M+Na]+: 411.0985, Found: 411.09931.

(2*S*,4*R*)-2-(3,4-dimethylphenyl)-3-((4-nitrophenyl)sulfonyl)-4-vinyloxazolidine (3l′)

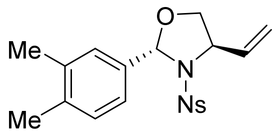

White solid; **m.p.**: 110 - 113 °C; 21% yield (16.2 mg); **HPLC** *ee*: 81% [Daicel CHIRALPAK AD-H (0.46 cm × 25cm); *n*-hexane/2-propanol = 98/2; flow rate = 1.0 mL/min; detection wavelength = 214 nm; t_R =44.27 (minor), 45.44 (major) min]. **[α]$^{20}_D$** = - 88.6 (c 1.0, $CHCl_3$). **^{1}H NMR** (600 MHz, $CDCl_3$) δ 8.07 (d, *J* = 8.7 Hz, 2H), 7.49 (d, *J* = 8.8 Hz, 2H), 7.02 (s, 2H), 6.87 (s, 1H), 6.07 (s, 1H), 5.94 (dt, *J* = 17.5, 9.4 Hz, 1H), 5.43 (d, *J* = 17.0 Hz, 1H), 5.31 (d, *J* = 10.1 Hz, 1H), 4.49 – 4.45 (m, 1H), 4.33 – 4.28 (m, 1H), 3.85 (dd, *J* = 8.6, 5.6 Hz, 1H), 2.24 (s, 3H), 2.08 (s, 3H). **^{13}C NMR** (151 MHz, $CDCl_3$) δ 149.5, 146.1, 138.5, 136.6, 134.6, 133.9, 129.4, 129.0, 128.5, 126.0, 123.5, 119.1, 92.3, 71.7, 62.7, 19.7, 19.7. **IR** (CH_2Cl_2): ν_{max} (cm^{-1}) = 3112, 3058, 2980, 2928, 2831, 2284, 1722, 1617, 1520, 1340, 1266, 1180, 872, 789, 684, 677, 622. **HRMS** (ESI+) calcd for $C_{19}H_{20}N_2NaO_5S$ [M+Na]+: 411.0985, Found: 411.09931.

(2,4*R*)-2-(3,4-dichlorophenyl)-3-((4-nitrophenyl)sulfonyl)-4-vinyloxazolidine (3m/3m′)
(*: representative signals for the minor diastereoisomer)

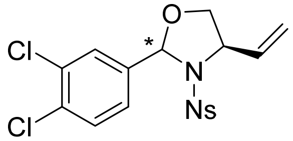

White solid; **m.p.**: 150 - 153 °C; 53% yield (45.3 mg); **HPLC** *ee*: 86%/70% [Daicel CHIRALPAK AD-H (0.46 cm × 25cm); *n*-hexane/2-propanol = 80/20; flow rate = 0.3 mL/min; detection wavelength = 214 nm; t_R =38.50 (major), 48.60 (minor) min, t_R =41.85 (major), 50.46 (minor) min]. **[α]$^{20}_D$** = + 4.8 (c 1.0, $CHCl_3$); **^{1}H NMR** (600 MHz, $CDCl_3$) δ 8.36 (d, *J* = 8.7 Hz, 2H), 8.22* (d, *J* = 8.7 Hz, 1H), 7.94 (d, *J* = 8.7 Hz, 2H), 7.64* (d, *J* = 8.7 Hz, 1H), 7.53 (d, *J* = 2.1 Hz, 1H), 7.45 (d, *J* = 8.3 Hz, 1H), 7.41* (d, *J* = 8.2 Hz, 1H), 7.36 (dd, *J* = 8.4, 2.1 Hz, 1H), 7.29 – 7.24 (m, 2H), 6.17 (s, 1H), 6.10* (s, 1H), 5.85 – 5.78 * (m, 1H), 5.70 (ddd, *J* = 17.4, 10.2, 7.6 Hz, 1H), 5.44 * (d, *J* = 17.0 Hz, 1H), 5.36 – 5.30 (m, 2H), 5.26 (d, *J* = 10.2 Hz, 1H), 4.50 – 4.44* (m, 1H), 4.38 (q, *J* = 6.9 Hz, 1H), 4.25* (dd, *J* = 8.9, 6.1 Hz, 1H), 4.05 (dd, *J* = 9.2, 7.1 Hz, 1H), 3.84* (dd, *J* = 9.0, 5.1 Hz, 1H), 3.73 (dd, *J* = 9.2, 5.8 Hz, 1H). **^{13}C NMR** (151 MHz, $CDCl_3$) δ 150.5, 150.0*, 145.9, 143.7*, 137.4, 137.0*, 134.7, 134.08* , 133.80 , 133.73* ,

133.01 , 132.80*, 130.74 , 130.40* , 129.75* , 129.25 , 129.10* , 128.5, 127.6, 126.7*, 124.5, 124.0*, 120.0, 119.7*, 91.0, 90.8*, 71.6, 71.0*, 62.6, 62.0*. **IR** (CH_2Cl_2): ν_{max} (cm^{-1}) = 3120, 3051, 2977, 2927, 2833, 2273, 1731, 1621, 1516, 1342, 1266, 1180, 876, 792, 690, 626. **HRMS** (ESI^+) calcd for $C_{17}H1_5Cl_2N_2O_5S$ $[M+H]^+$: 429.0073, Found: 429.0081.

(2*R*,4*R*)-2-(3,4-dibromophenyl)-3-((4-nitrophenyl)sulfonyl)-4-vinyloxazolidine (3n)

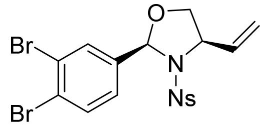

White solid; **m.p.**: 127 - 129 °C; 34% yield (35.0 mg); **HPLC** *ee*: 81% [Daicel CHIRALPAK IG-H (0.46 cm × 25cm); *n*-hexane/2-propanol = 90/10; flow rate = 1.0 mL/min; detection wavelength = 214 nm; t_R =28.22 (major), 34.21 (minor) min]. $[\alpha]^{20}_D$ = + 22.8 (c 1.0, $CHCl_3$). **^{1}H NMR** (400 MHz, $CDCl_3$) δ 8.35 (d, *J* = 8.8 Hz, 2H), 7.97 – 7.88 (m, 2H), 7.68 – 7.59 (m, 2H), 7.31 (dd, *J* = 8.3, 2.1 Hz, 1H), 6.14 (s, 1H), 5.76 – 5.64 (m, 1H), 5.33 (d, *J* = 17.1 Hz, 1H), 5.26 (d, *J* = 10.2 Hz, 1H), 4.40 (q, *J* = 6.8 Hz, 1H), 4.06 (dd, *J* = 9.2, 7.0 Hz, 1H), 3.74 (dd, *J* = 9.2, 5.6 Hz, 1H). **^{13}C NMR** (101 MHz, $CDCl_3$) δ 150.5, 143.9, 138.1, 134.8, 133.9, 132.4, 129.0, 127.6, 126.1, 125.2, 124.5, 119.7, 91.0, 71.1, 62.0. **IR** (CH_2Cl_2): ν_{max} (cm^{-1}) = 3125, 3063, 2981, 2933, 2828, 2271, 1737, 1629, 1513, 1345, 1275, 1181, 870, 793, 688, 616. **HRMS** (ESI^+) calcd for $C_{17}H_{15}Br_2N_2O_5S$ $[M+H]^+$: 516.9063, Found: 516.90651.

(2*R*,4*R*)-2-(3,4-dibromophenyl)-3-((4-nitrophenyl)sulfonyl)-4-vinyloxazolidine (3n′)

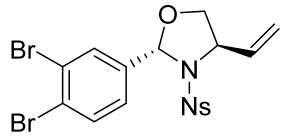

White solid; **m.p.**: 127 - 129 °C; 16% yield (16.4 mg); **HPLC** *ee*: 73% [Daicel CHIRALPAK IG-H (0.46 cm × 25cm); *n*-hexane/2-propanol = 90/10; flow rate = 1.0 mL/min; detection wavelength = 214 nm; t_R =31.97 (major), 39.71 (minor) min]. $[\alpha]^{20}_D$ = - 17.9 (c 1.0, $CHCl_3$). **^{1}H NMR** (400 MHz, $CDCl_3$) δ 8.24 (d, *J* = 8.8 Hz, 2H), 7.68 – 7.55 (m, 3H), 7.40 (d, *J* = 2.1 Hz, 1H), 7.23 (dd, *J* = 8.2, 2.1 Hz, 1H), 6.09 (s, 1H), 5.92 – 5.82 (m, 1H), 5.46 (d, *J* = 17.0 Hz, 1H), 5.34 (d, *J* = 10.1 Hz, 1H), 4.55 – 4.45 (m, 1H), 4.28 (dd, *J* = 8.9, 6.0 Hz, 1H), 3.87 (dd, *J* = 8.9, 5.2 Hz, 1H). **^{13}C NMR** (101 MHz, $CDCl_3$) δ 150.0, 145.9, 137.6, 133.9, 133.6, 132.9, 128.5, 128.4, 126.4, 125.0, 124.0, 119.9, 90.8, 71.7, 62.7. **IR** (CH_2Cl_2): ν_{max} (cm^{-1}) = 3127, 3066, 2988, 2931, 2822, 2284, 1732, 1630, 1515, 1349, 1277, 1187, 875, 799, 681, 620. **HRMS** (ESI^+) calcd for $C_{17}H_{15}Br_2N_2O_5S$ $[M+Na]^+$: 516.9063, Found: 516.90651.

(2,4*R*)-3-((4-nitrophenyl)sulfonyl)-2-(3,4,5-trichlorophenyl)-4-vinyloxazolidine (3o/3o′)
(*: representative signals for the minor diastereoisomer)

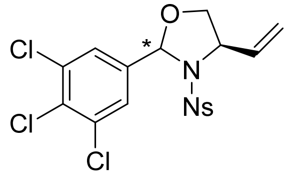

Pale yellow solid; **m.p.**: 133 - 135 °C; 51% yield (46.9 mg); **HPLC** *ee*: 85%/ 53% [Daicel CHIRALPAK IG-H (0.46 cm × 25cm); *n*-hexane/2-propanol = 90/10; flow rate = 1.0 mL/min; detection wavelength = 254 nm; t_R =19.88 (major), 24.49 (minor) min, t_R =16.50 (minor), 20.95 (major) min]. $[\alpha]^{20}_D$ = +10.2 (c 1.0, $CHCl_3$). **^{1}H NMR** (400 MHz, $CDCl_3$) δ 8.38 (d, J = 8.8 Hz, 2H), 8.25* (d, J = 8.8 Hz, 2H), 7.98 (d, J = 8.8 Hz, 2H), 7.71* (d, J = 8.5 Hz, 2H), 7.50 (s, 2H), 7.32* (s, 2H), 6.14 (s, 1H), 6.07* (s, 1H), 5.84 – 5.74 (m, 1H), 5.74 – 5.63* (m, 1H), 5.44 (d, J = 17.1 Hz, 1H), 5.34 (d, J = 11.1 Hz, 1H), 5.32 – 5.25* (m, 2H), 4.54 – 4.46 (m, 1H), 4.36* (q, J = 7.3 Hz, 1H), 4.24 (dd, J = 9.0, 6.0 Hz, 1H), 4.04* (dd, J = 9.2, 7.1 Hz, 1H), 3.84 (dd, J = 9.0, 4.9 Hz, 1H), 3.71* (dd, J = 9.2, 6.0 Hz, 1H). **^{13}C NMR** (151 MHz, $CDCl_3$) δ 150.6, 150.1*, 145.9, 143.4*, 137.8, 137.3*, 134.7, 134.5*,

134.4, 133.5*, 132.8, 132.5*, 129.1, 128.6*, 128.0, 127.4*, 124.6, 124.0*, 120.3, 119.9*, 90.5, 90.3*, 71.7, 71.1*, 62.7, 62.0*. **IR** (CH_2Cl_2): ν_{max} (cm^{-1}) = 3125, 3063, 2981, 2933, 2828, 2271, 1737, 1629, 1513, 1345, 1275, 1181, 870, 793, 688, 616. **HRMS** (ESI^+) calcd for $C_{17}H_{14}Cl_3N_2O_5S$ $[M+H]^+$: 462.9684, Found: 462.9671.

(2,4*R*)-2-(benzofuran-2-yl)-3-((4-nitrophenyl)sulfonyl)-4-vinyloxazolidine (3p/3p′)

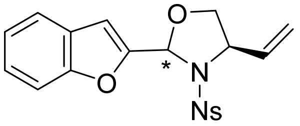

(*: representative signals for the minor diastereoisomer) White solid; **m.p.**: 134 - 137 °C; 68% yield (54.3 mg); **HPLC** *ee*: 11%/91% [Daicel CHIRALPAK AD-H (0.46 cm × 25cm); *n*-hexane/2-propanol = 90/10; flow rate = 1.0 mL/min; detection wavelength = 254 nm; t_R =22.88 (minor), 25.91 (major) min, t_R =29.92 (major), 35.63 (minor) min]. $[\alpha]^{20}_D$ = +42.46 (c 1.0, $CHCl_3$). **^{1}H NMR** (400 MHz, $CDCl_3$) δ 8.14 (d, *J* = 8.9 Hz, 2H), 7.85 (d, *J* = 8.9 Hz, 2H), 7.79 (d, *J* = 8.9 Hz, 2H), 7.56 (dt, *J* = 7.5, 1.7 Hz, 2H), 7.53 (d, *J* = 8.9 Hz, 2H), 7.35 – 7.30 (m, 1H), 7.30 – 7.27 (m, 1H), 7.25 – 7.16 (m, 2H), 6.96* (d, *J* = 8.2 Hz, 1H), 6.90 – 6.84 (m, 2H), 6.38 (s, 1H), 6.27 * (s, 1H), 6.11 – 6.01* (m, 1H), 5.90 (ddd, *J* = 17.1, 10.1, 8.3 Hz, 1H), 5.49* (d, *J* = 17.1 Hz, 1H), 5.46 – 5.34 (m, 2H), 5.31 (dd, *J* = 10.1, 0.9 Hz, 1H), 4.60 – 4.50 (m, 3H), 4.29 – 4.21 (m, 1H), 4.03 – 3.97 (m, 2H). **^{13}C NMR** (151 MHz, $CDCl_3$) δ 155.3, 154.5*, 151.7, 151.7*, 150.1, 149.5*, 144.5, 144.2*, 135.4, 134.6*, 128.9*, 128.2, 127.1*, 126.6, 125.8*, 125.6, 124.0*, 123.7, 123.5*, 123.4, 122.0, 121.8*, 120.2, 118.7*, 111.5, 111.1*, 108.8, 107.9*, 86.1, 85.2*, 73.1, 71.9*, 62.1, 61.8*. **IR** (CH_2Cl_2): ν_{max} (cm^{-1}) = 3123, 3058, 2980, 2921, 2829, 2279, 1731, 1610, 1520, 1344, 1266, 1181, 876, 798, 690, 622. **HRMS** (ESI^+) calcd for $C_{19}H_{16}N_2NaO_6S$ $[M+Na]^+$: 423.0621, Found: 423.0614.

5. General procedure for the synthesis of 4 and 5

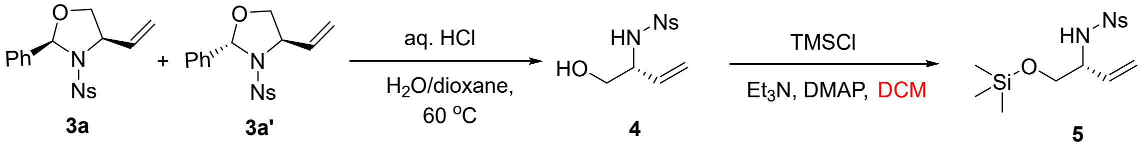

To a solution of (2, 4*R*)-3-((4-nitrophenyl)sulfonyl)-2-phenyl-4-vinyloxazolidine (**3a**/**3a′**) (36 mg, 0.1 mmol) in 1,4-dioxane/water (2:1, 1.5 mL) was added hydrochloric acid (20 μL of 6 N solution, 0.1 mmol) at room temperature. The reaction mixture was stirred at 60 ºC for 12 h. The mixture was then cooled, neutralized with aqueous sodium bicarbonate and extracted with ethyl acetate, dried over anhydrous Na_2SO_4, filtered and concentrated. The residue was purified by flash chromatography (eluent: petroleum ether / ethyl acetate = 2/1) on silica to afford the product **4** (22 mg, 81%) as a white solid.

A round bottom flask equipped with a magnetic stir bar was charged with (*R*)-N-(1-hydroxybut-3-en-2-yl)-4-nitrobenzenesulfonamide **4** (27.2 mg, 0.1 mmol), and then CH_2Cl_2 (2 mL) was added via a glass syringe and followed by the addition of Et_3N (28 μL, 0.2 mmol), DMAP (5.0 mg) and chlorotrimethylsilane (14 μL, 0.11 mmol). The resulting reaction mixture was stirred for 12 h at room temperature. Then quenched with water (10.0 mL) and extracted with CH_2Cl_2 (20 mL). The combined organic layers were washed with

brine and dried over anhydrous Na_2SO_4, filtered and concentrated. The crude product was purified by flash column chromatography (eluent: petroleum ether / ethyl acetate = 5/1) as eluent to afford the pure product **5** in 76% yield as a yellow oil.

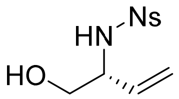

(*R*)-N-(1-hydroxybut-3-en-2-yl)-4-nitrobenzenesulfonamide (4)
White solid; **m.p.**: 102 - 104 °C; 81% yield (22.0 mg); **HPLC** *ee*: 95% [Daicel CHIRALCEL OJ-H (0.46 cm × 25 cm); *n*-hexane/2-propanol = 90/10; flow rate = 1.0 mL/min; detection wavelength = 254 nm; t_R =60.15 (minor), 62.01 (major) min]. **[α]$^{20}_{D}$** = + 11.89 (c 1.0, $CHCl_3$). **^{1}H NMR** (400 MHz, CD_3CN) δ 8.32 (d, *J* = 8.9 Hz, 2H), 8.03 (d, *J* = 8.9 Hz, 2H), 6.06 (d, *J* = 8.0 Hz, 1H), 5.61 (ddd, *J* = 17.1, 10.5, 6.5 Hz, 1H), 5.10 – 4.94 (m, 2H), 3.91 – 3.80 (m, 1H), 3.46 – 3.35 (m, *J* = 5.7 Hz, 2H), 2.91 (t, *J* = 5.9 Hz, 1H) **^{13}C NMR** (151 MHz, CD_3CN) δ 151.0, 148.1, 136.0, 129.2, 125.1, 117.7, 65.0, 59.4. **IR** (CH_2Cl_2): ν_{max} (cm^{-1}) = 3441, 3203, 3018, 2780, 2621, 2229, , 1730, 1601, 1511, 1321, 1209, 1129, 870, 766. **HRMS** (ESI^+) calcd for $C_{10}H_{12}N_2NaO_5S$ $[M+Na]^+$: 295.0359, Found: 295.03551.

(*R*)-4-nitro-N-(1-((trimethylsilyl)oxy)but-3-en-2-yl)benzenesulfonamide (5)

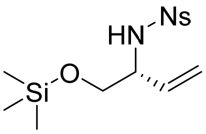

Yellow oil; 76% yield (26.0 mg); **HPLC** *ee*: 95% [Daicel CHIRALPAK AD-H (0.46 cm × 25cm); *n*-hexane/2-propanol = 90/10; flow rate = 1.0 mL/min; detection wavelength = 254 nm; t_R =8.23 (minor), 9.81 (major) min]. **[α]$^{20}_{D}$** = + 12.51 (c 1.0, $CHCl_3$). **^{1}H NMR** (400 MHz, $CDCl_3$) δ 8.33 (d, *J* = 8.7 Hz, 2H), 8.05 (d, *J* = 8.7 Hz, 2H), 5.60 (ddd, *J* = 17.1, 10.1, 6.8 Hz, 1H), 5.19 – 5.06 (m, 2H), 3.91 – 3.85 (m, 1H), 3.59 (dd, *J* = 10.2, 4.3 Hz, 1H), 3.49 (dd, *J* = 10.1, 5.6 Hz, 1H), 0.06 (s, 9H). **^{13}C NMR** (151 MHz, $CDCl_3$) δ 150.1, 146.9, 134.7, 128.6, 124.2, 118.2, 64.8, 58.0. **IR** (CH_2Cl_2): ν_{max} (cm^{-1}) = 3113, 3057, 2983, 2920, 2810, 2226, 1730, 1609, 1511, 1320, 1268, 1181, 876, 790, 687. **HRMS** (ESI^+) calcd for $C_{13}H_{21}N_2O_5SSi$ $[M+H]^+$: 345.0935, Found: 345.0939.

6. X-ray crystallographic information of 3b

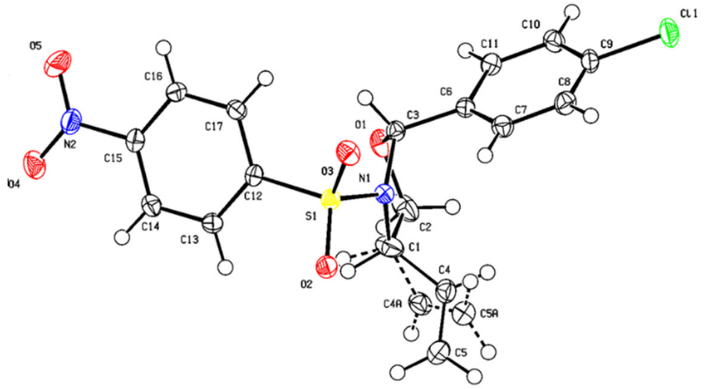

Table S1. Crystal data and structure refinement for compound 3b. (CCDC: 2265381)

CCDC 2265381 (**3b**) contains the supplementary crystallographic data for this paper. These data can be obtained free of charge from The Cambridge Crystallographic Data Centre via www.ccdc.cam.ac.uk/data_request/cif.

Empirical formula	$C_{17}H_{15}ClN_2O_5S$
Formula weight	394.82
Temperature/K	100.00
Crystal system	orthorhombic
Space group	$P2_12_12_1$
a/Å	7.3445(4)
b/Å	10.0239(4)
c/Å	23.4723(12)
α/°	90
β/°	90
γ/°	90
Volume/Å^3	1728.04(15)
Z	4
ρ_{calc}g/cm^3	1.518
μ/mm^{-1}	0.374
F(000)	816.0
Crystal size/mm^3	0.26 × 0.17 × 0.05
Radiation	MoKα (λ = 0.71073)
2Θ range for data collection/°	5.344 to 55.046
Index ranges	$-9 \le h \le 9$, $-12 \le k \le 13$, $-30 \le l \le 30$
Reflections collected	34022
Independent reflections	3960 [R_{int} = 0.0631, R_{sigma} = 0.0330]
Data/restraints/parameters	3960/57/254
Goodness-of-fit on F^2	1.063
Final R indexes [I>=2σ (I)]	R_1 = 0.0315, wR_2 = 0.0681
Final R indexes [all data]	R_1 = 0.0368, wR_2 = 0.0711
Largest diff. peak/hole / e Å^{-3}	0.31/-0.32
Flack parameter	-0.01(3)

7. Copies of NMR spectra of compounds L1-L6, 3 / 3′, 4 and 5

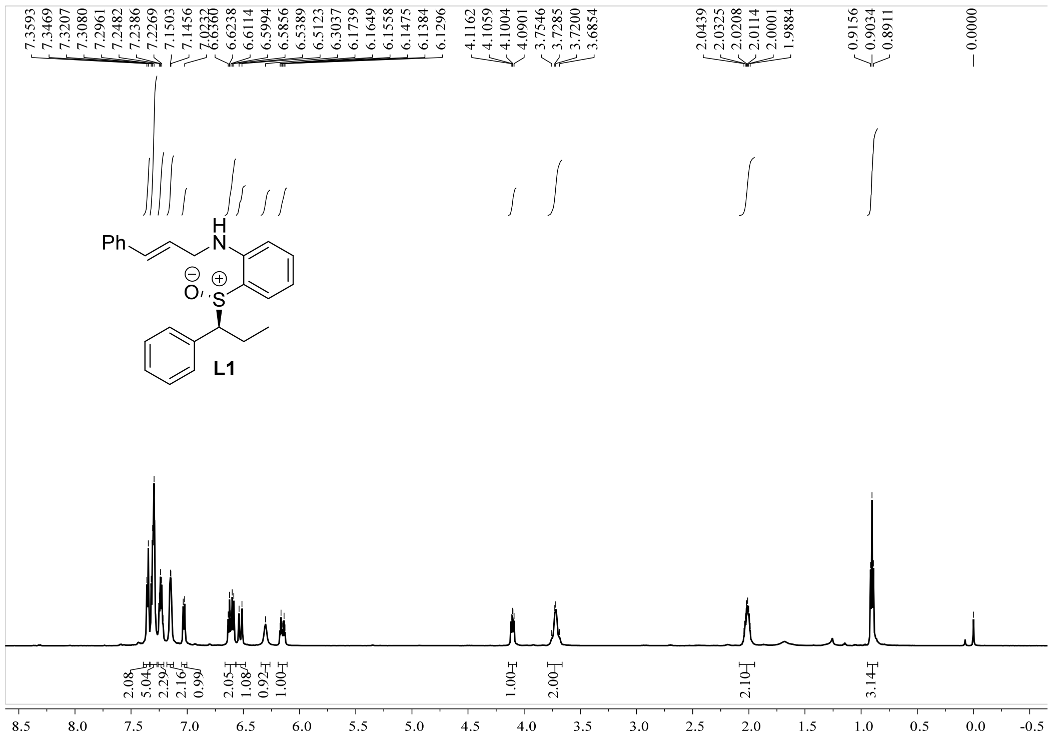

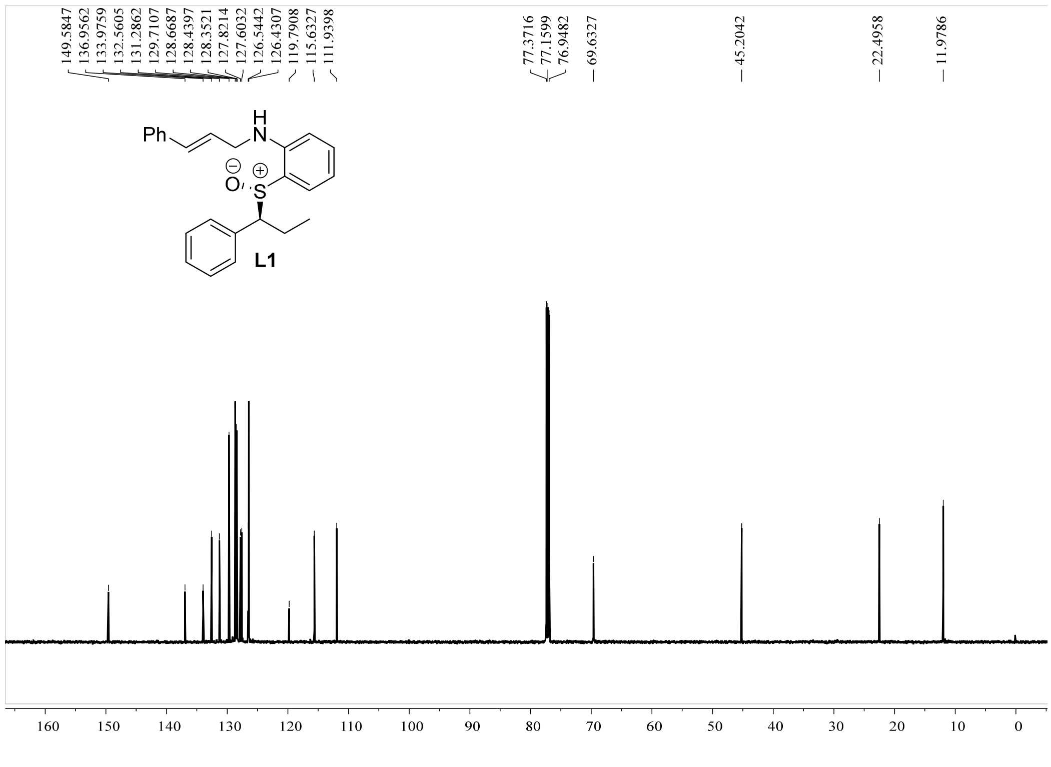

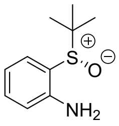

L2

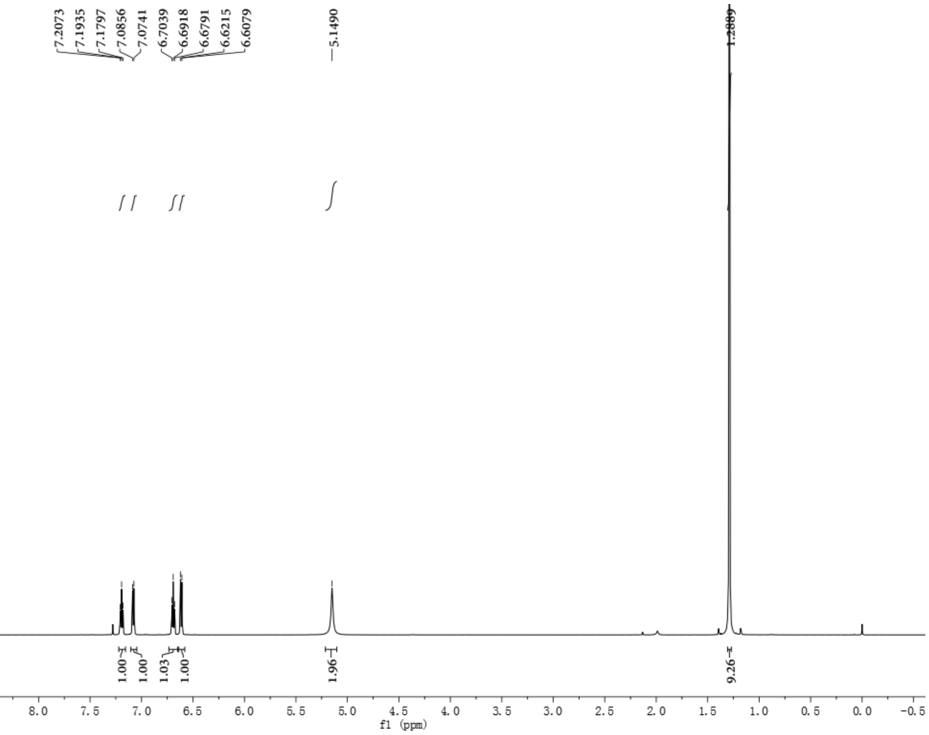
7.2073
7.1935
7.1797
7.0856
7.0741
6.7039
6.6918
6.6791
6.6215
6.6079
5.1490
1.2889
1.00
1.00
1.03
1.00
1.96
9.26
f1 (ppm)

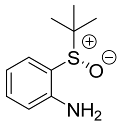

L2

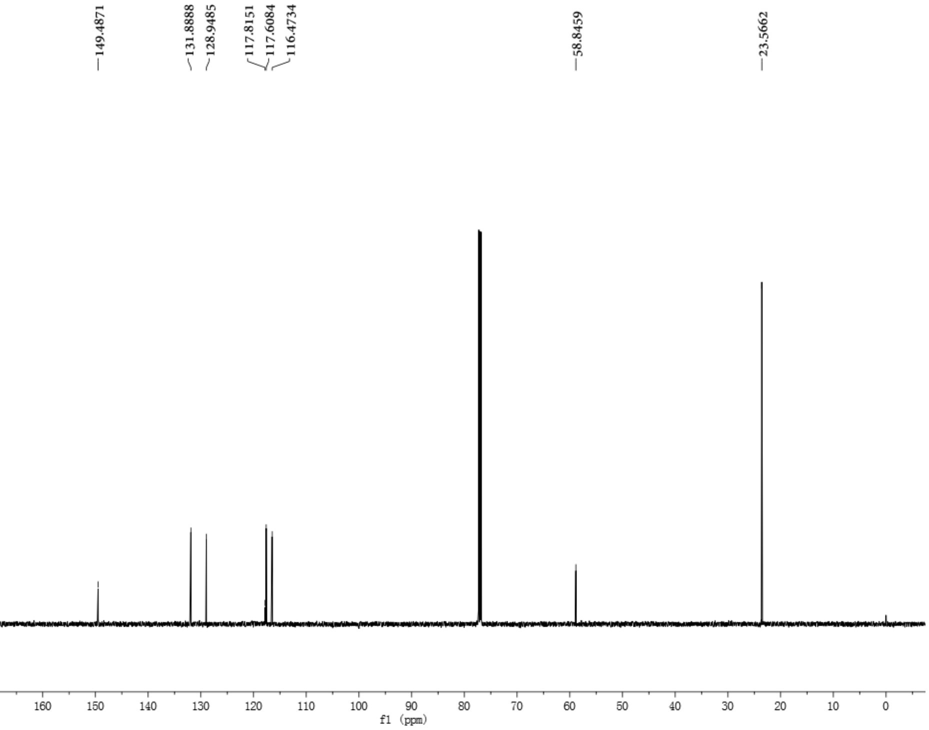
149.4871
131.8888
128.9485
117.8151
117.6084
116.4734
58.8459
23.5662
f1 (ppm)

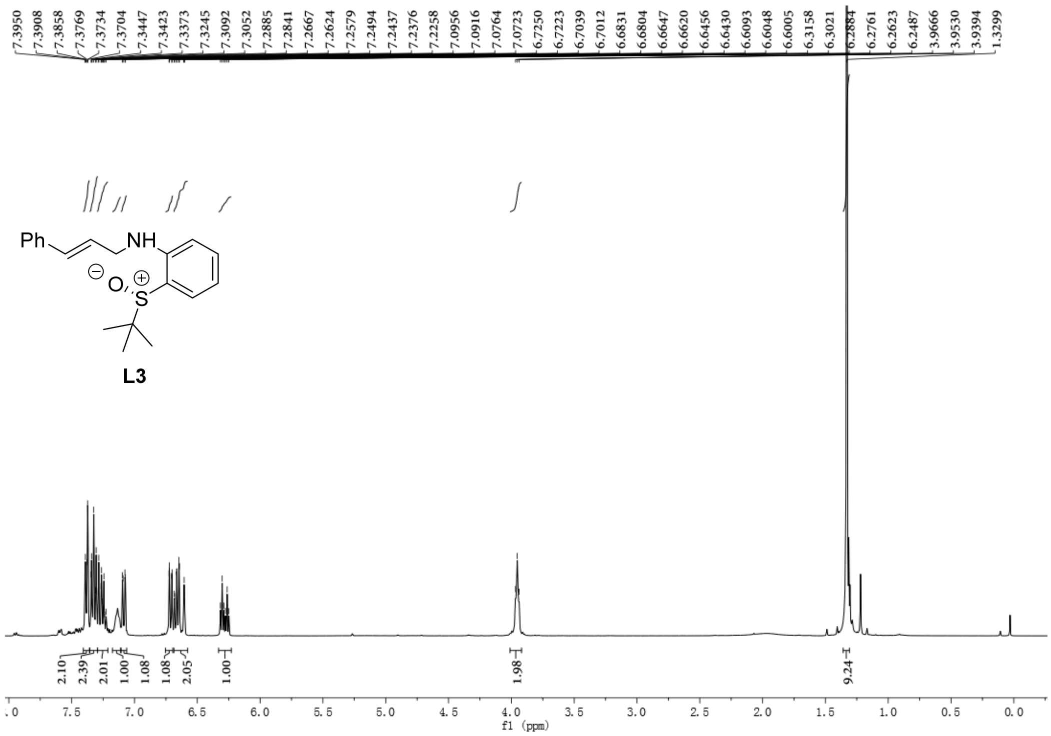
7.3950
7.3908
7.3858
7.3769
7.3734
7.3704
7.3447
7.3423
7.3373
7.3245
7.3092
7.3052
7.2885
7.2841
7.2667
7.2624
7.2579
7.2494
7.2437
7.2376
7.2258
7.0956
7.0916
7.0764
7.0723
6.7250
6.7223
6.7039
6.7012
6.6831
6.6804
6.6647
6.6620
6.6456
6.6430
6.6093
6.6048
6.6005
6.3158
6.3021
6.2884
6.2761
6.2623
6.2487
3.9666
3.9530
3.9394
1.3299
Ph
NH
O
S
L3
2.10
2.39
2.01
1.00
1.08
1.08
2.05
1.00
1.98
9.24
f1 (ppm)

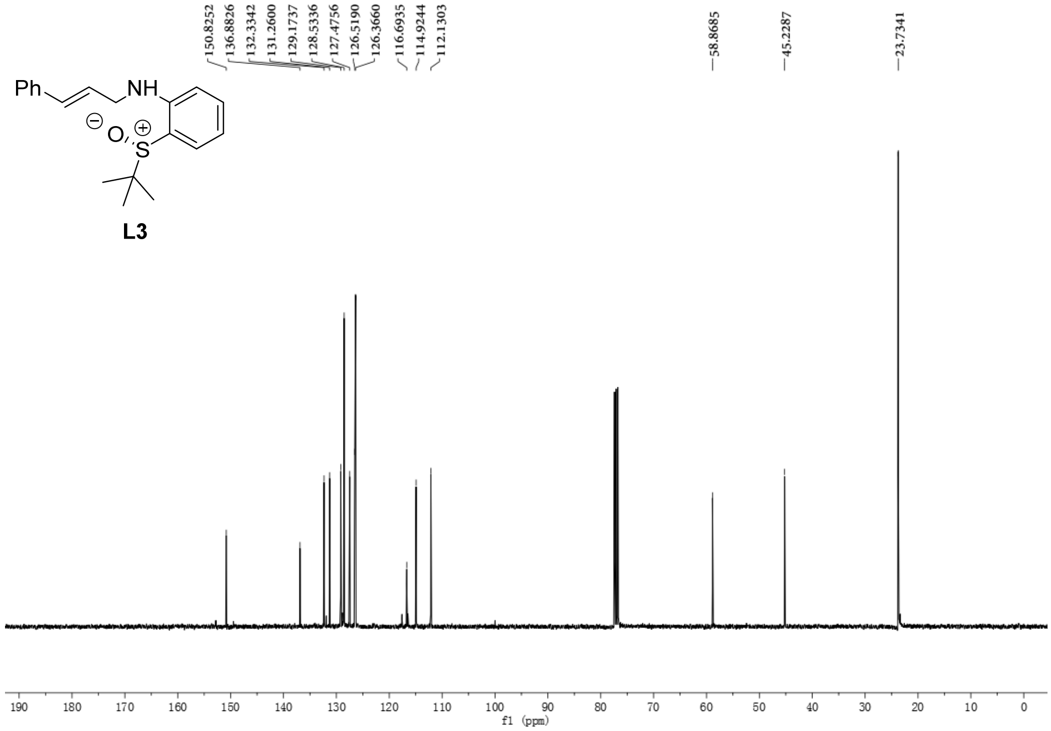
150.8252
136.8826
132.3342
131.2600
129.1737
128.5336
127.4756
126.5190
126.3660
116.6935
114.9244
112.1303
58.8685
45.2287
23.7341
Ph
NH
O
S
L3
f1 (ppm)

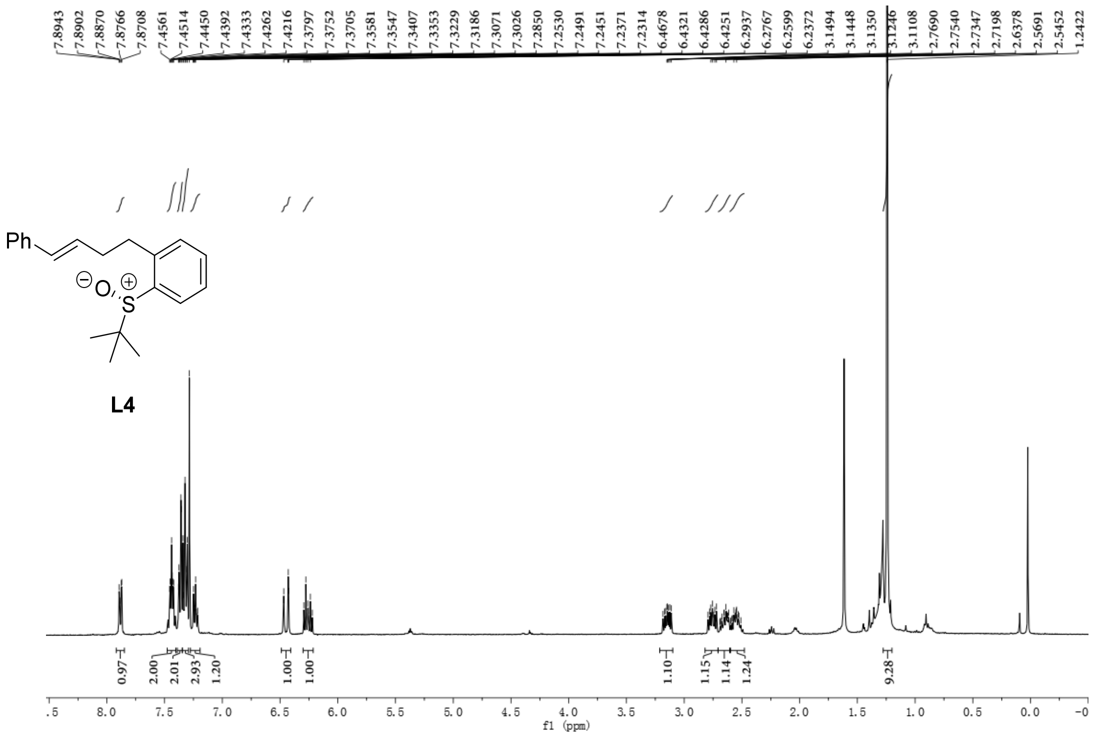
Ph
L4
f1 (ppm)

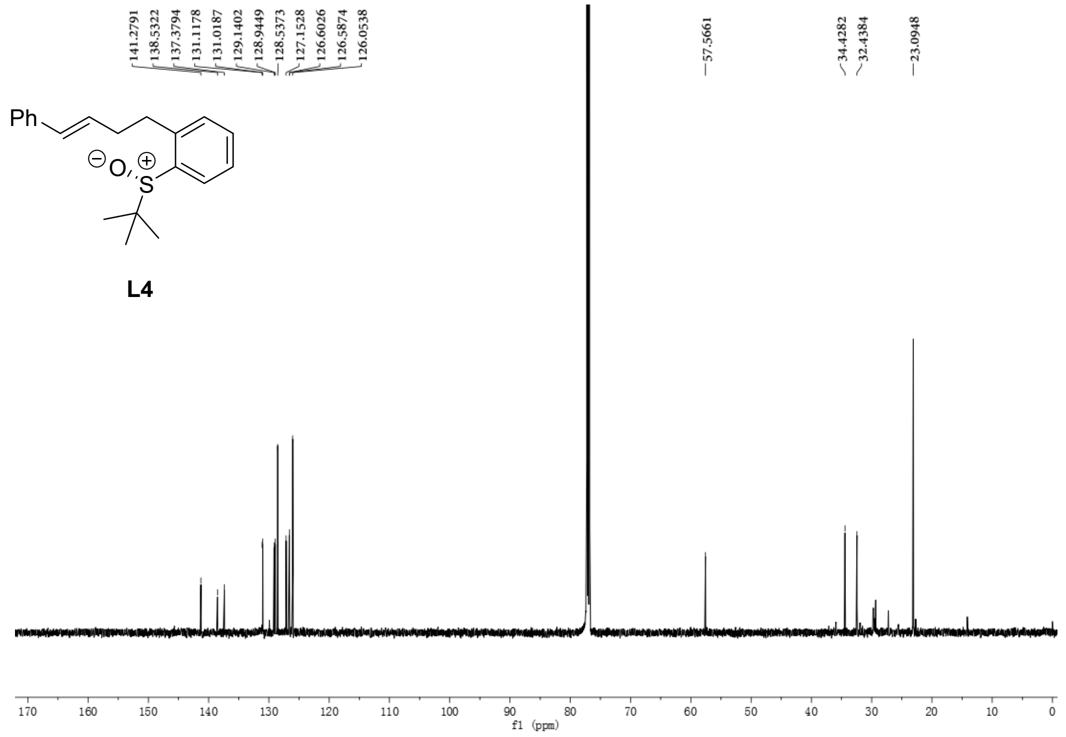
Ph
L4
f1 (ppm)

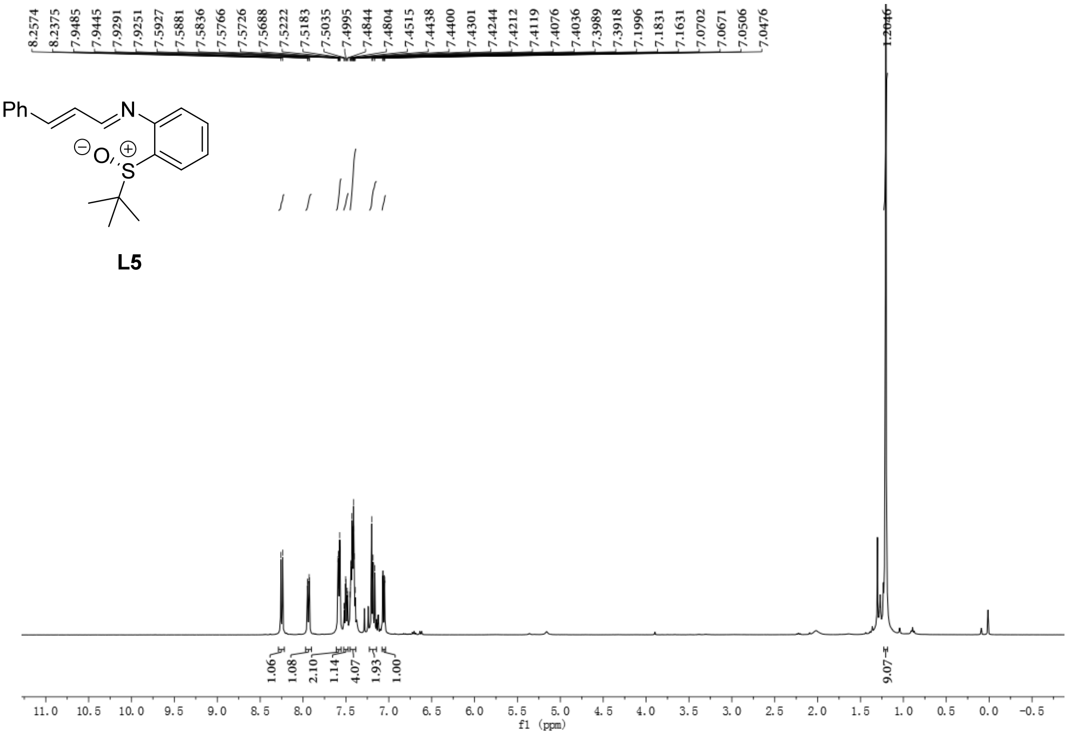
8.2574
8.2375
7.9485
7.9445
7.9291
7.9251
7.5927
7.5881
7.5836
7.5766
7.5726
7.5688
7.5222
7.5183
7.5035
7.4995
7.4844
7.4804
7.4515
7.4438
7.4400
7.4301
7.4244
7.4212
7.4119
7.4076
7.4036
7.3989
7.3918
7.1996
7.1831
7.1631
7.0702
7.0671
7.0506
7.0476
1.2046
Ph
N
O
S
L5
1.06
1.08
2.10
1.14
4.07
1.93
1.00
9.07
f1 (ppm)

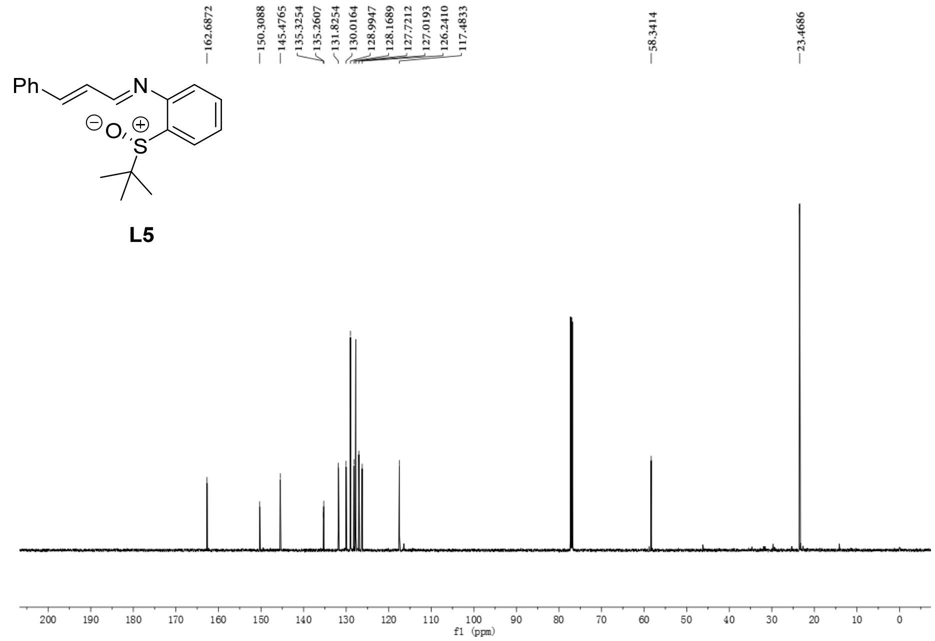
162.6872
150.3088
145.4765
135.3254
135.2607
131.8254
130.0164
128.9947
128.1689
127.7212
127.0193
126.2410
117.4833
58.3414
23.4686
Ph
N
O
S
L5
f1 (ppm)

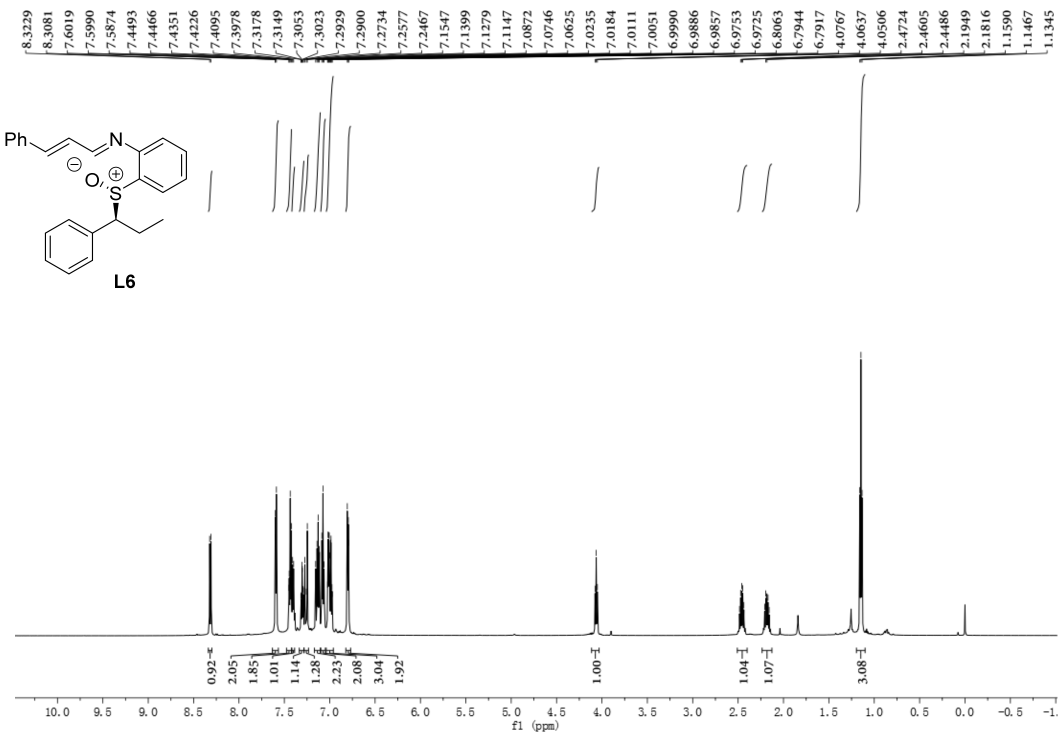
8.3229 8.3081 7.6019 7.5990 7.5874 7.4493 7.4466 7.4351 7.4226 7.4095 7.3978 7.3178 7.3149 7.3053 7.3023 7.2929 7.2900 7.2734 7.2577 7.2467 7.1547 7.1399 7.1279 7.1147 7.0872 7.0746 7.0625 7.0235 7.0184 7.0111 7.0051 6.9990 6.9886 6.9857 6.9753 6.9725 6.8063 6.7944 6.7917 4.0767 4.0637 4.0506 2.4724 2.4605 2.4486 2.1949 2.1816 1.1590 1.1467 1.1345
Ph
N
O
S
L6
0.92 2.05 1.85 1.01 1.14 1.28 2.23 2.08 3.04 1.92 1.00 1.04 1.07 3.08
10.0 9.5 9.0 8.5 8.0 7.5 7.0 6.5 6.0 5.5 5.0 4.5 4.0 3.5 3.0 2.5 2.0 1.5 1.0 0.5 0.0 -0.5
f1 (ppm)

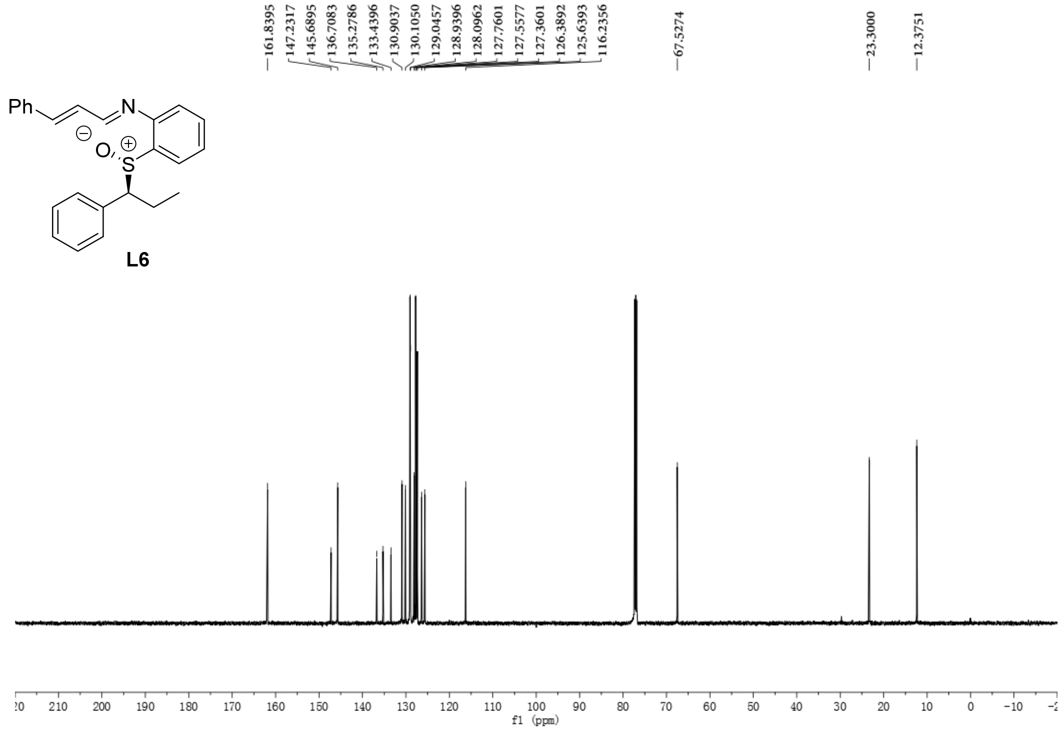
161.8395 147.2317 145.6895 136.7083 135.2786 133.4396 130.9037 130.1050 129.0457 128.9396 128.0962 127.7601 127.5577 127.3601 126.3892 125.6393 116.2356 67.5274 23.3000 12.3751
Ph
N
O
S
L6
210 200 190 180 170 160 150 140 130 120 110 100 90 80 70 60 50 40 30 20 10 0 -10
f1 (ppm)

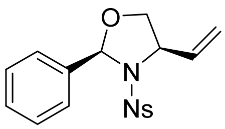
O
N
Ns

3a

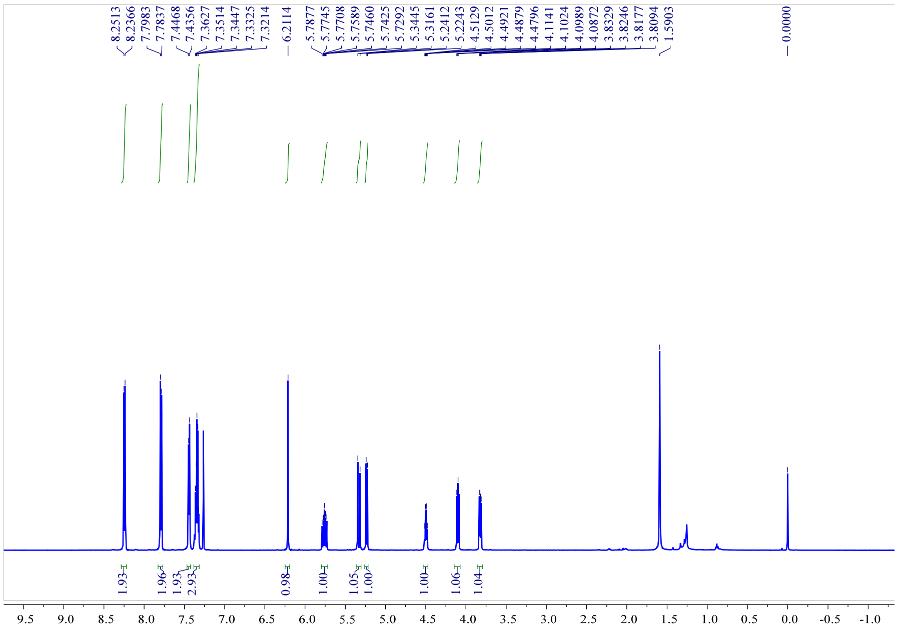
8.2513
8.2366
7.7983
7.7837
7.4468
7.4356
7.3627
7.3514
7.3447
7.3325
7.3214
6.2114
5.7877
5.7745
5.7708
5.7589
5.7460
5.7425
5.7292
5.3445
5.3161
5.2412
5.2243
4.5129
4.5012
4.4921
4.4879
4.4796
4.1141
4.1024
4.0989
4.0872
3.8329
3.8246
3.8177
3.8094
1.5903
0.0000
1.93
1.96
1.93
2.93
0.98
1.00
1.05
1.00
1.00
1.06
1.04
9.5 9.0 8.5 8.0 7.5 7.0 6.5 6.0 5.5 5.0 4.5 4.0 3.5 3.0 2.5 2.0 1.5 1.0 0.5 0.0 -0.5 -1.0

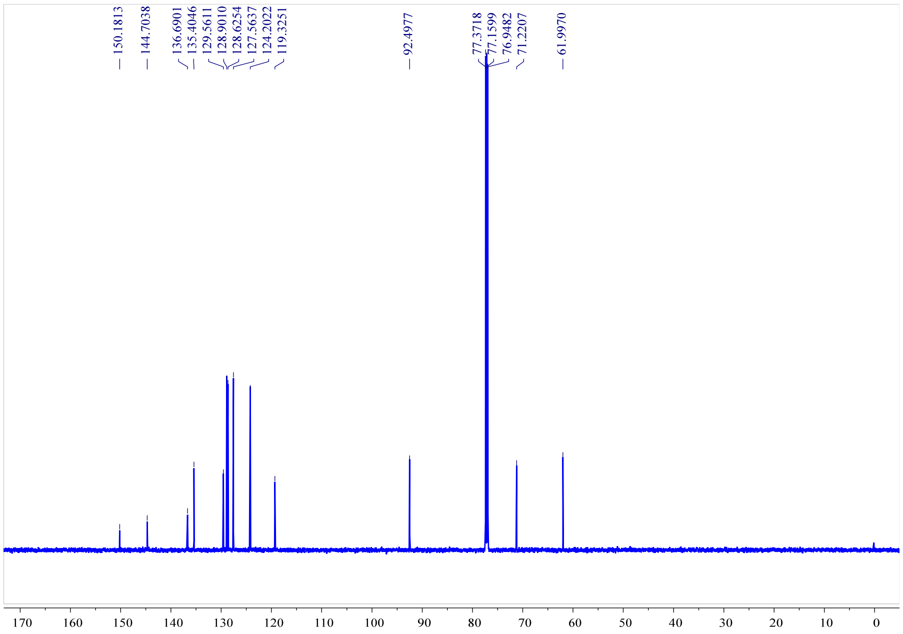
150.1813
144.7038
136.6901
135.4046
129.5611
128.9010
128.6254
127.5637
124.2022
119.3251
92.4977
77.3718
77.1599
76.9482
71.2207
61.9970
170 160 150 140 130 120 110 100 90 80 70 60 50 40 30 20 10 0

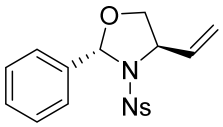
O
N
Ns

3a'

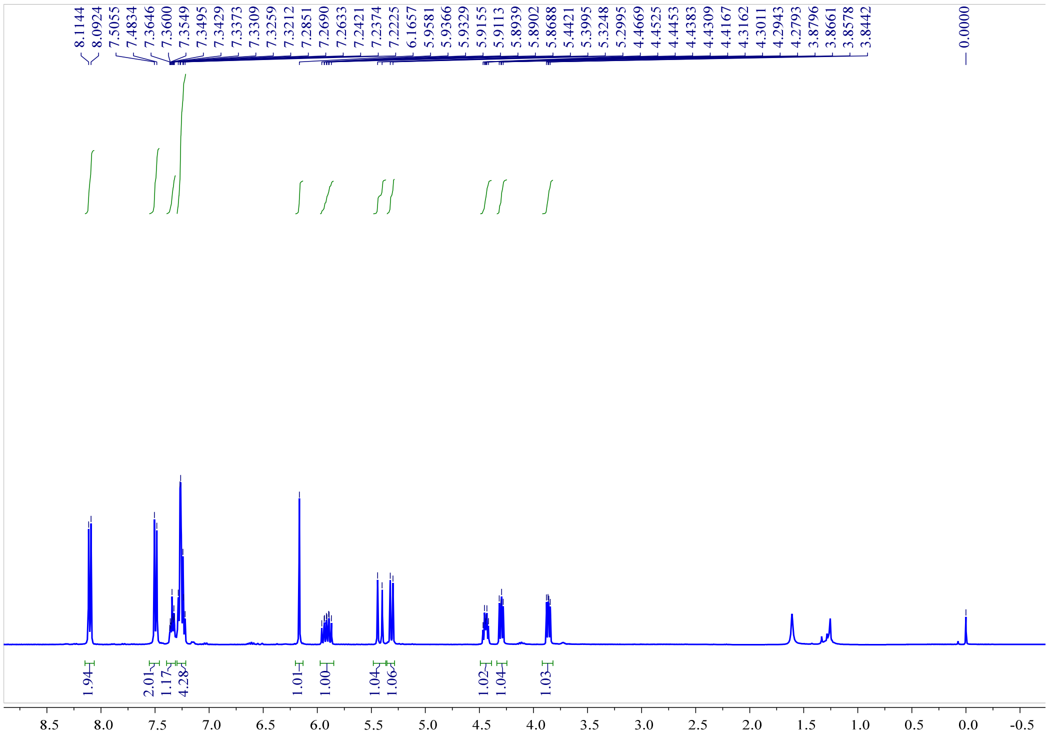
8.1144
8.0924
7.5055
7.4834
7.3646
7.3600
7.3549
7.3495
7.3429
7.3373
7.3309
7.3259
7.3212
7.2851
7.2690
7.2633
7.2421
7.2374
7.2225
6.1657
5.9581
5.9366
5.9329
5.9155
5.9113
5.8939
5.8902
5.8688
5.4421
5.3995
5.3248
5.2995
4.4669
4.4525
4.4453
4.4383
4.4309
4.4167
4.3162
4.3011
4.2943
4.2793
3.8796
3.8661
3.8578
3.8442
0.0000
1.94
2.01
1.17
4.28
1.01
1.00
1.04
1.06
1.02
1.04
1.03
8.5 8.0 7.5 7.0 6.5 6.0 5.5 5.0 4.5 4.0 3.5 3.0 2.5 2.0 1.5 1.0 0.5 0.0 -0.5

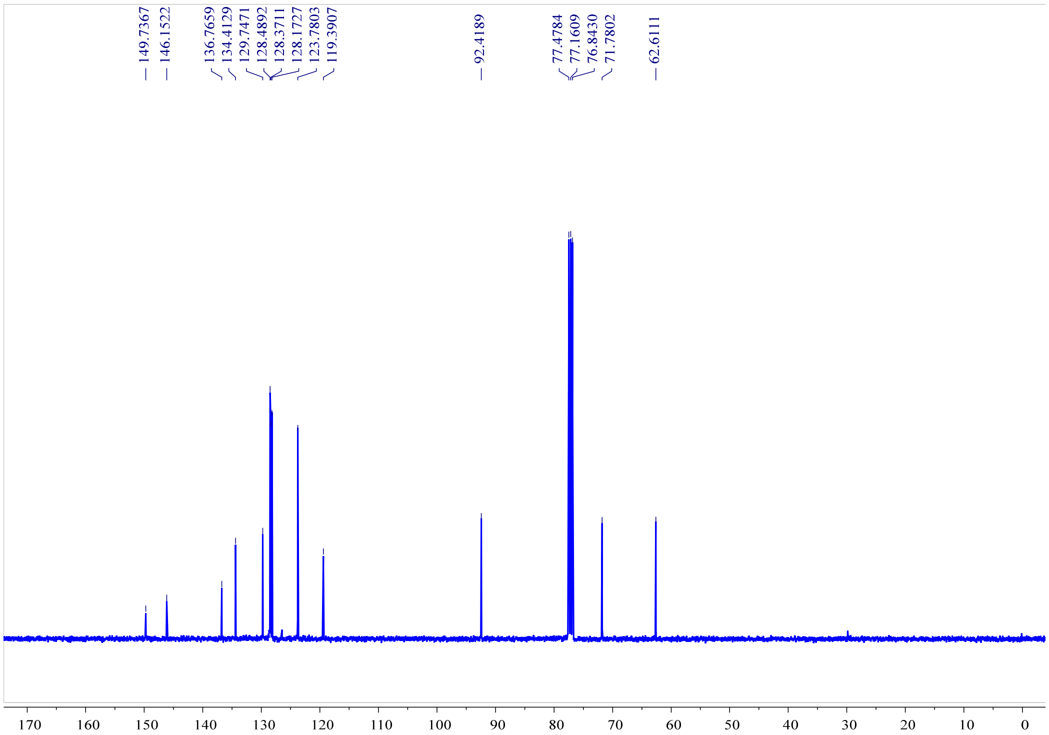
149.7367
146.1522
136.7659
134.4129
129.7471
128.4892
128.3711
128.1727
123.7803
119.3907
92.4189
77.4784
77.1609
76.8430
71.7802
62.6111
170 160 150 140 130 120 110 100 90 80 70 60 50 40 30 20 10 0

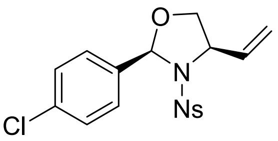
O
N
Ns
Cl

3b

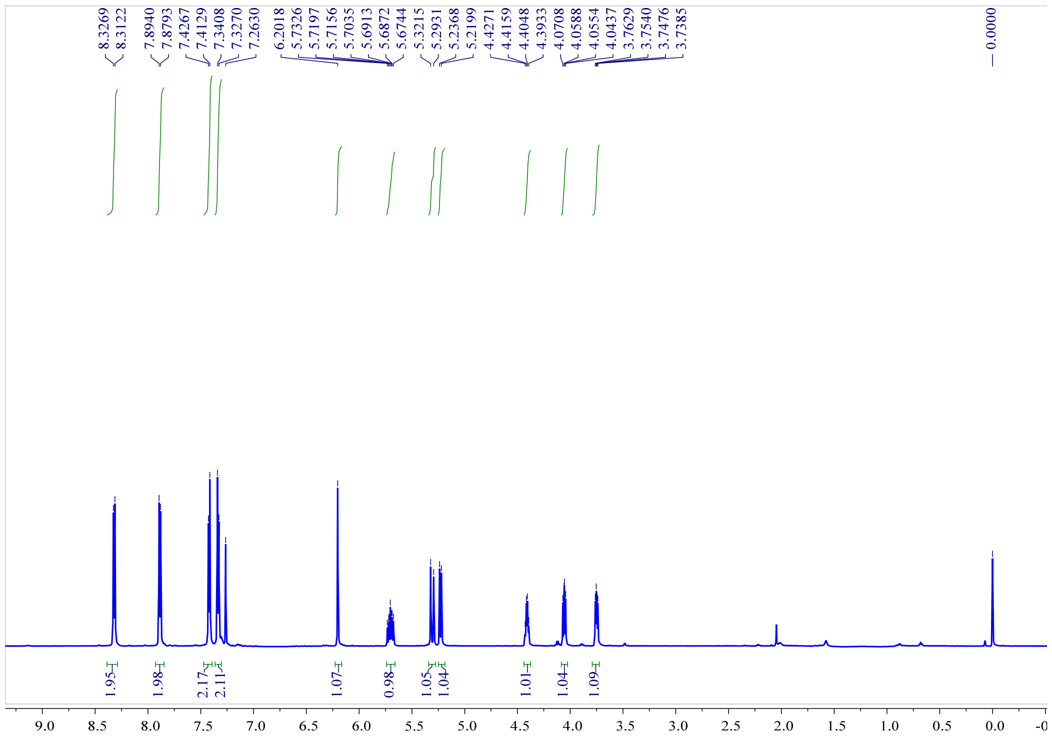
8.3269
8.3122
7.8940
7.8793
7.4267
7.4129
7.3408
7.3270
7.2630
6.2018
5.7326
5.7197
5.7156
5.7035
5.6913
5.6872
5.6744
5.3215
5.2931
5.2368
5.2199
4.4271
4.4159
4.4048
4.3933
4.0708
4.0588
4.0554
4.0437
3.7629
3.7540
3.7476
3.7385
0.0000
1.95
1.98
2.17
2.11
1.07
0.98
1.05
1.04
1.01
1.04
1.09
9.0
8.5
8.0
7.5
7.0
6.5
6.0
5.5
5.0
4.5
4.0
3.5
3.0
2.5
2.0
1.5
1.0
0.5
0.0

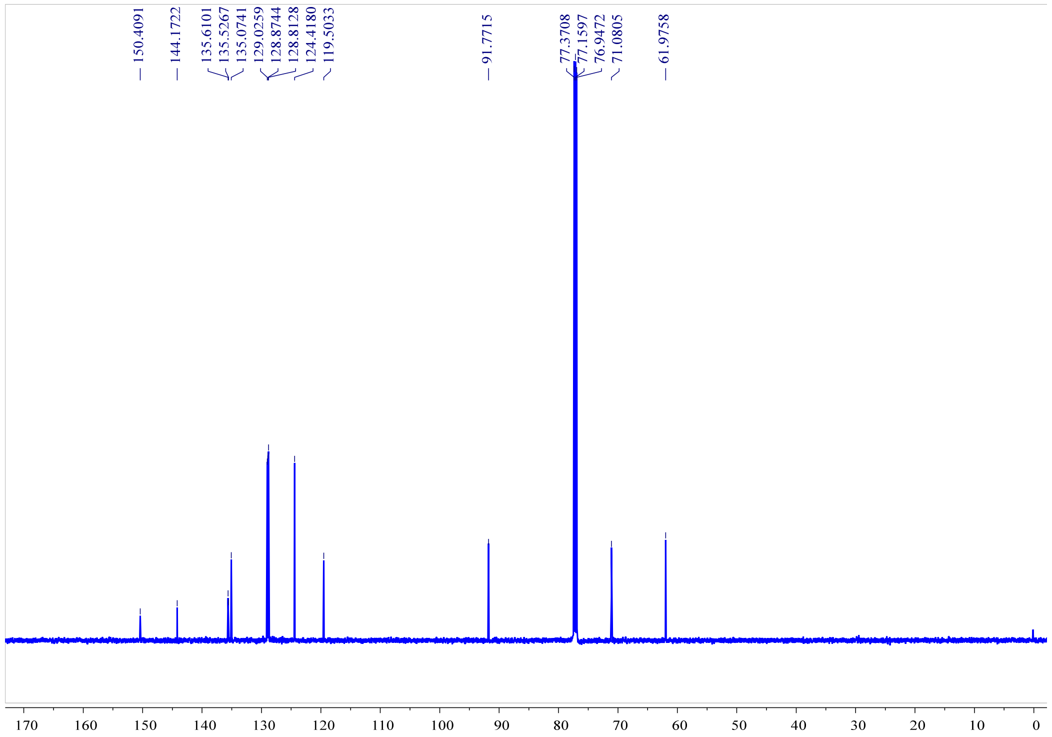
150.4091
144.1722
135.6101
135.5267
135.0741
129.0259
128.8744
128.8128
124.4180
119.5033
91.7715
77.3708
77.1597
76.9472
71.0805
61.9758
170
160
150
140
130
120
110
100
90
80
70
60
50
40
30
20
10
0

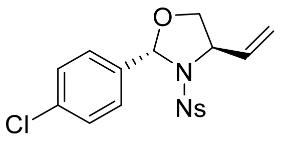
O
N
Ns
Cl

3b'

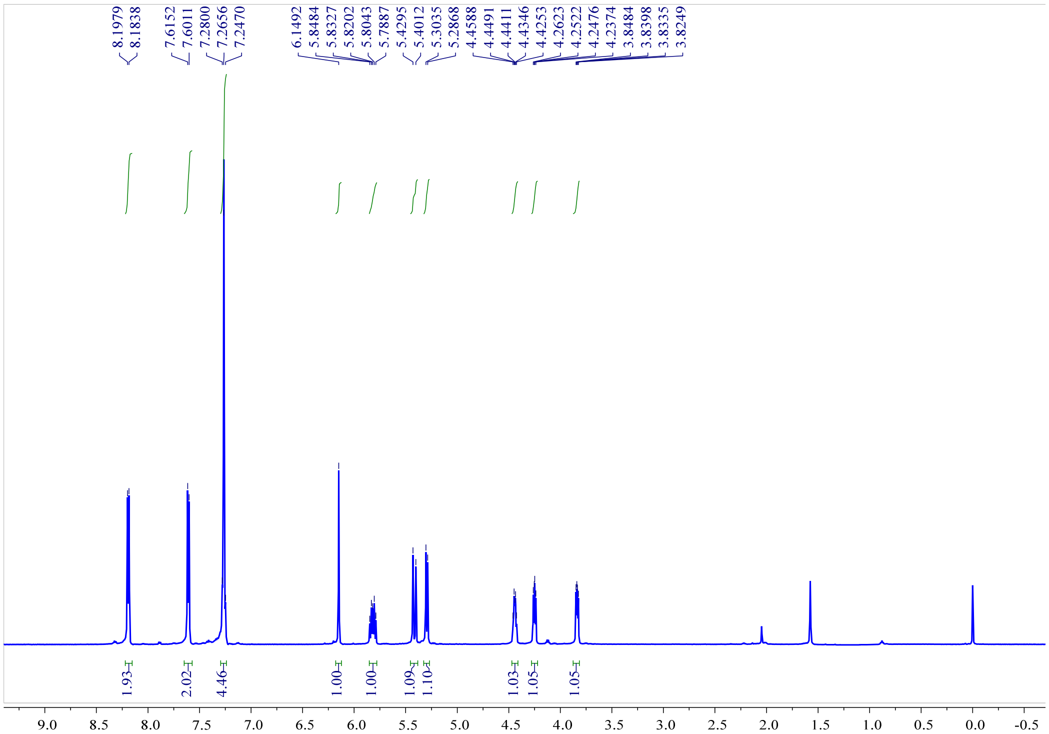
8.1979
8.1838
7.6152
7.6011
7.2800
7.2656
7.2470
6.1492
5.8484
5.8327
5.8202
5.8043
5.7887
5.4295
5.4012
5.3035
5.2868
4.4588
4.4491
4.4411
4.4346
4.4253
4.2623
4.2522
4.2476
4.2374
3.8484
3.8398
3.8335
3.8249
1.93
2.02
4.46
1.00
1.00
1.09
1.10
1.03
1.05
1.05
9.0 8.5 8.0 7.5 7.0 6.5 6.0 5.5 5.0 4.5 4.0 3.5 3.0 2.5 2.0 1.5 1.0 0.5 0.0 -0.5

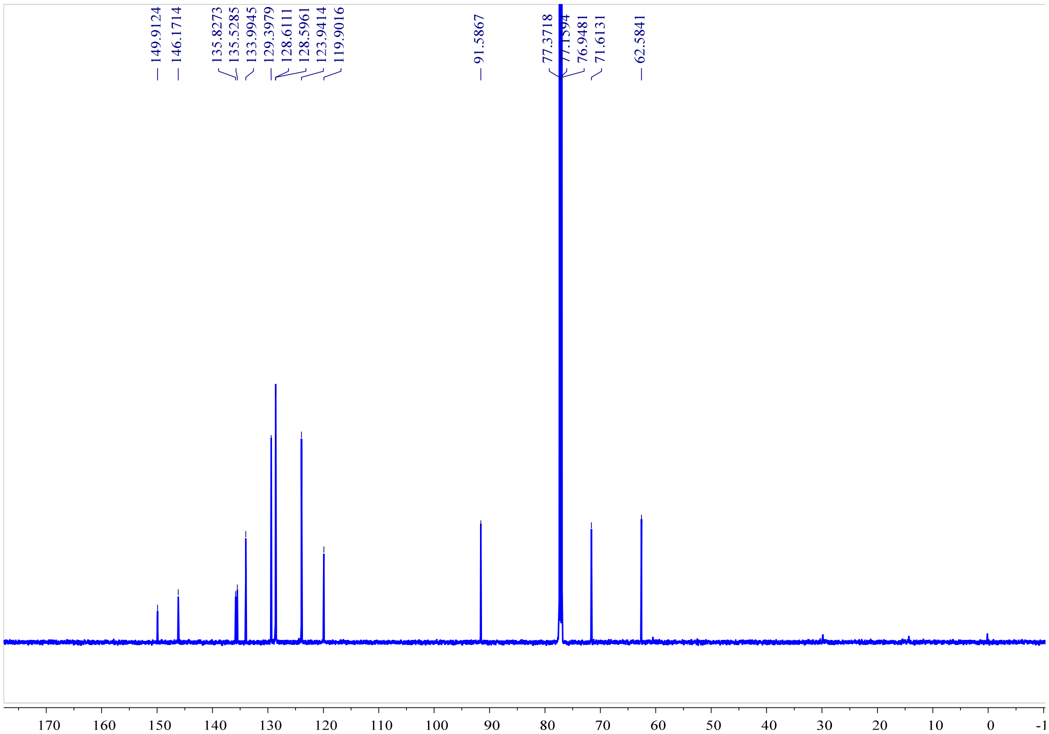
149.9124
146.1714
135.8273
135.5285
133.9945
129.3979
128.6111
128.5961
123.9414
119.9016
91.5867
77.3718
77.1594
76.9481
71.6131
62.5841
170 160 150 140 130 120 110 100 90 80 70 60 50 40 30 20 10 0 -1

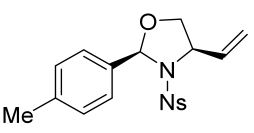
O
N
Ns
Me

3c

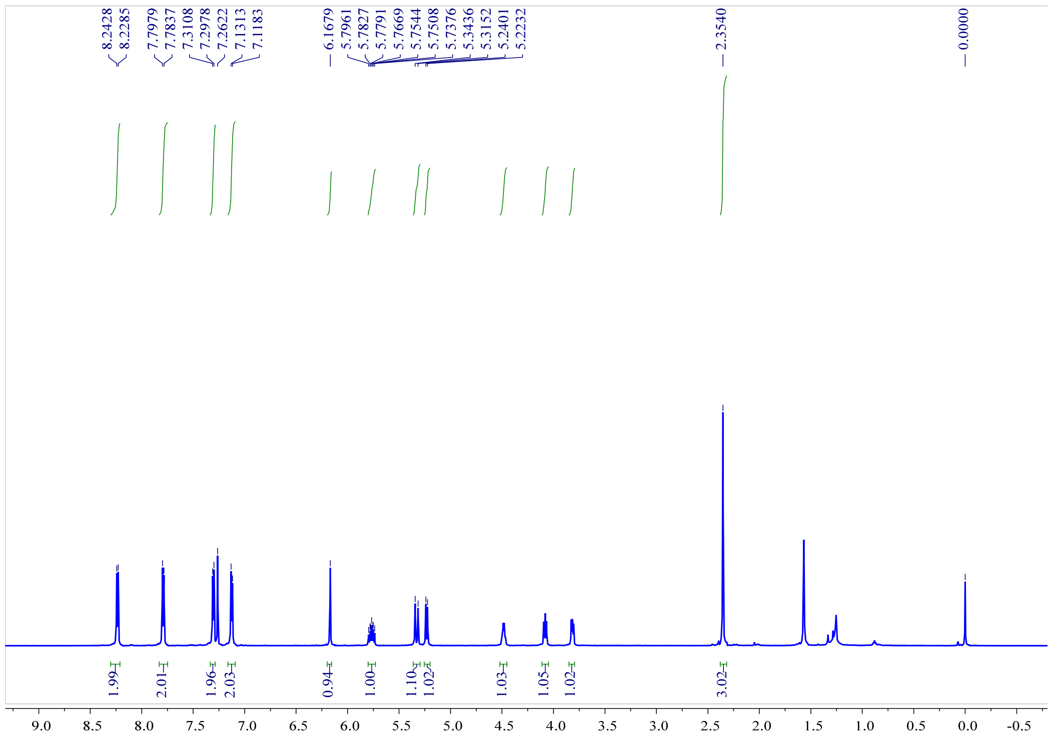
8.2428
8.2285
7.7979
7.7837
7.3108
7.2978
7.2622
7.1313
7.1183
6.1679
5.7961
5.7827
5.7791
5.7669
5.7544
5.7508
5.7376
5.3436
5.3152
5.2401
5.2232
2.3540
0.0000
1.99
2.01
1.96
2.03
0.94
1.00
1.10
1.02
1.03
1.05
1.02
3.02
9.0 8.5 8.0 7.5 7.0 6.5 6.0 5.5 5.0 4.5 4.0 3.5 3.0 2.5 2.0 1.5 1.0 0.5 0.0 -0.5

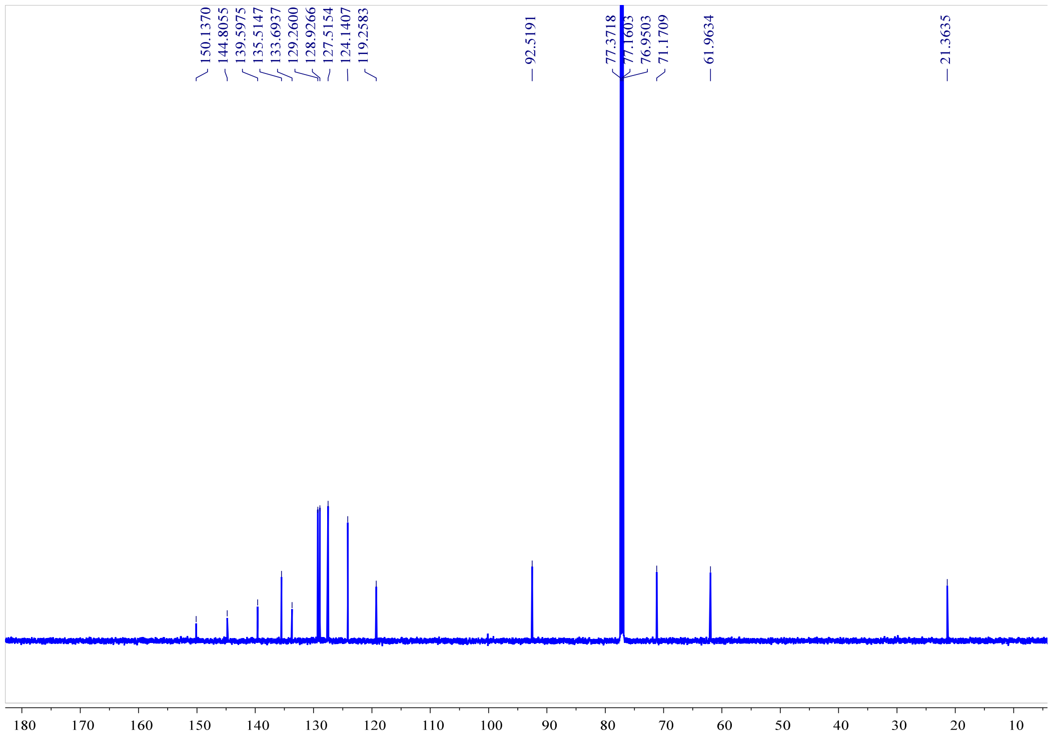
150.1370
144.8055
139.5975
135.5147
133.6937
129.2600
128.9266
127.5154
124.1407
119.2583
92.5191
77.3718
77.1603
76.9503
71.1709
61.9634
21.3635
180 170 160 150 140 130 120 110 100 90 80 70 60 50 40 30 20 10

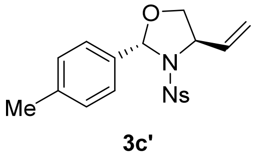
O
N
Ns
Me

3c'

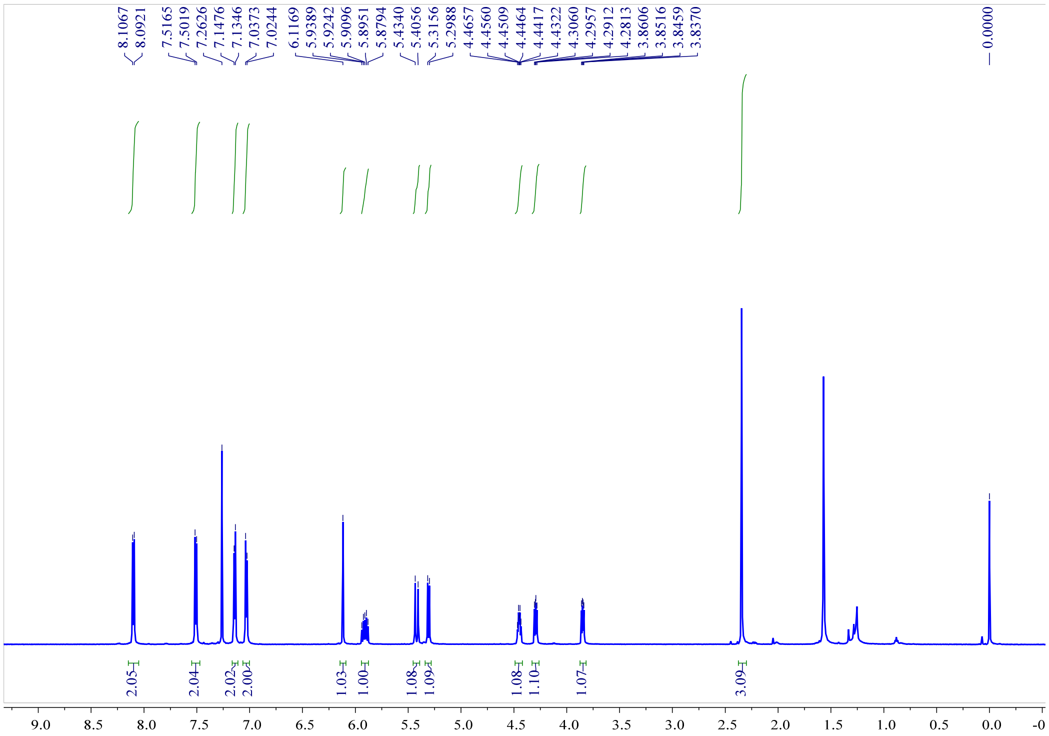

8.1067
8.0921
7.5165
7.5019
7.2626
7.1476
7.1346
7.0373
7.0244
6.1169
5.9389
5.9242
5.9096
5.8951
5.8794
5.4340
5.4056
5.3156
5.2988
4.4657
4.4560
4.4509
4.4464
4.4417
4.4322
4.3060
4.2957
4.2912
4.2813
3.8606
3.8516
3.8459
3.8370
0.0000
2.05
2.04
2.02
2.00
1.03
1.00
1.08
1.09
1.08
1.10
1.07
3.09
9.0
8.5
8.0
7.5
7.0
6.5
6.0
5.5
5.0
4.5
4.0
3.5
3.0
2.5
2.0
1.5
1.0
0.5
0.0
-0

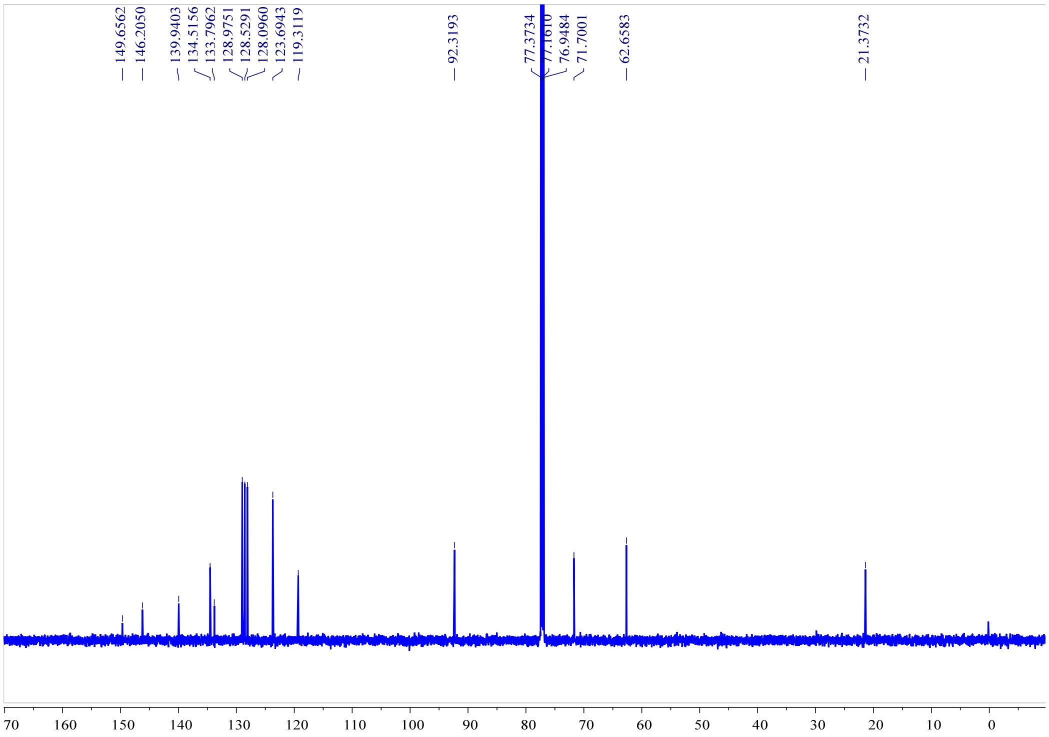

149.6562
146.2050
139.9403
134.5156
133.7962
128.9751
128.5291
128.0960
123.6943
119.3119
92.3193
77.3734
77.1610
76.9484
71.7001
62.6583
21.3732
70
160
150
140
130
120
110
100
90
80
70
60
50
40
30
20
10
0

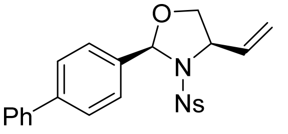

O
N
Ns
Ph

3d

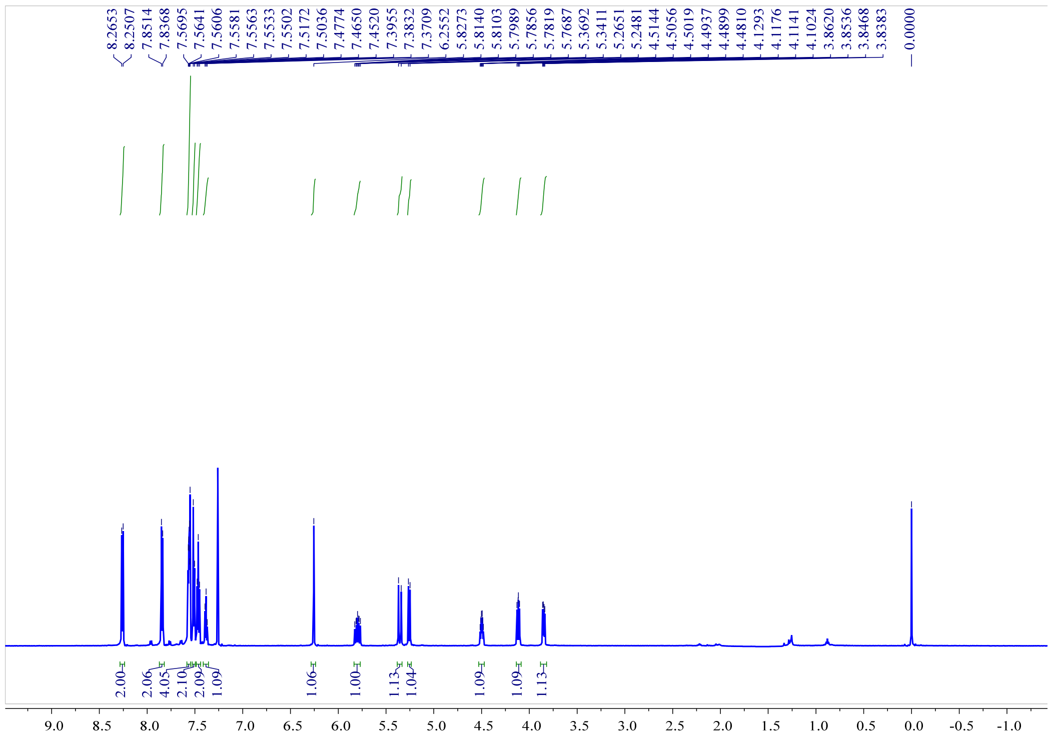

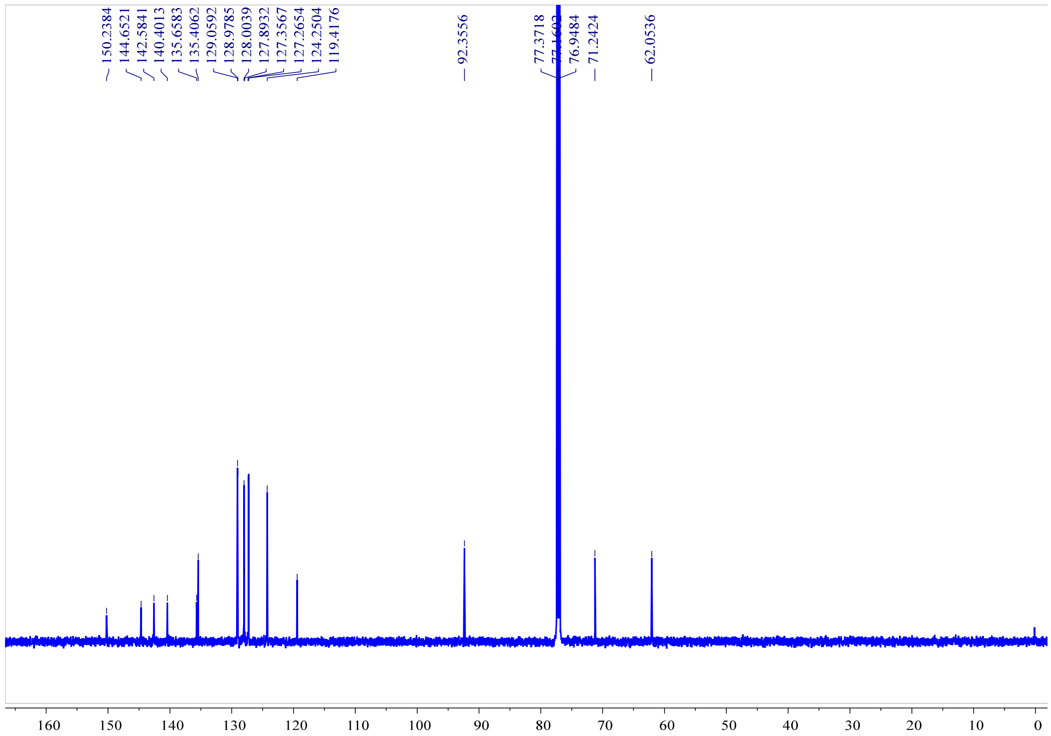

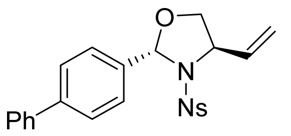

3d'

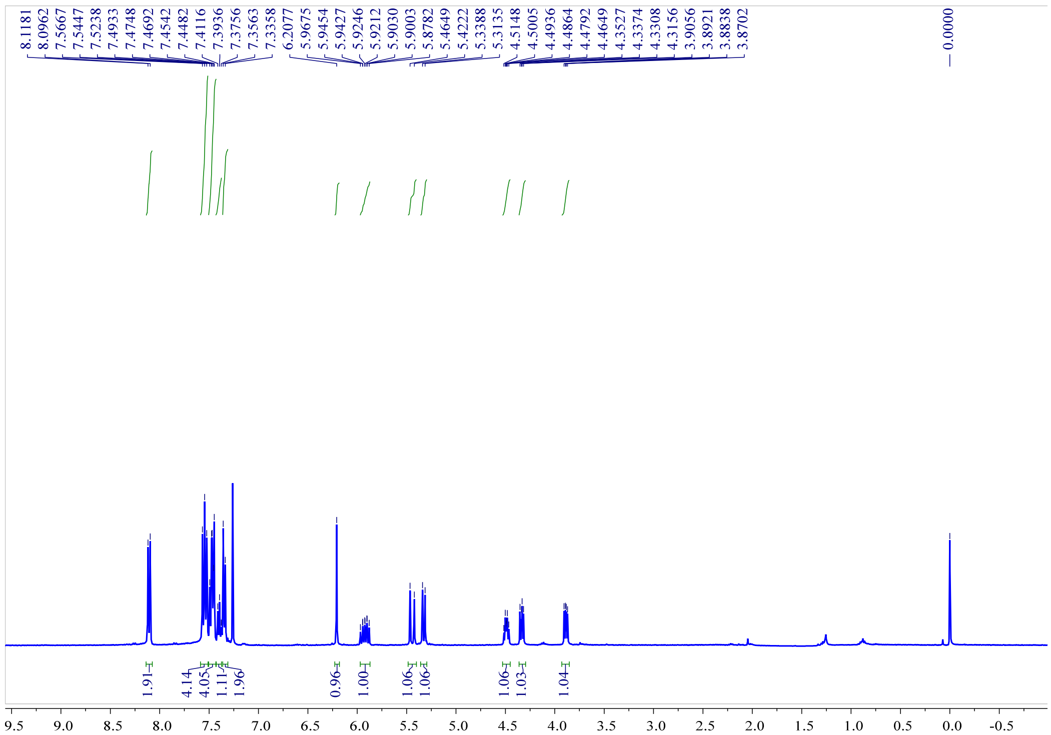
8.1181
8.0962
7.5667
7.5447
7.5238
7.4933
7.4748
7.4692
7.4542
7.4482
7.4116
7.3936
7.3756
7.3563
7.3358
6.2077
5.9675
5.9454
5.9427
5.9246
5.9212
5.9030
5.9003
5.8782
5.4649
5.4222
5.3388
5.3135
4.5148
4.5005
4.4936
4.4864
4.4792
4.4649
4.3527
4.3374
4.3308
4.3156
3.9056
3.8921
3.8838
3.8702
0.0000
1.91
4.14
4.05
1.11
1.96
0.96
1.00
1.06
1.06
1.06
1.03
1.04
9.5 9.0 8.5 8.0 7.5 7.0 6.5 6.0 5.5 5.0 4.5 4.0 3.5 3.0 2.5 2.0 1.5 1.0 0.5 0.0 -0.5

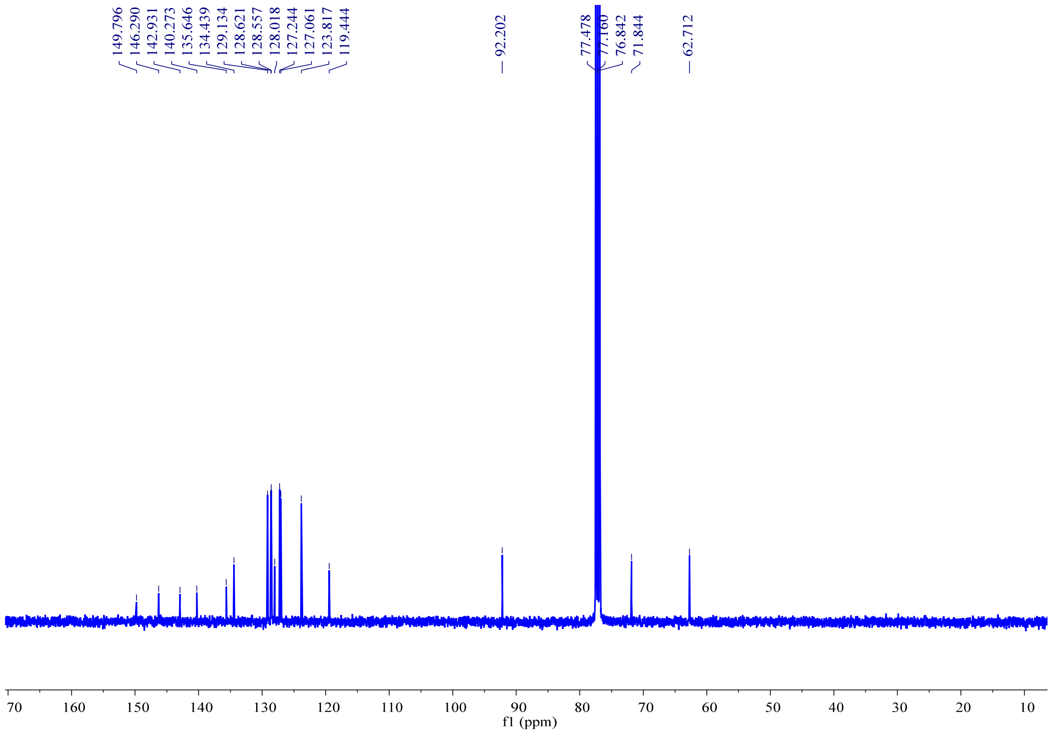
149.796
146.290
142.931
140.273
135.646
134.439
129.134
128.621
128.557
128.018
127.244
127.061
123.817
119.444
92.202
77.478
77.160
76.842
71.844
62.712
70 160 150 140 130 120 110 100 90 80 70 60 50 40 30 20 10
f1 (ppm)

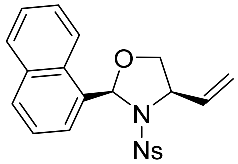
O
N
Ns

3e

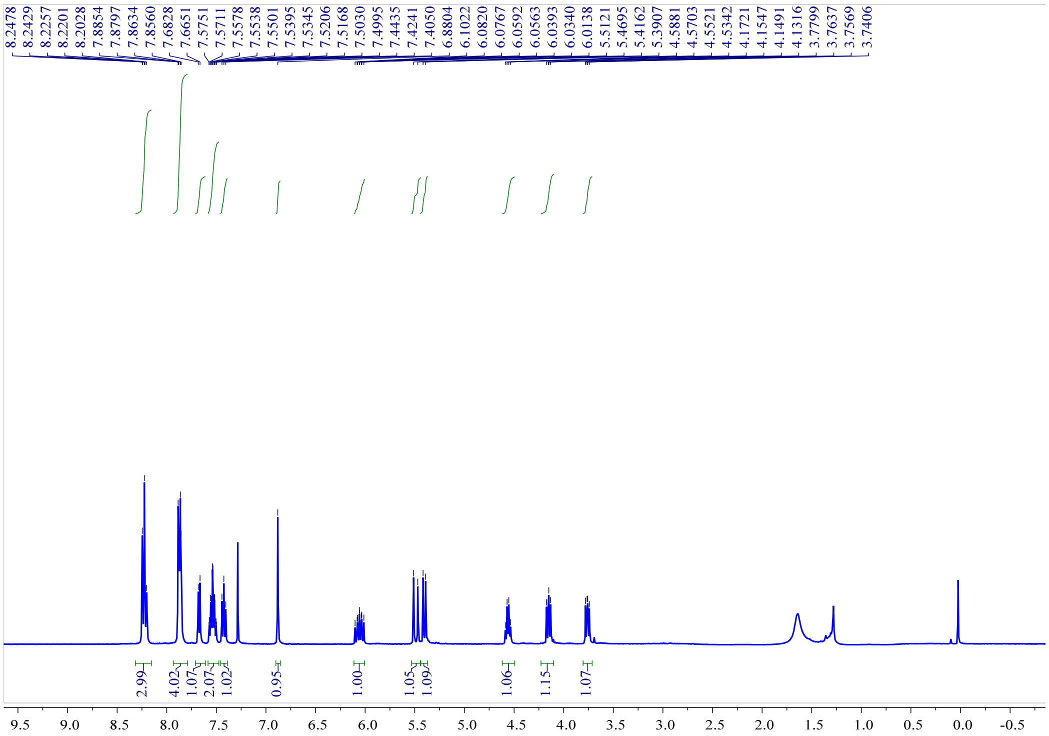

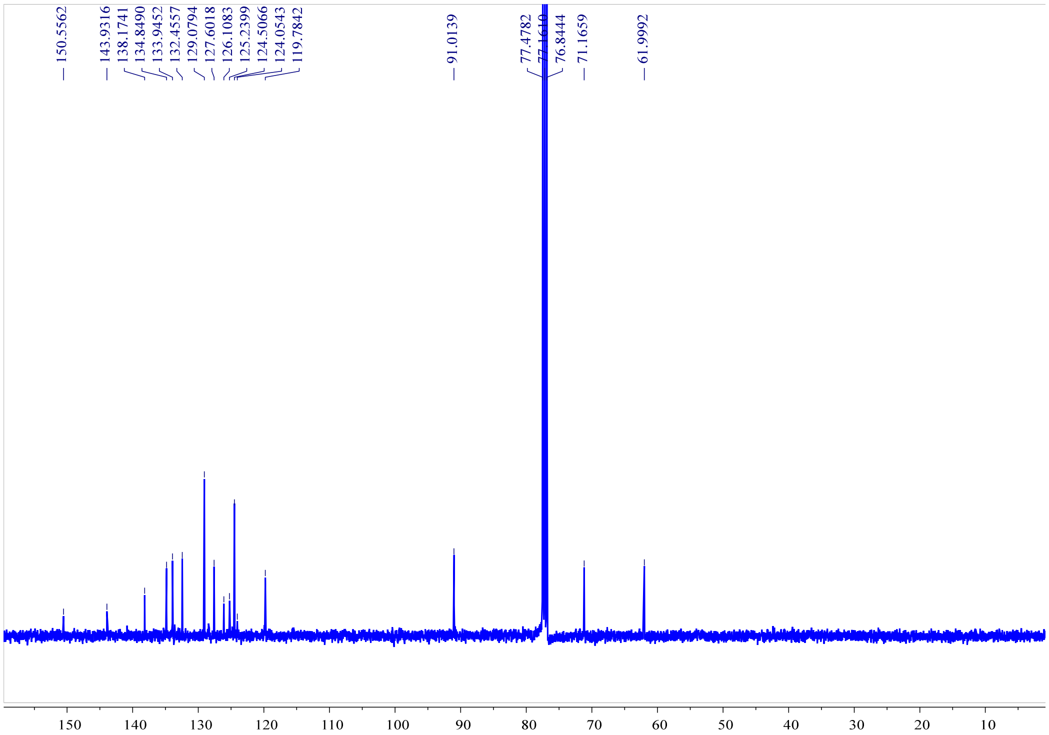

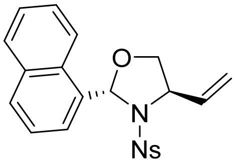

3e'

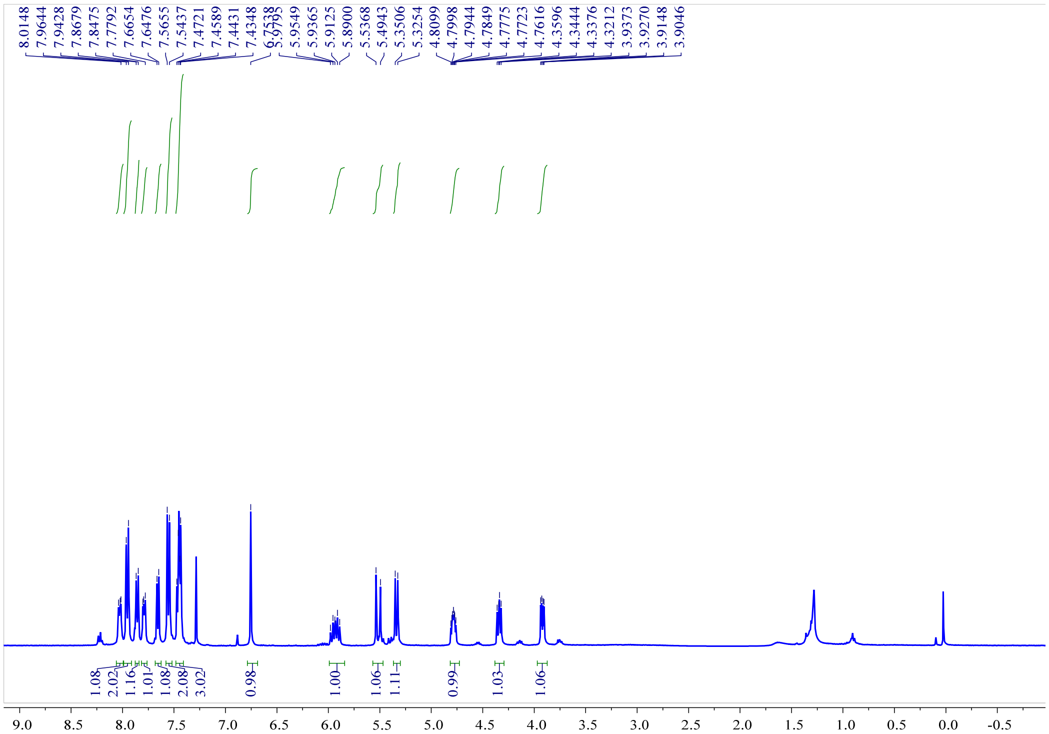
8.0148
7.9644
7.9428
7.8679
7.8475
7.7792
7.6654
7.6476
7.5655
7.5437
7.4721
7.4589
7.4431
7.4348
6.7538
5.9795
5.9549
5.9365
5.9125
5.8900
5.5368
5.4943
5.3506
5.3254
4.8099
4.7998
4.7944
4.7849
4.7775
4.7723
4.7616
4.3596
4.3444
4.3376
4.3212
3.9373
3.9270
3.9148
3.9046
1.08
2.02
1.16
1.01
1.08
2.08
3.02
0.98
1.00
1.06
1.11
0.99
1.03
1.06
9.0 8.5 8.0 7.5 7.0 6.5 6.0 5.5 5.0 4.5 4.0 3.5 3.0 2.5 2.0 1.5 1.0 0.5 0.0 -0.5

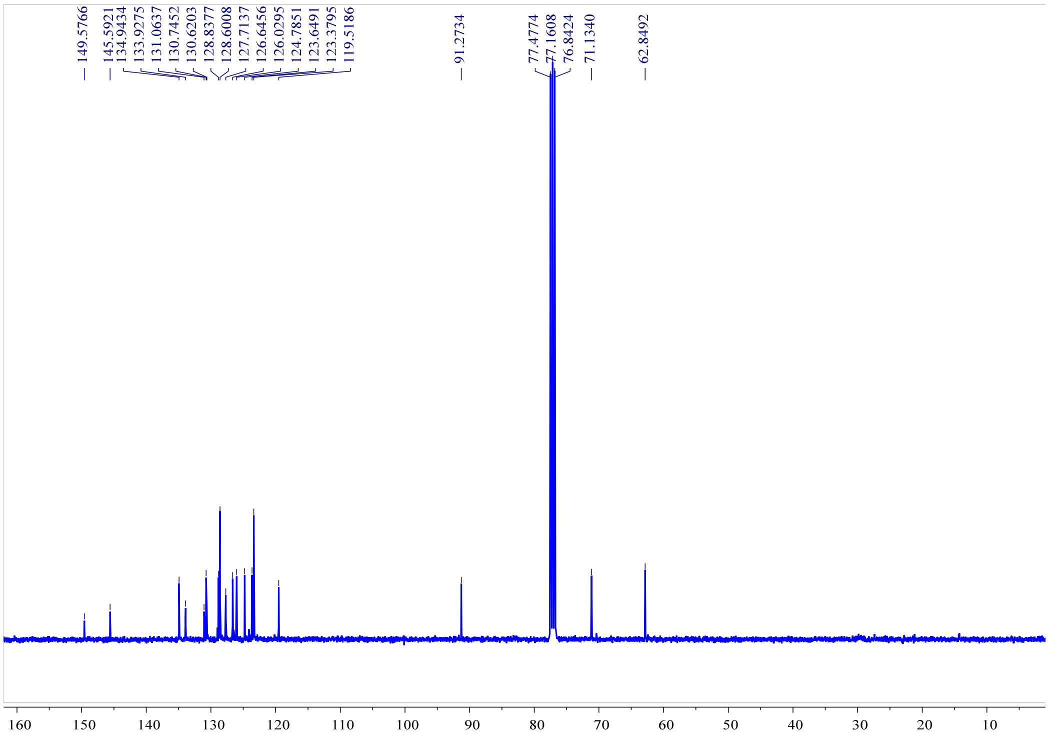
149.5766
145.5921
134.9434
133.9275
131.0637
130.7452
130.6203
128.8377
128.6008
127.7137
126.6456
126.0295
124.7851
123.6491
123.3795
119.5186
91.2734
77.4774
77.1608
76.8424
71.1340
62.8492
160 150 140 130 120 110 100 90 80 70 60 50 40 30 20 10

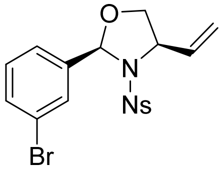
O
N
Ns
Br

3f

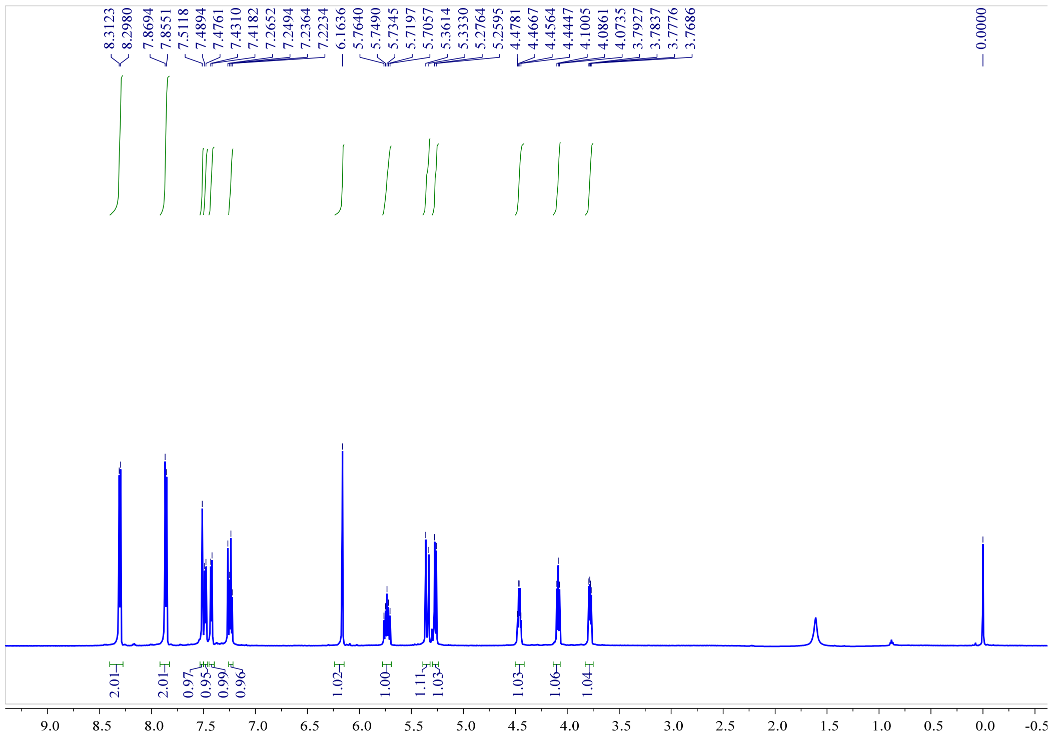
8.3123
8.2980
7.8694
7.8551
7.5118
7.4894
7.4761
7.4310
7.4182
7.2652
7.2494
7.2364
7.2234
6.1636
5.7640
5.7490
5.7345
5.7197
5.7057
5.3614
5.3330
5.2764
5.2595
4.4781
4.4667
4.4564
4.4447
4.1005
4.0861
4.0735
3.7927
3.7837
3.7776
3.7686
0.0000
2.01
2.01
0.97
0.95
0.99
0.96
1.02
1.00
1.11
1.03
1.03
1.06
1.04
9.0
8.5
8.0
7.5
7.0
6.5
6.0
5.5
5.0
4.5
4.0
3.5
3.0
2.5
2.0
1.5
1.0
0.5
0.0
-0.5

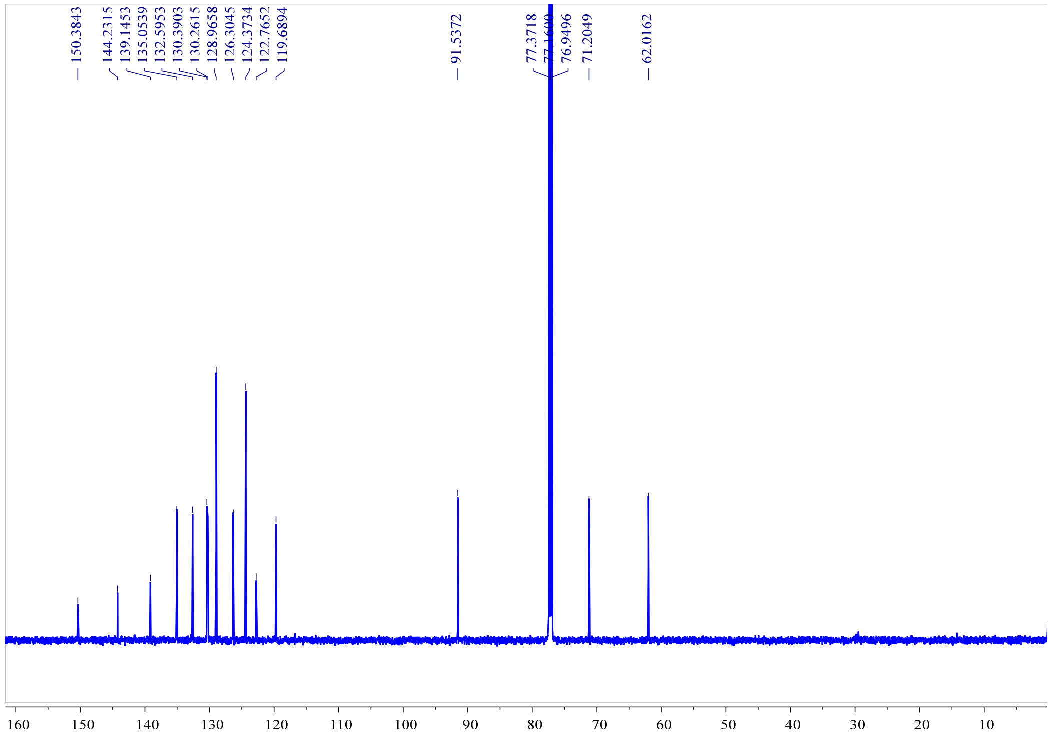
150.3843
144.2315
139.1453
135.0539
132.5953
130.3903
130.2615
128.9658
126.3045
124.3734
122.7652
119.6894
91.5372
77.3718
76.9496
71.2049
62.0162
160
150
140
130
120
110
100
90
80
70
60
50
40
30
20
10

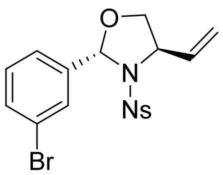
O
N
Ns
Br

3f'

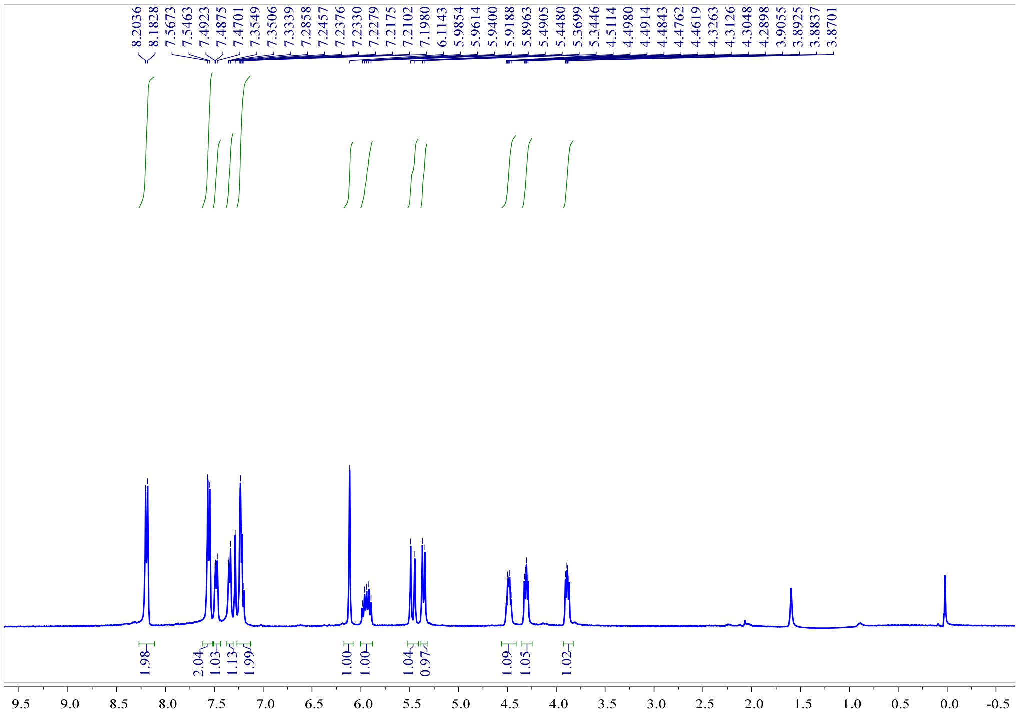
8.2036
8.1828
7.5673
7.5463
7.4923
7.4875
7.4701
7.3549
7.3506
7.3339
7.2858
7.2457
7.2376
7.2330
7.2279
7.2175
7.2102
7.1980
6.1143
5.9854
5.9614
5.9400
5.9188
5.8963
5.4905
5.4480
5.3699
5.3446
4.5114
4.4980
4.4914
4.4843
4.4762
4.4619
4.3263
4.3126
4.3048
4.2898
3.9055
3.8925
3.8837
3.8701
1.98
2.04
1.03
1.13
1.99
1.00
1.00
1.04
0.97
1.09
1.05
1.02
9.5 9.0 8.5 8.0 7.5 7.0 6.5 6.0 5.5 5.0 4.5 4.0 3.5 3.0 2.5 2.0 1.5 1.0 0.5 0.0 -0.5

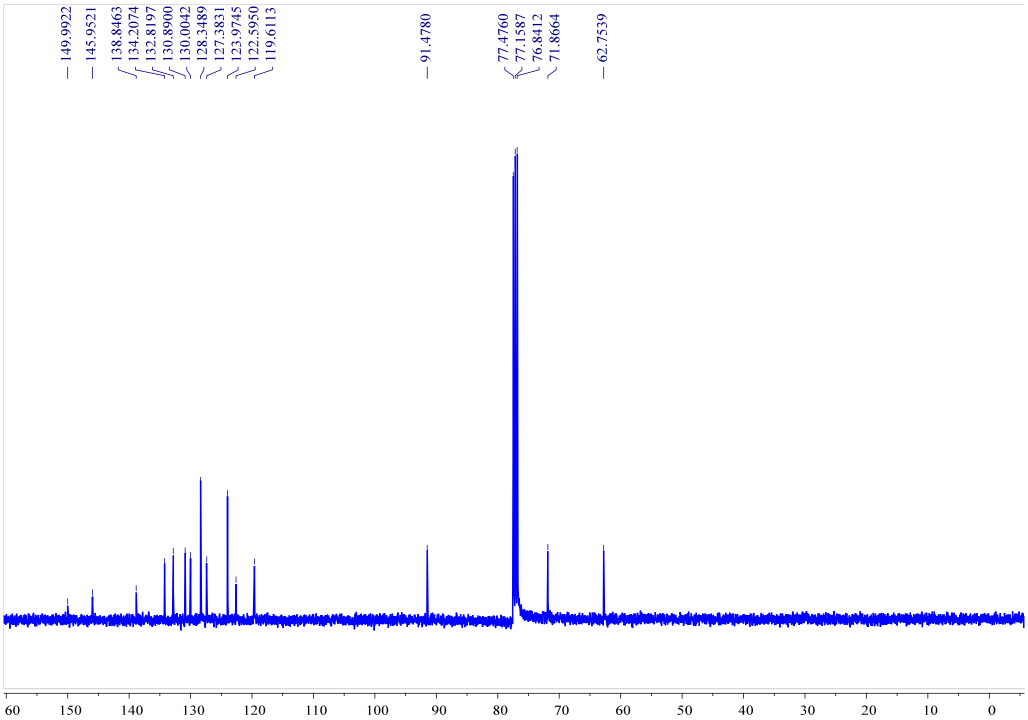
149.9922
145.9521
138.8463
134.2074
132.8197
130.8900
130.0042
128.3489
127.3831
123.9745
122.5950
119.6113
91.4780
77.4760
77.1587
76.8412
71.8664
62.7539
60 150 140 130 120 110 100 90 80 70 60 50 40 30 20 10 0

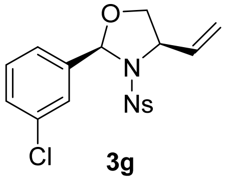
O
N
Ns
Cl

3g

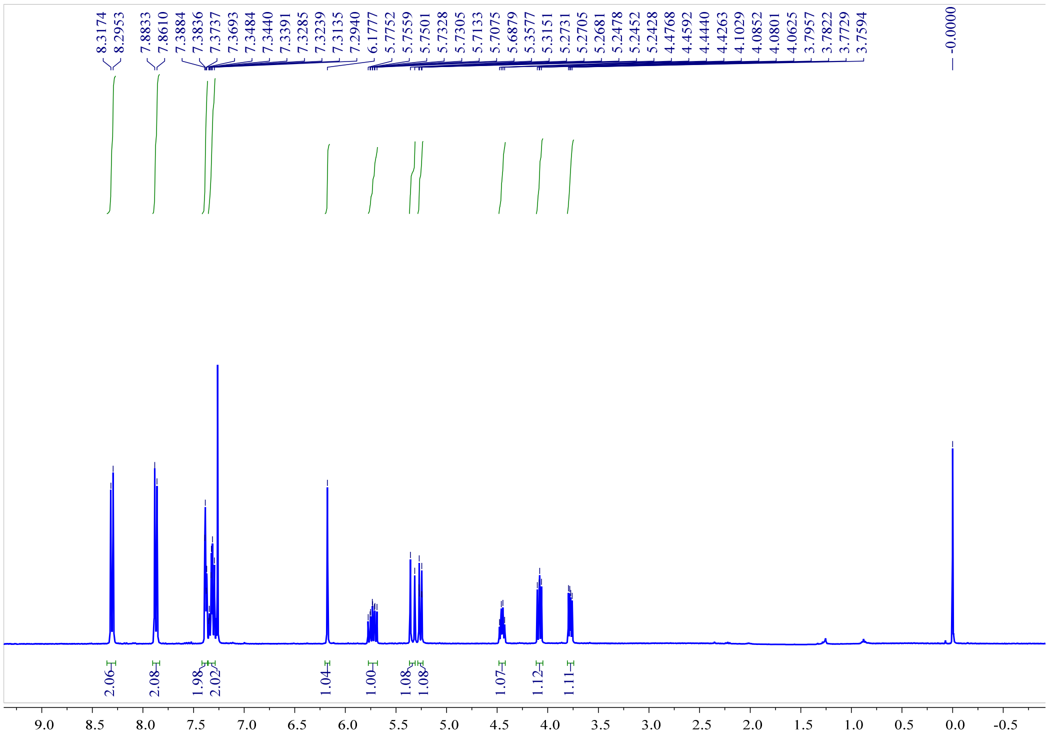
8.3174
8.2953
7.8833
7.8610
7.3884
7.3836
7.3737
7.3693
7.3484
7.3440
7.3391
7.3285
7.3239
7.3135
7.2940
6.1777
5.7752
5.7559
5.7501
5.7328
5.7305
5.7133
5.7075
5.6879
5.3577
5.3151
5.2731
5.2705
5.2681
5.2478
5.2452
5.2428
4.4768
4.4592
4.4440
4.4263
4.1029
4.0852
4.0801
4.0625
3.7957
3.7822
3.7729
3.7594
-0.0000
2.06
2.08
1.98
2.02
1.04
1.00
1.08
1.08
1.07
1.12
1.11
9.0
8.5
8.0
7.5
7.0
6.5
6.0
5.5
5.0
4.5
4.0
3.5
3.0
2.5
2.0
1.5
1.0
0.5
0.0
-0.5

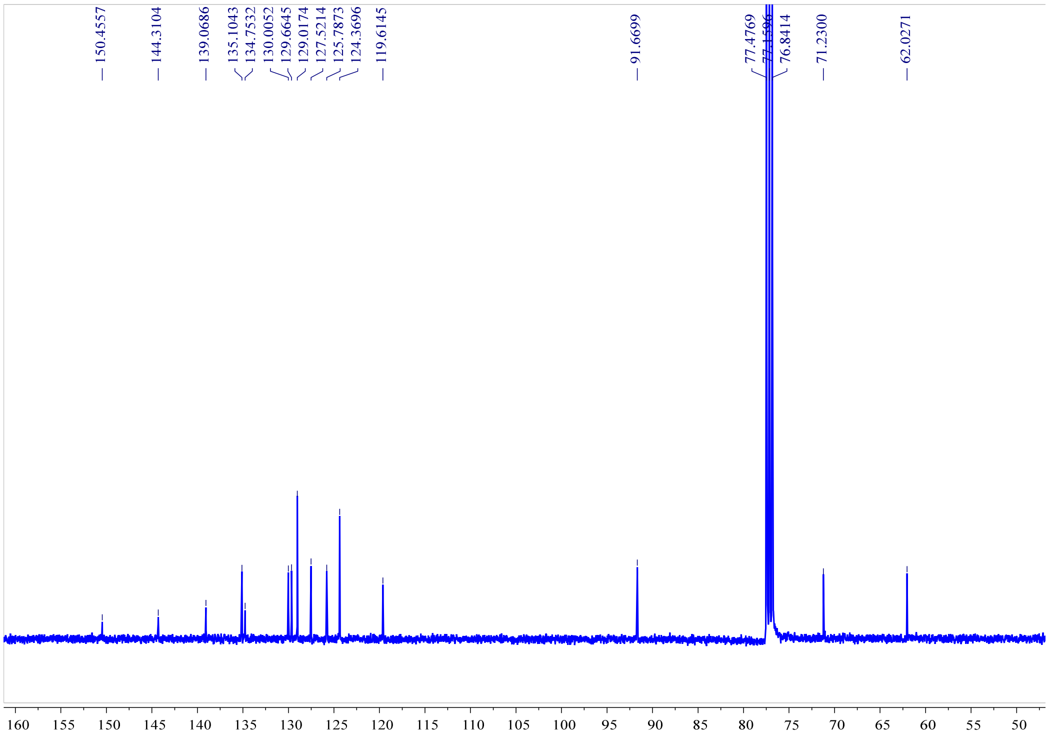
150.4557
144.3104
139.0686
135.1043
134.7532
130.0052
129.6645
129.0174
127.5214
125.7873
124.3696
119.6145
91.6699
77.4769
76.8414
71.2300
62.0271
160
155
150
145
140
135
130
125
120
115
110
105
100
95
90
85
80
75
70
65
60
55
50

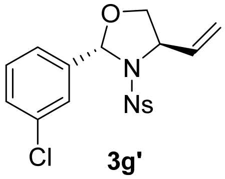
O
N
Ns
Cl

3g'

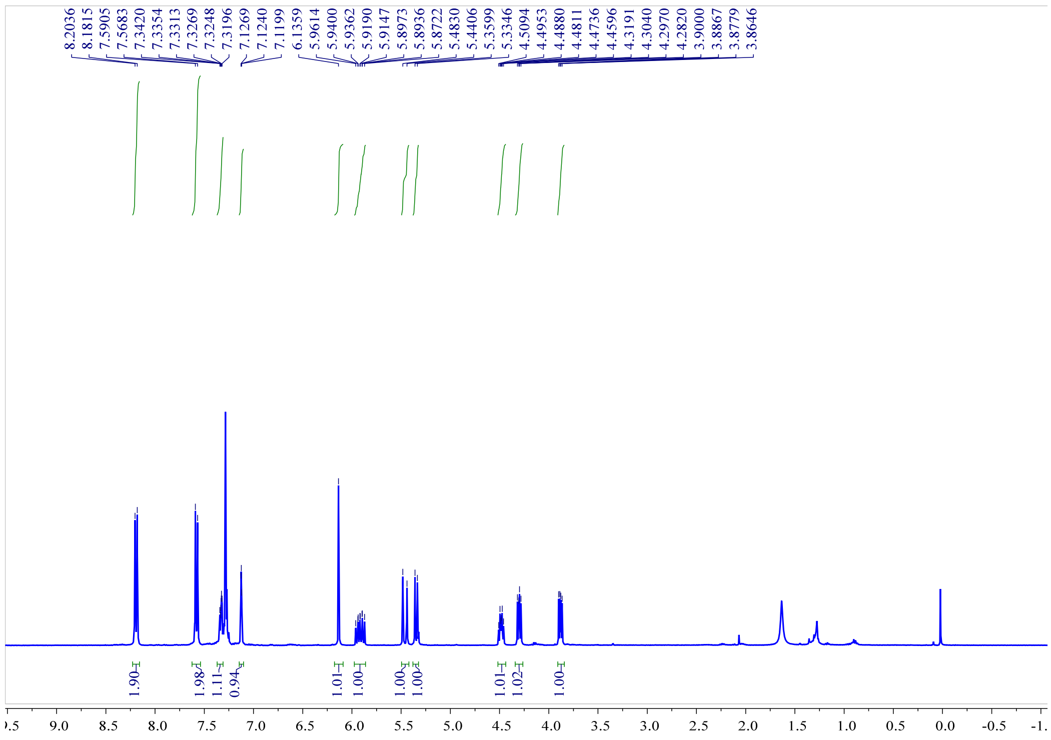

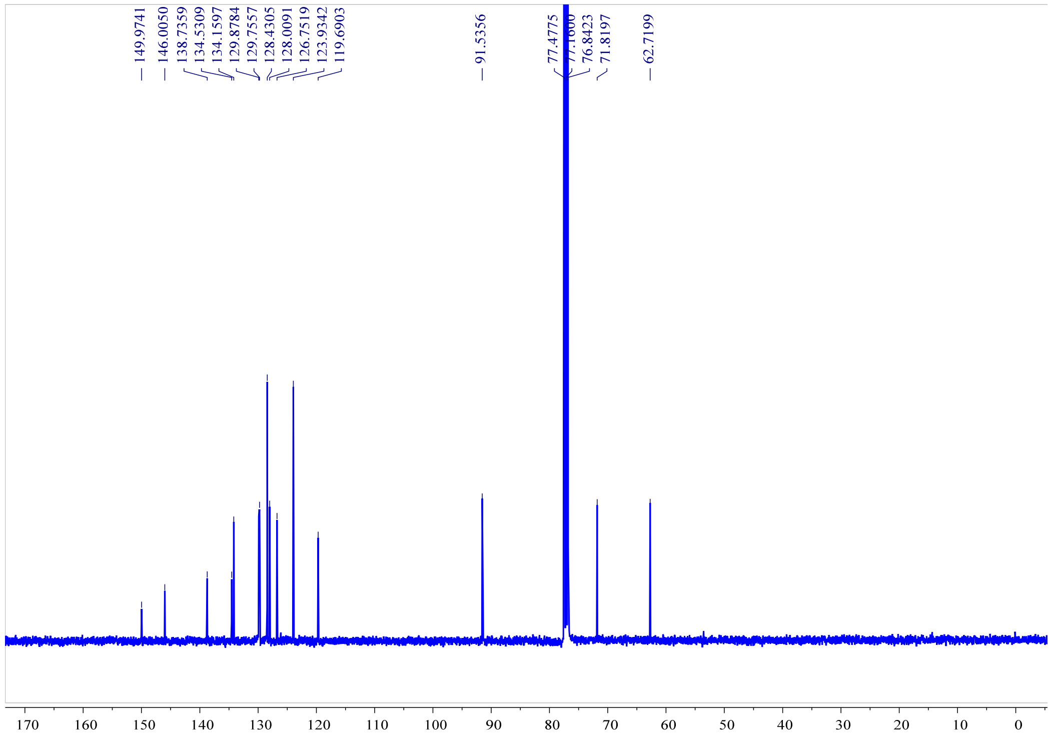

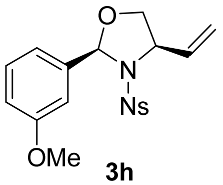

3h

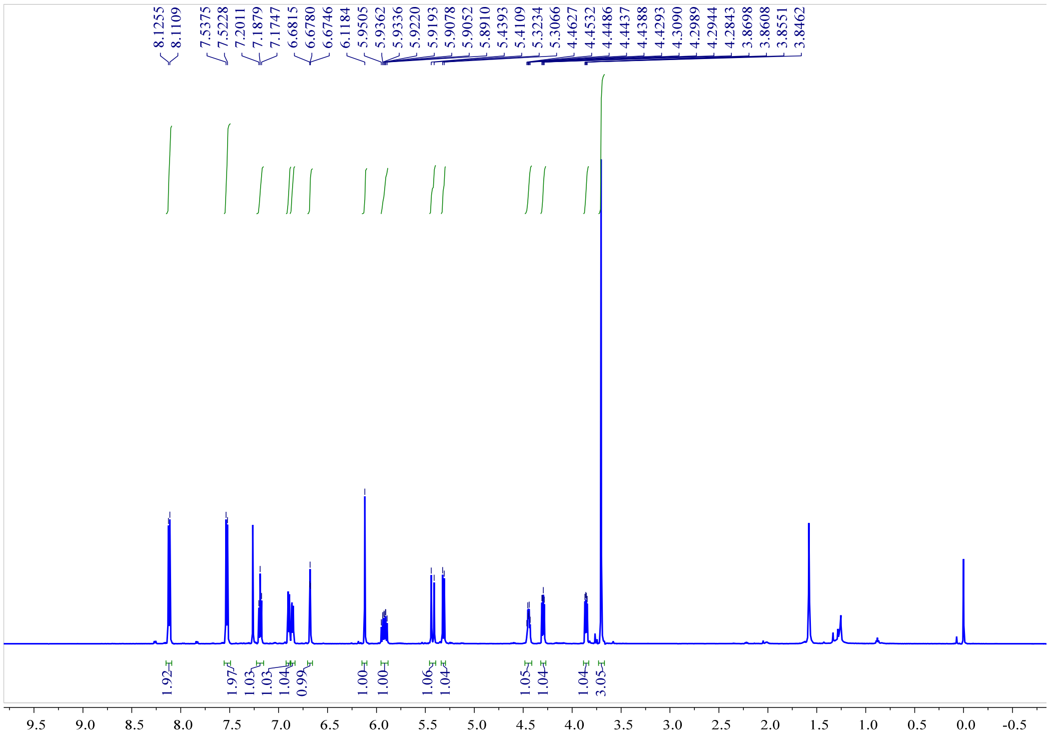
8.1255
8.1109
7.5375
7.5228
7.2011
7.1879
7.1747
6.6815
6.6780
6.6746
6.1184
5.9505
5.9362
5.9336
5.9220
5.9193
5.9078
5.9052
5.8910
5.4393
5.4109
5.3234
5.3066
4.4627
4.4532
4.4486
4.4437
4.4388
4.4293
4.3090
4.2989
4.2944
4.2843
3.8698
3.8608
3.8551
3.8462
1.92
1.97
1.03
1.03
1.04
0.99
1.00
1.00
1.06
1.04
1.05
1.04
1.04
3.05
9.5 9.0 8.5 8.0 7.5 7.0 6.5 6.0 5.5 5.0 4.5 4.0 3.5 3.0 2.5 2.0 1.5 1.0 0.5 0.0 -0.5

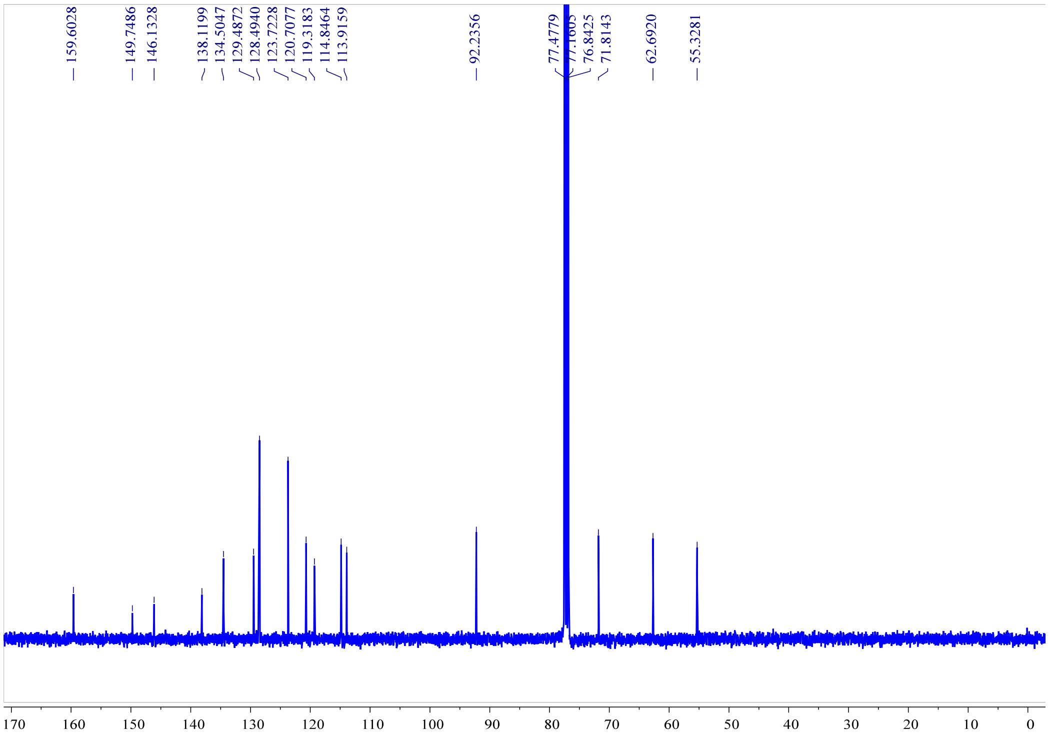
159.6028
149.7486
146.1328
138.1199
134.5047
129.4872
128.4940
123.7228
120.7077
119.3183
114.8464
113.9159
92.2356
77.4779
76.8425
71.8143
62.6920
55.3281
170 160 150 140 130 120 110 100 90 80 70 60 50 40 30 20 10 0

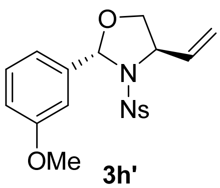
O
N
Ns
OMe

3h'

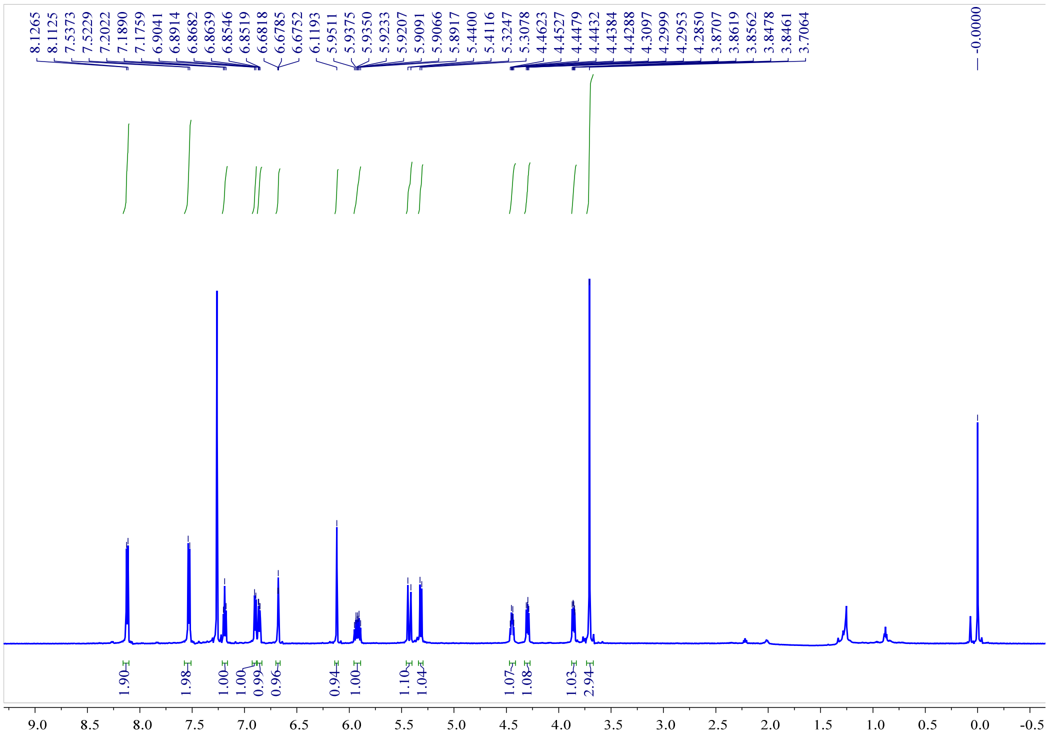
8.1265
8.1125
7.5373
7.5229
7.2022
7.1890
7.1759
6.9041
6.8914
6.8682
6.8639
6.8546
6.8519
6.6818
6.6785
6.6752
6.1193
5.9511
5.9375
5.9350
5.9233
5.9207
5.9091
5.9066
5.8917
5.4400
5.4116
5.3247
5.3078
4.4623
4.4527
4.4479
4.4432
4.4384
4.4288
4.3097
4.2999
4.2953
4.2850
3.8707
3.8619
3.8562
3.8478
3.8461
3.7064
-0.0000
1.90
1.98
1.00
1.00
0.99
0.96
0.94
1.00
1.10
1.04
1.07
1.08
1.03
2.94
9.0 8.5 8.0 7.5 7.0 6.5 6.0 5.5 5.0 4.5 4.0 3.5 3.0 2.5 2.0 1.5 1.0 0.5 0.0 -0.5

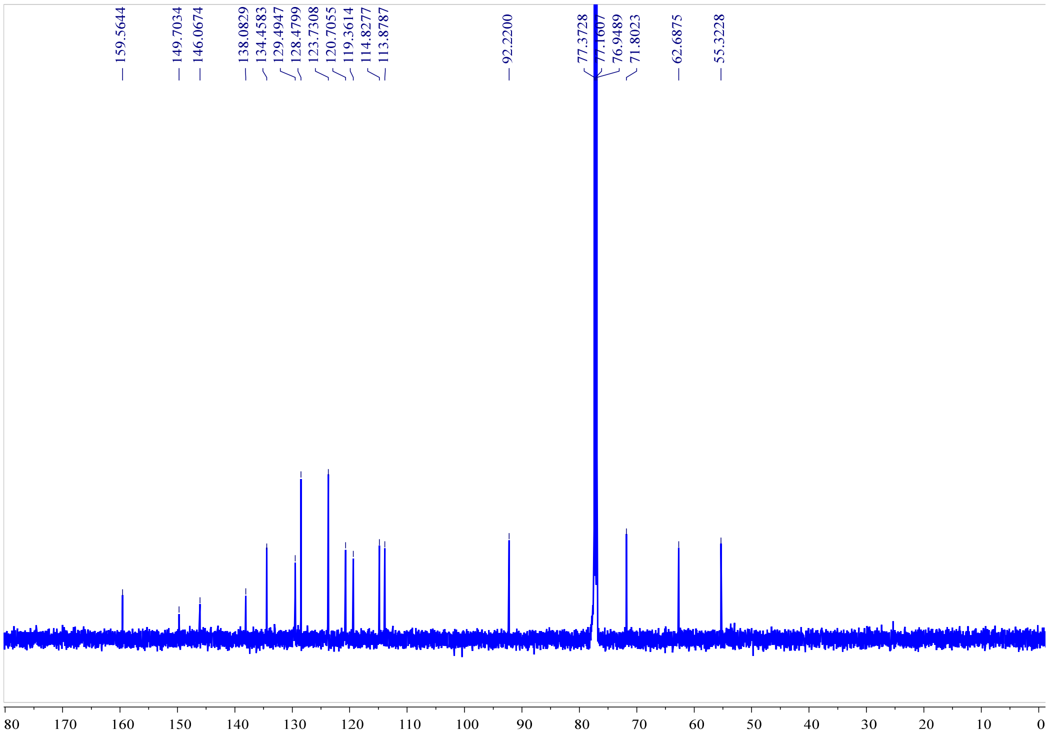
159.5644
149.7034
146.0674
138.0829
134.4583
129.4947
128.4799
123.7308
120.7055
119.3614
114.8277
113.8787
92.2200
77.3728
77.1607
76.9489
71.8023
62.6875
55.3228
180 170 160 150 140 130 120 110 100 90 80 70 60 50 40 30 20 10 0

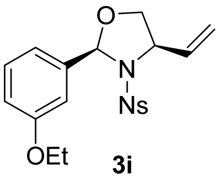
O
N
Ns
OEt
3i

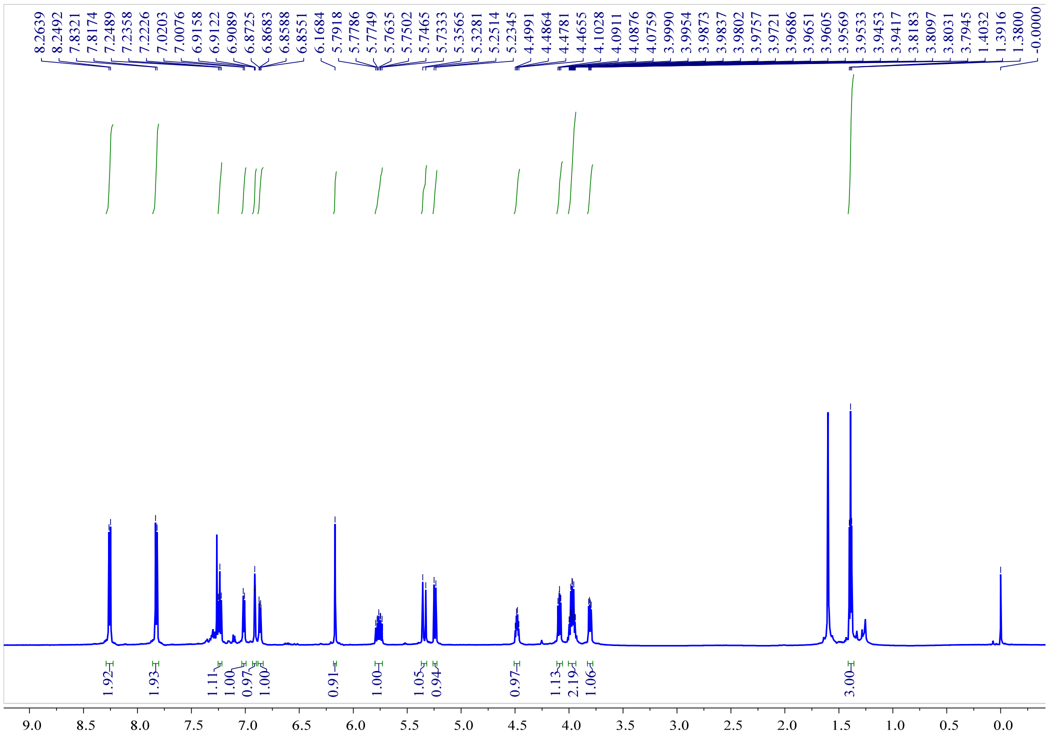
8.2639
8.2492
7.8321
7.8174
7.2489
7.2358
7.2226
7.0203
7.0076
6.9158
6.9122
6.9089
6.8725
6.8683
6.8588
6.8551
6.1684
5.7918
5.7786
5.7749
5.7635
5.7502
5.7465
5.7333
5.3565
5.3281
5.2514
5.2345
4.4991
4.4864
4.4781
4.4655
4.1028
4.0911
4.0876
4.0759
3.9990
3.9954
3.9873
3.9837
3.9802
3.9757
3.9721
3.9686
3.9651
3.9605
3.9569
3.9533
3.9453
3.9417
3.8183
3.8097
3.8031
3.7945
1.4032
1.3916
1.3800
-0.0000
1.92
1.93
1.11
1.00
0.97
1.00
0.91
1.00
1.05
0.94
0.97
1.13
2.19
1.06
3.00
9.0 8.5 8.0 7.5 7.0 6.5 6.0 5.5 5.0 4.5 4.0 3.5 3.0 2.5 2.0 1.5 1.0 0.5 0.0

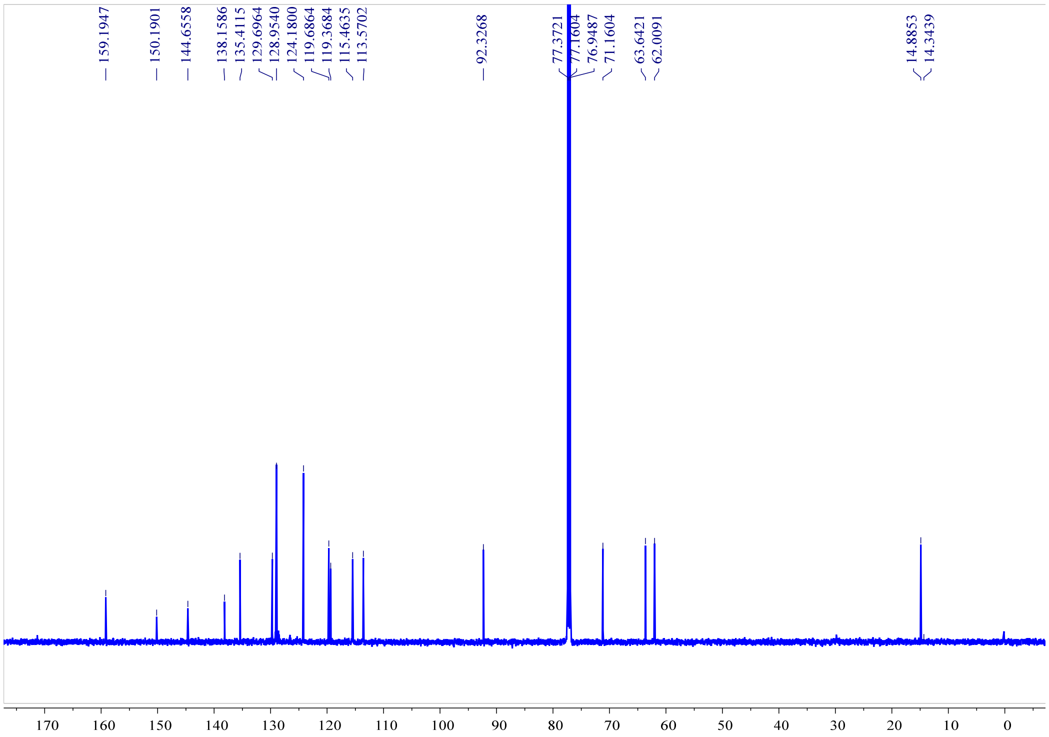
159.1947
150.1901
144.6558
138.1586
135.4115
129.6964
128.9540
124.1800
119.6864
119.3684
115.4635
113.5702
92.3268
77.3721
77.1604
76.9487
71.1604
63.6421
62.0091
14.8853
14.3439
170 160 150 140 130 120 110 100 90 80 70 60 50 40 30 20 10 0

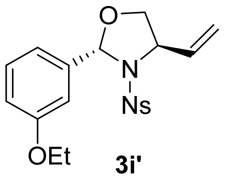
O
N
Ns
OEt

3i'

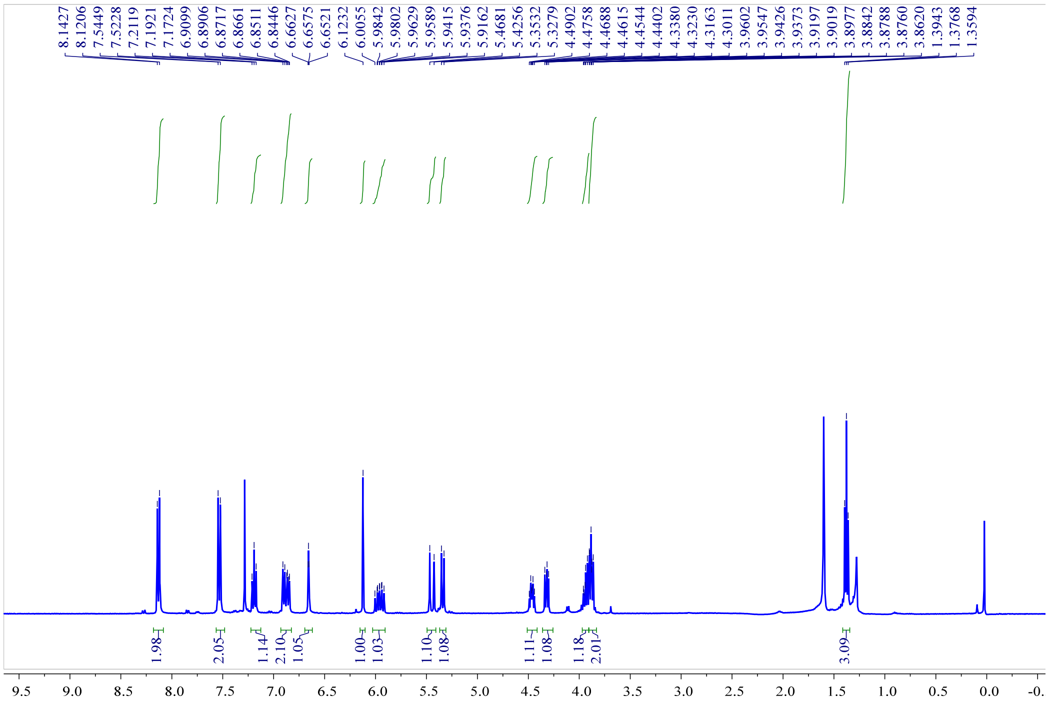
8.1427
8.1206
7.5449
7.5228
7.2119
7.1921
7.1724
6.9099
6.8906
6.8717
6.8661
6.8511
6.8446
6.6627
6.6575
6.6521
6.1232
6.0055
5.9842
5.9802
5.9629
5.9589
5.9415
5.9376
5.9162
5.4681
5.4256
5.3532
5.3279
4.4902
4.4758
4.4688
4.4615
4.4544
4.4402
4.3380
4.3230
4.3163
4.3011
3.9602
3.9547
3.9426
3.9373
3.9197
3.9019
3.8977
3.8842
3.8788
3.8760
3.8620
1.3943
1.3768
1.3594
1.98
2.05
1.14
2.10
1.05
1.00
1.03
1.10
1.08
1.11
1.08
1.18
2.01
3.09
9.5 9.0 8.5 8.0 7.5 7.0 6.5 6.0 5.5 5.0 4.5 4.0 3.5 3.0 2.5 2.0 1.5 1.0 0.5 0.0 -0.

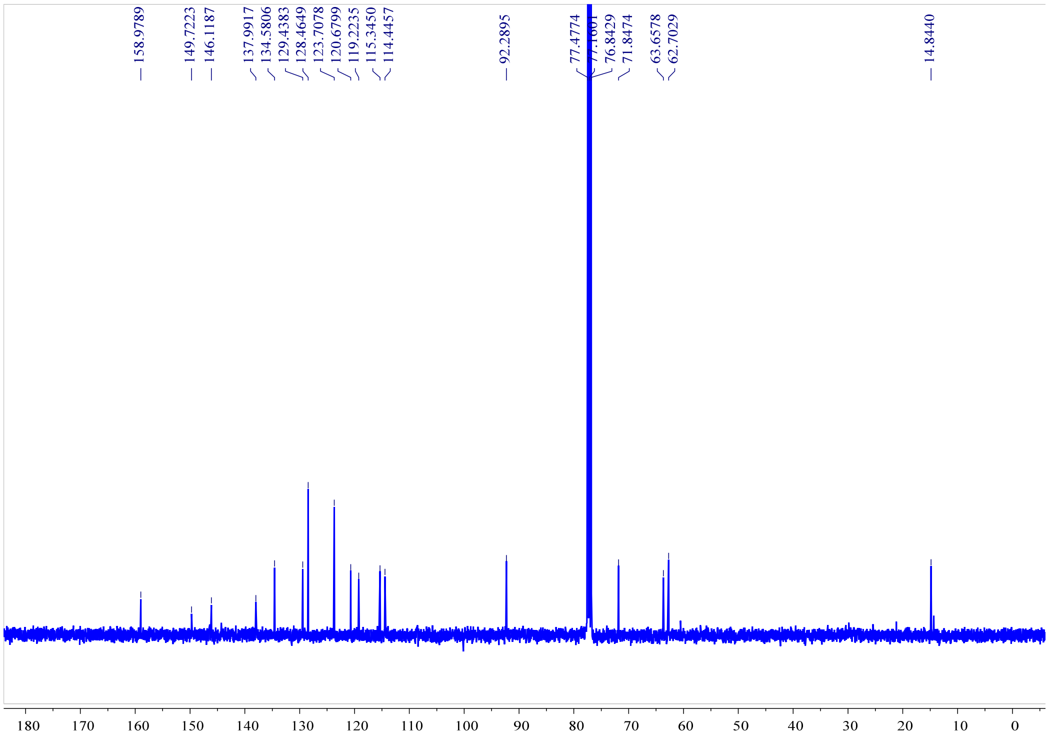
158.9789
149.7223
146.1187
137.9917
134.5806
129.4383
128.4649
123.7078
120.6799
119.2235
115.3450
114.4457
92.2895
77.4774
77.1601
76.8429
71.8474
63.6578
62.7029
14.8440
180 170 160 150 140 130 120 110 100 90 80 70 60 50 40 30 20 10 0

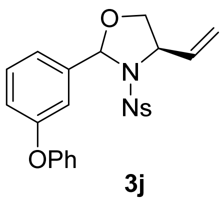
O
N
Ns
OPh

3j

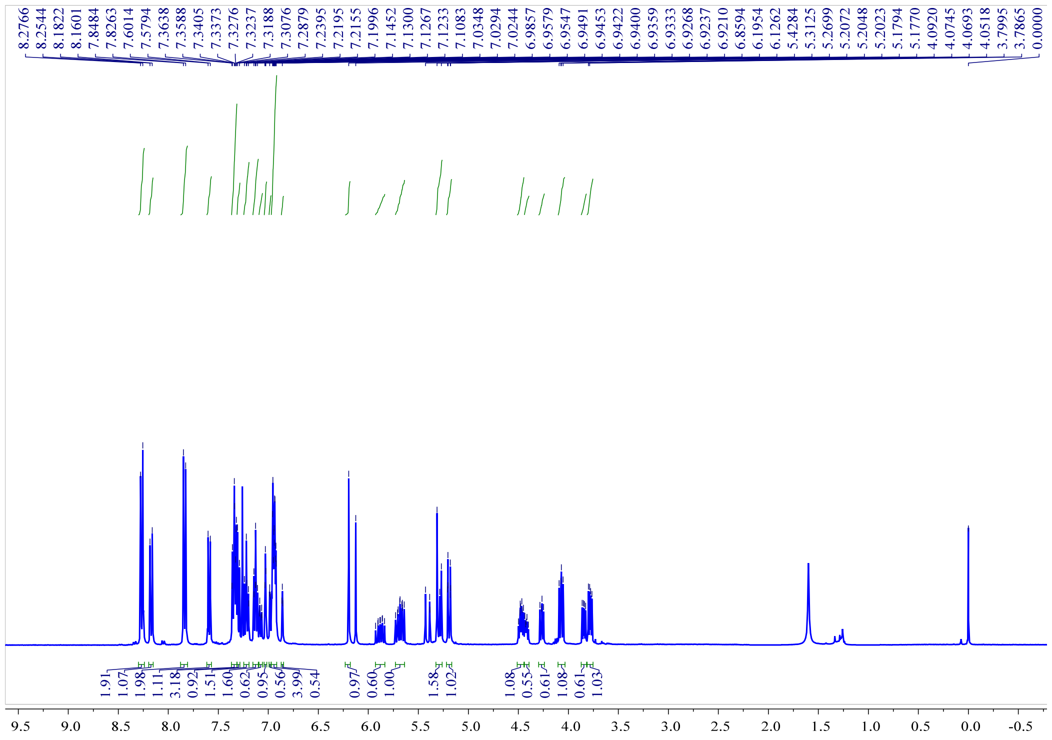
8.2766
8.2544
8.1822
8.1601
7.8484
7.8263
7.6014
7.5794
7.3638
7.3588
7.3405
7.3373
7.3276
7.3237
7.3188
7.3076
7.2879
7.2395
7.2195
7.2155
7.1996
7.1452
7.1300
7.1267
7.1233
7.1083
7.0348
7.0294
7.0244
6.9857
6.9579
6.9547
6.9491
6.9453
6.9422
6.9400
6.9359
6.9333
6.9268
6.9237
6.9210
6.8594
6.1954
6.1262
5.4284
5.3125
5.2699
5.2072
5.2048
5.2023
5.1794
5.1770
4.0920
4.0745
4.0693
4.0518
3.7995
3.7865
0.0000
1.91
1.07
1.98
1.11
3.18
0.92
1.51
1.60
0.62
0.95
0.56
3.99
0.54
0.97
0.60
1.00
1.58
1.02
1.08
0.55
0.61
1.08
0.61
1.03
9.5 9.0 8.5 8.0 7.5 7.0 6.5 6.0 5.5 5.0 4.5 4.0 3.5 3.0 2.5 2.0 1.5 1.0 0.5 0.0 -0.5
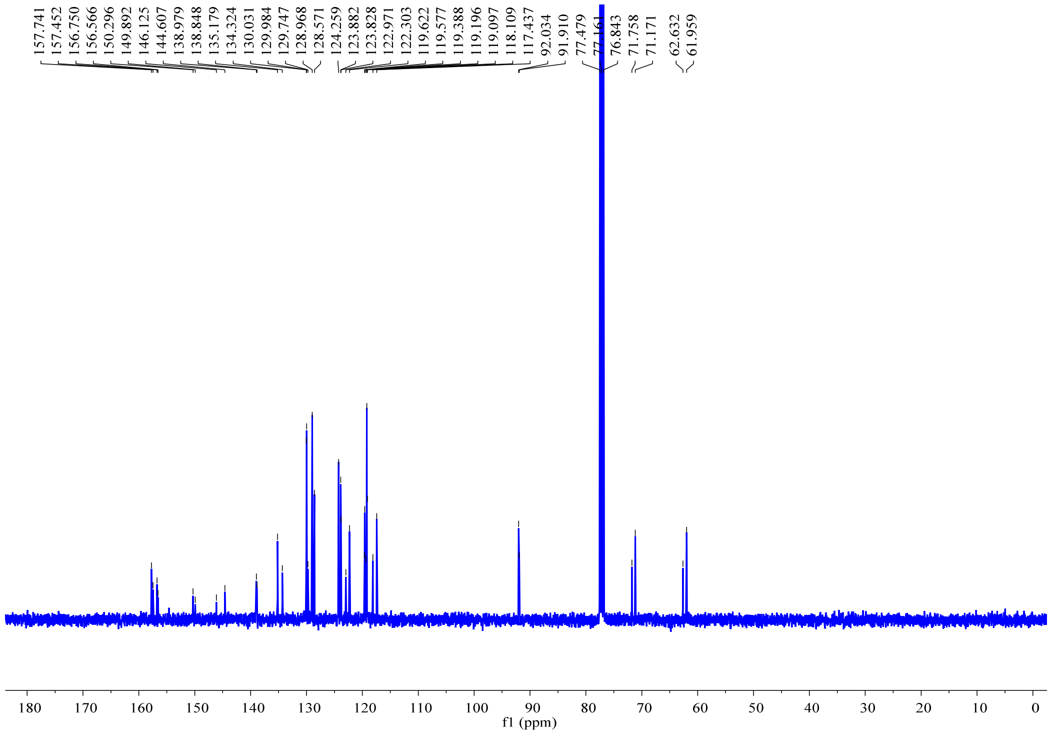
157.741
157.452
156.750
156.566
150.296
149.892
146.125
144.607
138.979
138.848
135.179
134.324
130.031
129.984
129.747
128.968
128.571
124.259
123.882
123.828
122.971
122.303
119.622
119.577
119.388
119.196
119.097
118.109
117.437
92.034
91.910
77.479
77.161
76.843
71.758
71.171
62.632
61.959
180 170 160 150 140 130 120 110 100 90 80 70 60 50 40 30 20 10 0
f1 (ppm)
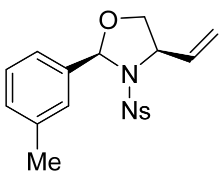
O
N
Ns
Me

3k

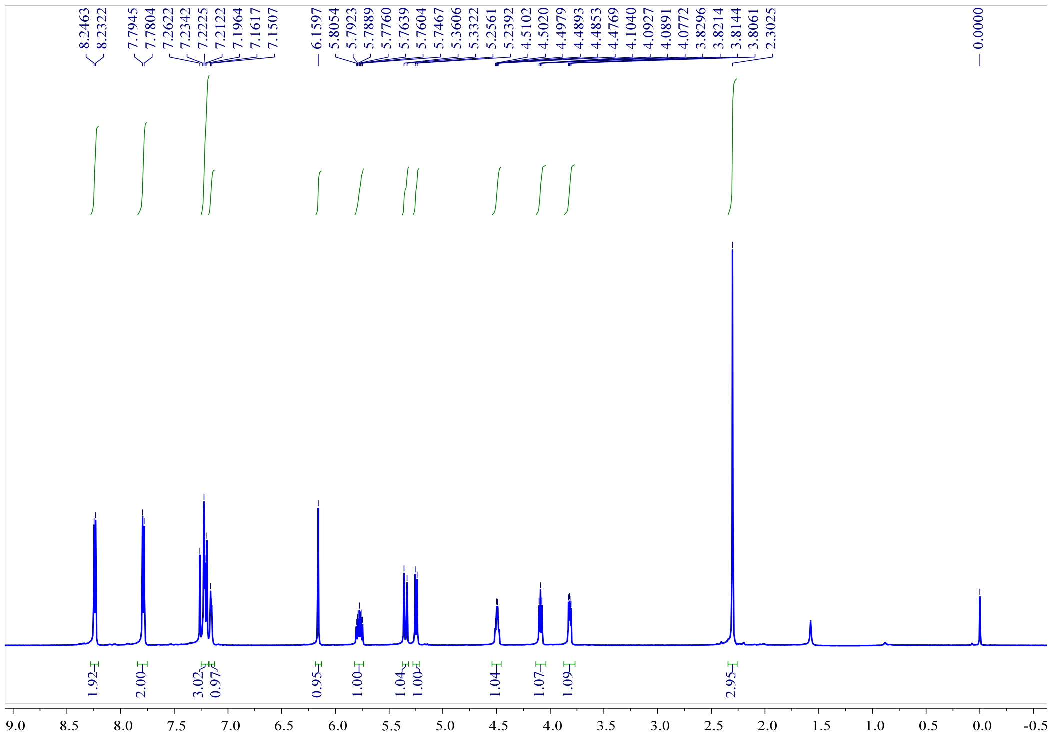

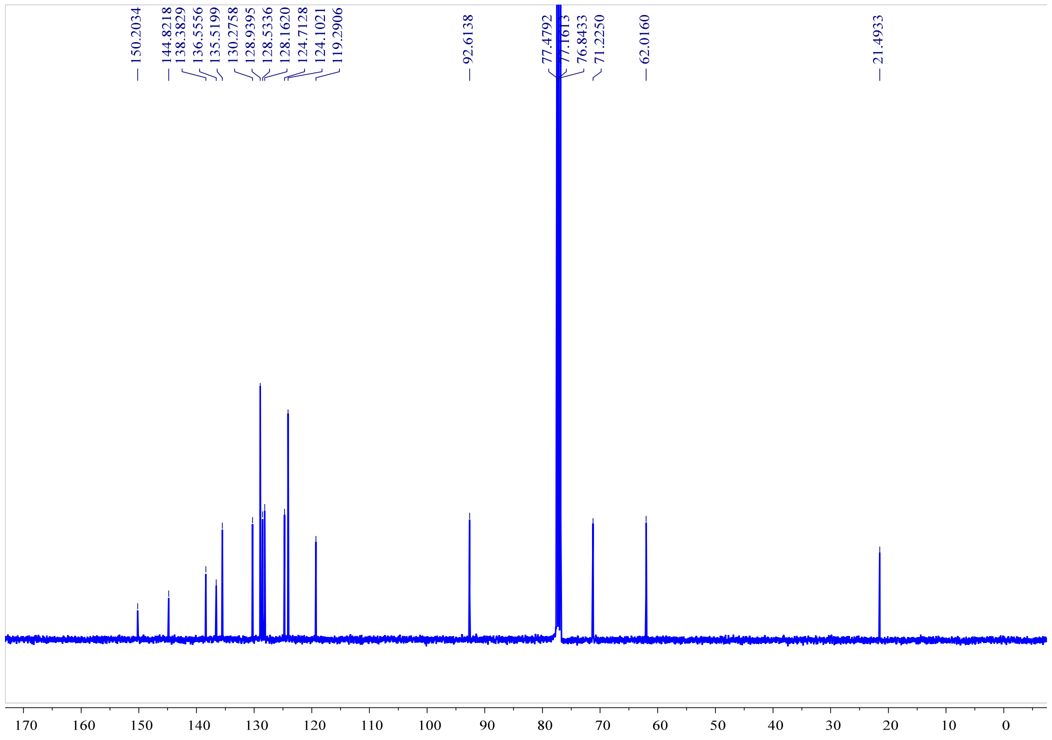

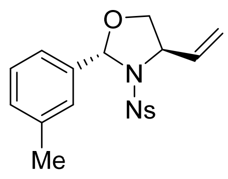

3k'

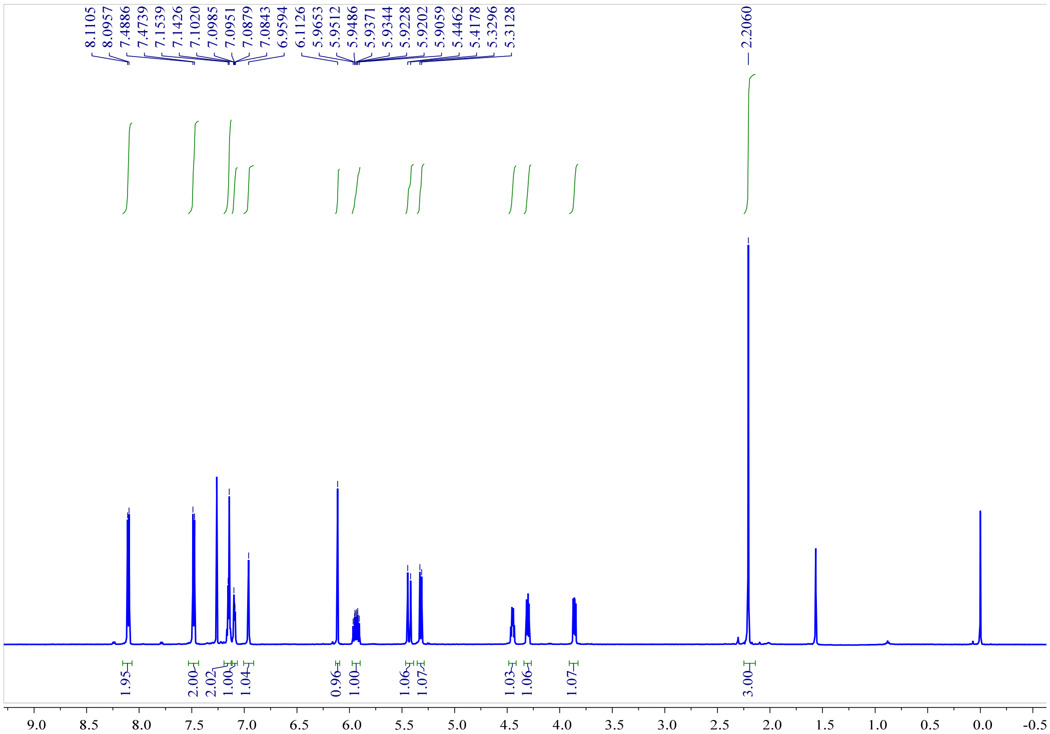
8.1105 8.0957 7.4886 7.4739 7.1539 7.1426 7.1020 7.0985 7.0951 7.0879 7.0843 6.9594 6.1126 5.9653 5.9512 5.9486 5.9371 5.9344 5.9228 5.9202 5.9059 5.4462 5.4178 5.3296 5.3128
2.2060
1.95 2.00 2.02 1.00 1.04 0.96 1.00 1.06 1.07 1.03 1.06 1.07 3.00
9.0 8.5 8.0 7.5 7.0 6.5 6.0 5.5 5.0 4.5 4.0 3.5 3.0 2.5 2.0 1.5 1.0 0.5 0.0 -0.5

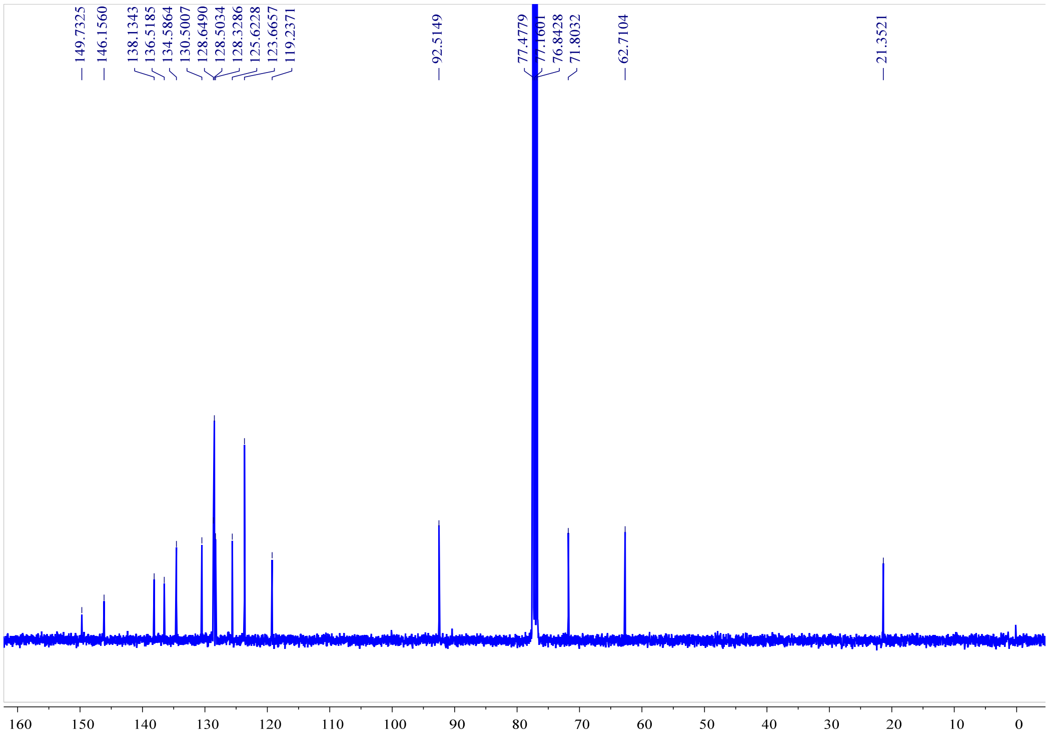
149.7325 146.1560 138.1343 136.5185 134.5864 130.5007 128.6490 128.5034 128.3286 125.6228 123.6657 119.2371
92.5149
77.4779 77.1601 76.8428 71.8032
62.7104
21.3521
160 150 140 130 120 110 100 90 80 70 60 50 40 30 20 10 0

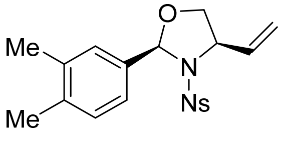
Me
Me
O
N
Ns

3l

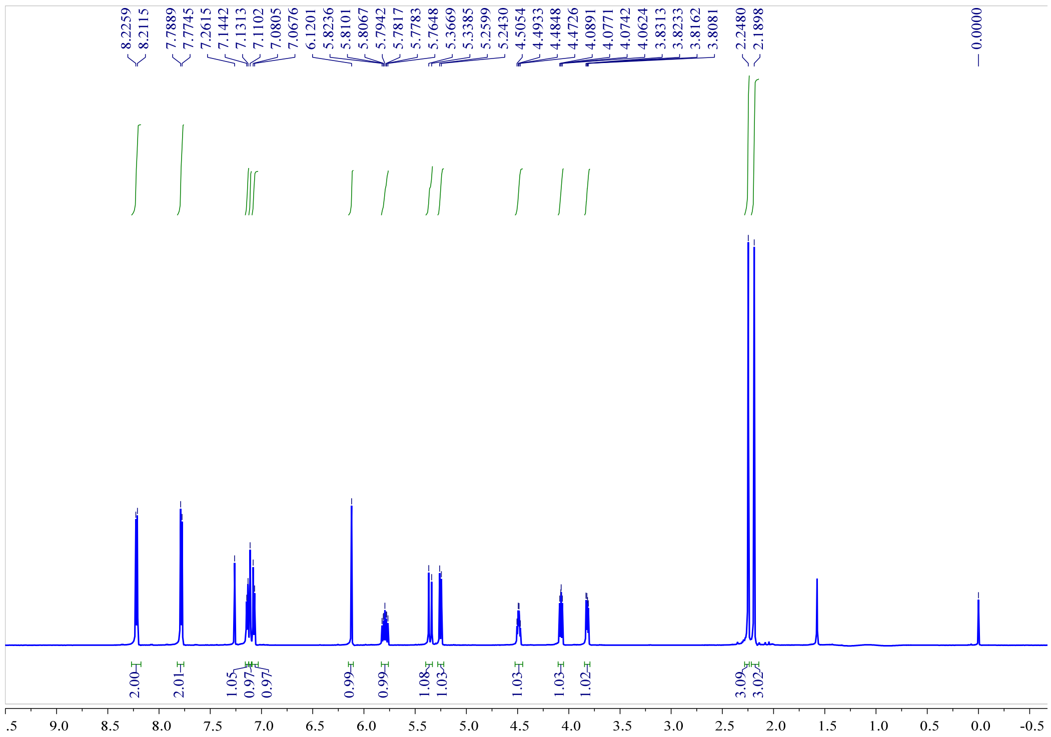
8.2259
8.2115
7.7889
7.7745
7.2615
7.1442
7.1313
7.1102
7.0805
7.0676
6.1201
5.8236
5.8101
5.8067
5.7942
5.7817
5.7783
5.7648
5.3669
5.3385
5.2599
5.2430
4.5054
4.4933
4.4848
4.4726
4.0891
4.0771
4.0742
4.0624
3.8313
3.8233
3.8162
3.8081
2.2480
2.1898
0.0000
2.00
2.01
1.05
0.97
0.97
0.99
0.99
1.08
1.03
1.03
1.03
1.02
3.09
3.02
.5 9.0 8.5 8.0 7.5 7.0 6.5 6.0 5.5 5.0 4.5 4.0 3.5 3.0 2.5 2.0 1.5 1.0 0.5 0.0 -0.5

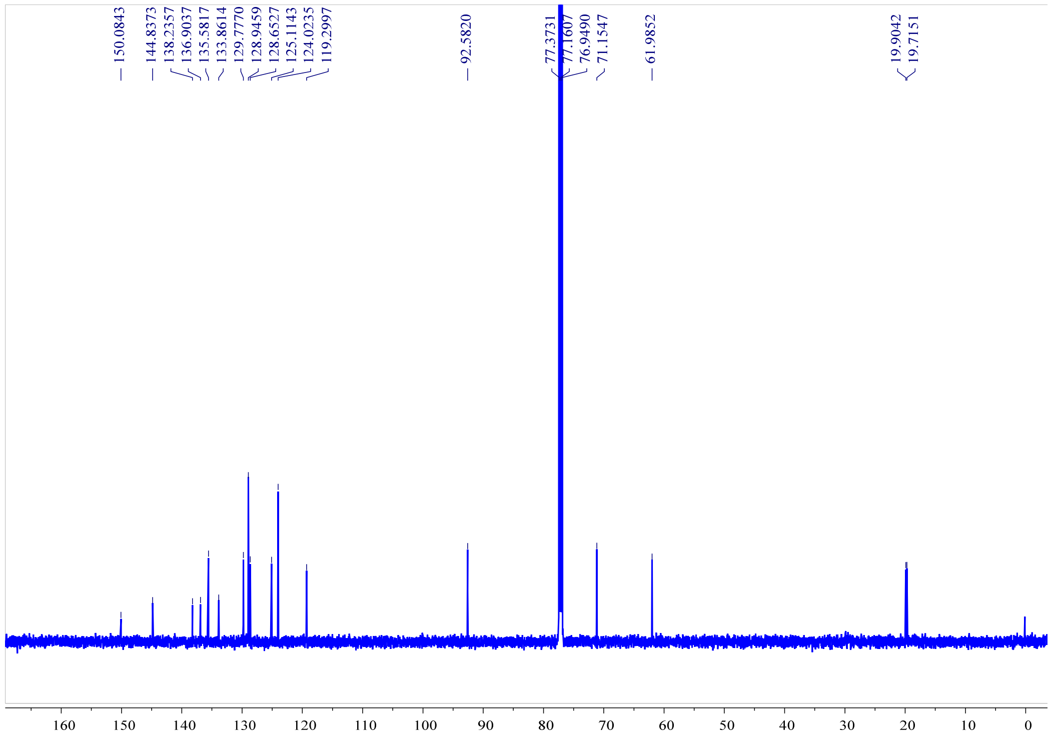
150.0843
144.8373
138.2357
136.9037
135.5817
133.8614
129.7770
128.9459
128.6527
125.1143
124.0235
119.2997
92.5820
77.3731
77.1607
76.9490
71.1547
61.9852
19.9042
19.7151
160 150 140 130 120 110 100 90 80 70 60 50 40 30 20 10 0

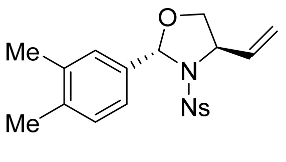
O
Me
N
Me
Ns

3l'

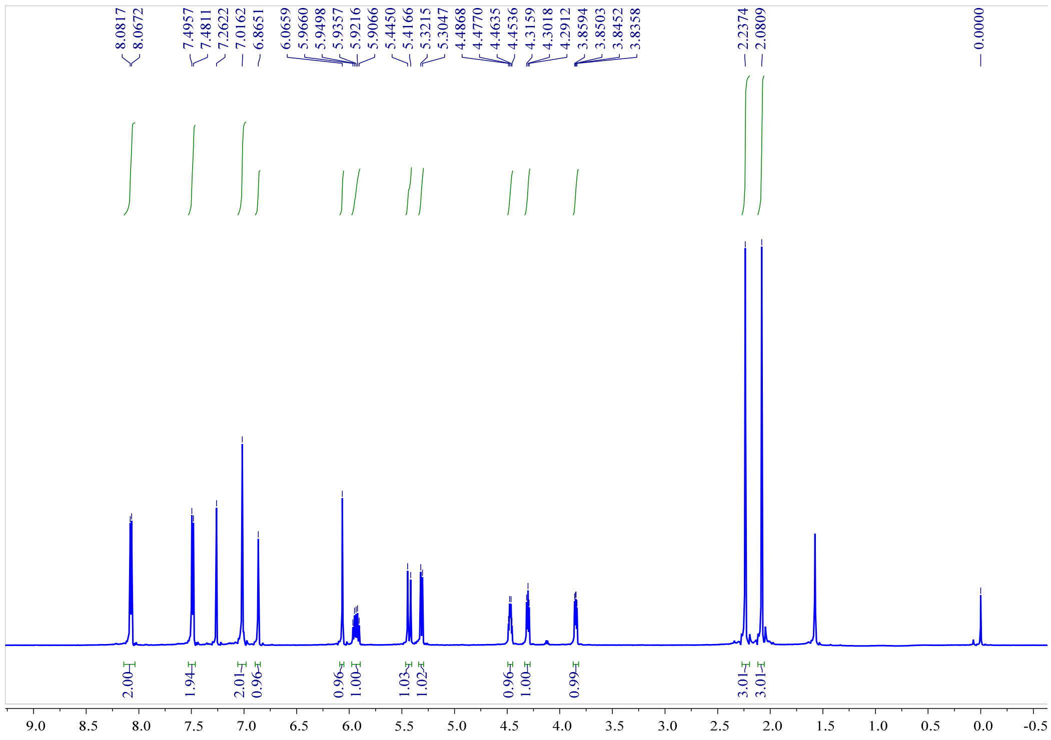
8.0817
8.0672
7.4957
7.4811
7.2622
7.0162
6.8651
6.0659
5.9660
5.9498
5.9357
5.9216
5.9066
5.4450
5.4166
5.3215
5.3047
4.4868
4.4770
4.4635
4.4536
4.3159
4.3018
4.2912
3.8594
3.8503
3.8452
3.8358
2.2374
2.0809
0.0000
2.00
1.94
2.01
0.96
0.96
1.00
1.03
1.02
0.96
1.00
0.99
3.01
3.01

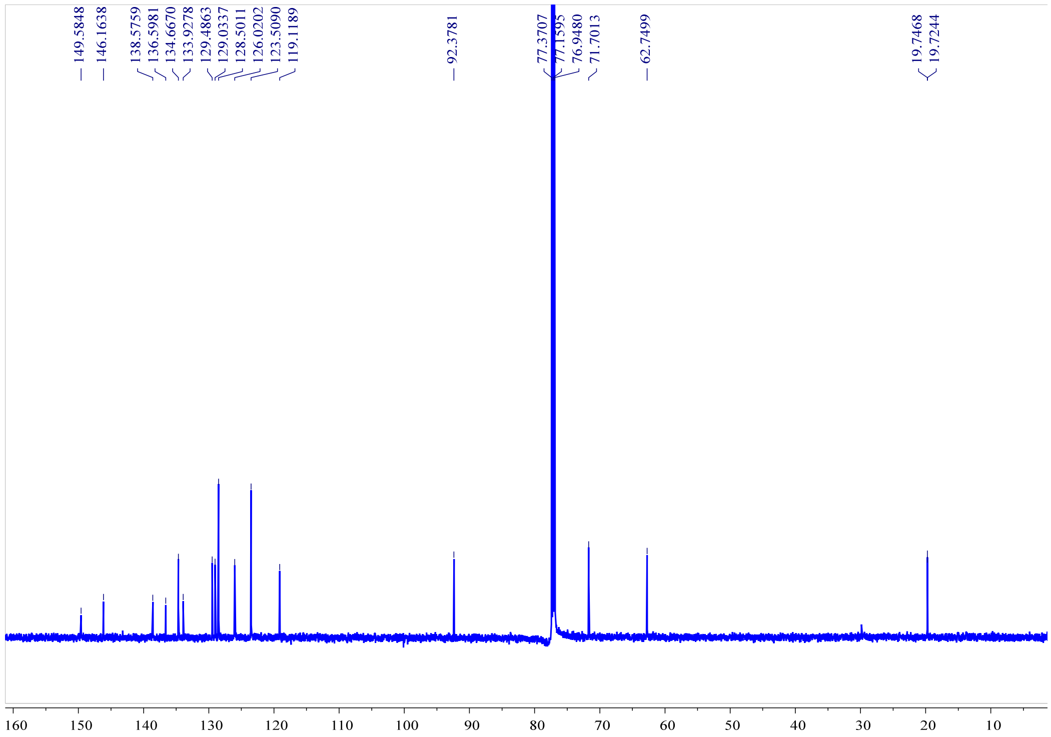
149.5848
146.1638
138.5759
136.5981
134.6670
133.9278
129.4863
129.0337
128.5011
126.0202
123.5090
119.1189
92.3781
77.3707
77.1595
76.9480
71.7013
62.7499
19.7468
19.7244

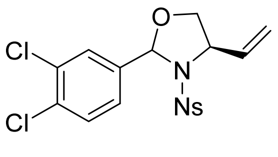
Cl
Cl
O
N
Ns

3m

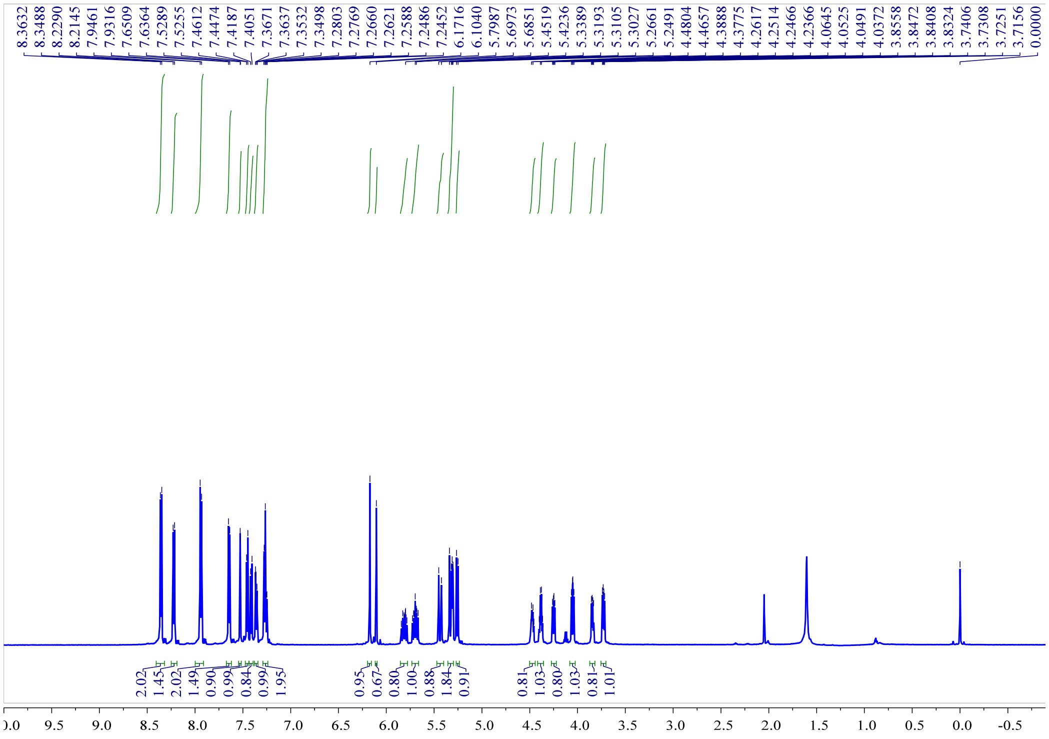
8.3632 8.3488 8.2290 8.2145 7.9461 7.9316 7.6509 7.6364 7.5289 7.5255 7.4612 7.4474 7.4187 7.4051 7.3671 7.3637 7.3532 7.3498 7.2803 7.2769 7.2660 7.2621 7.2588 7.2486 7.2452 6.1716 6.1040 5.7987 5.6973 5.6851 5.4519 5.4236 5.3389 5.3193 5.3105 5.3027 5.2661 5.2491 4.4804 4.4657 4.3888 4.3775 4.2617 4.2514 4.2466 4.2366 4.0645 4.0525 4.0491 4.0372 3.8558 3.8472 3.8408 3.8324 3.7406 3.7308 3.7251 3.7156 0.0000
2.02 1.45 2.02 1.49 0.90 0.99 0.84 0.99 1.95 0.95 0.67 0.80 1.00 0.88 1.84 0.91 0.81 1.03 0.80 1.03 0.81 1.01
9.5 9.0 8.5 8.0 7.5 7.0 6.5 6.0 5.5 5.0 4.5 4.0 3.5 3.0 2.5 2.0 1.5 1.0 0.5 0.0 -0.5

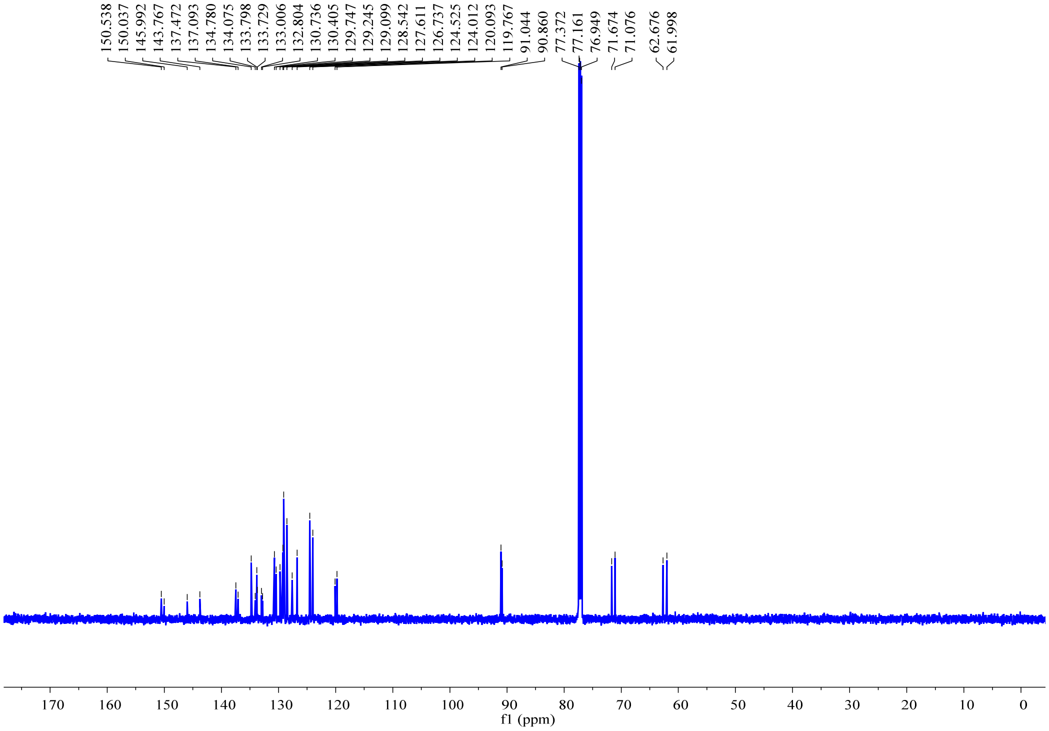
150.538 150.037 145.992 143.767 137.472 137.093 134.780 134.075 133.798 133.729 133.006 132.804 130.736 130.405 129.747 129.245 129.099 128.542 127.611 126.737 124.525 124.012 120.093 119.767 91.044 90.860 77.372 77.161 76.949 71.674 71.076 62.676 61.998
170 160 150 140 130 120 110 100 90 80 70 60 50 40 30 20 10 0
f1 (ppm)

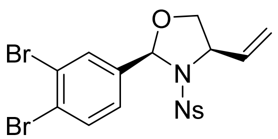
O
N
Ns
Br
Br

3m

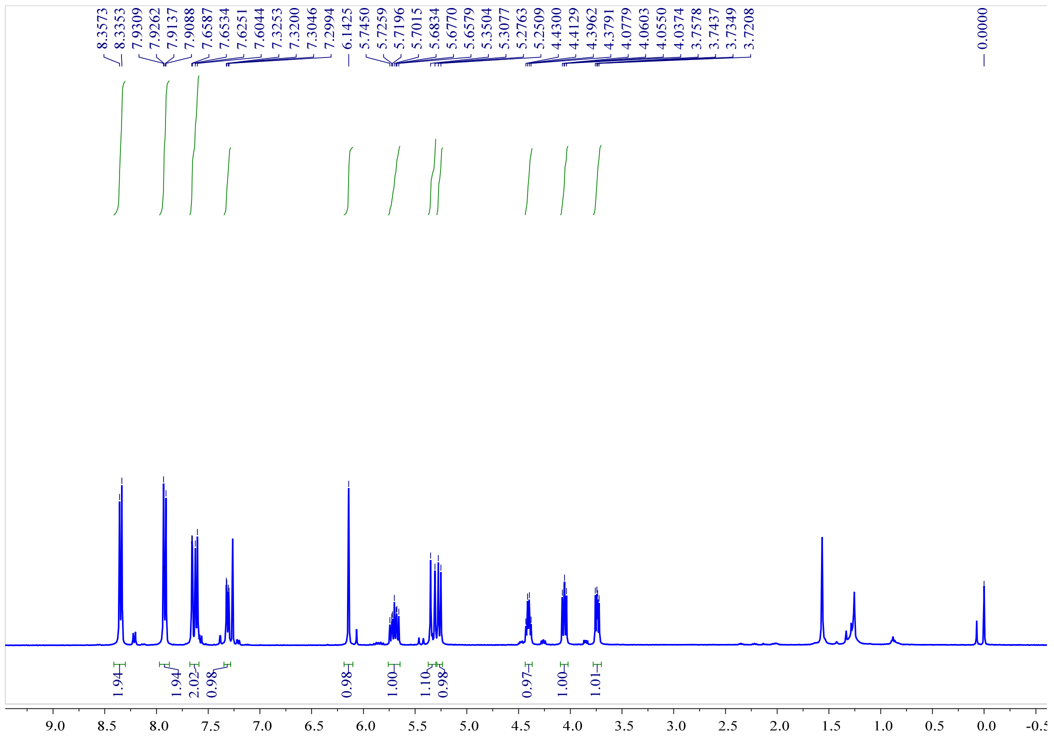
8.3573
8.3353
7.9309
7.9262
7.9137
7.9088
7.6587
7.6534
7.6251
7.6044
7.3253
7.3200
7.3046
7.2994
6.1425
5.7450
5.7259
5.7196
5.7015
5.6834
5.6770
5.6579
5.3504
5.3077
5.2763
5.2509
4.4300
4.4129
4.3962
4.3791
4.0779
4.0603
4.0550
4.0374
3.7578
3.7437
3.7349
3.7208
0.0000
1.94
1.94
2.02
0.98
0.98
1.00
1.10
0.98
0.97
1.00
1.01
9.0
8.5
8.0
7.5
7.0
6.5
6.0
5.5
5.0
4.5
4.0
3.5
3.0
2.5
2.0
1.5
1.0
0.5
0.0

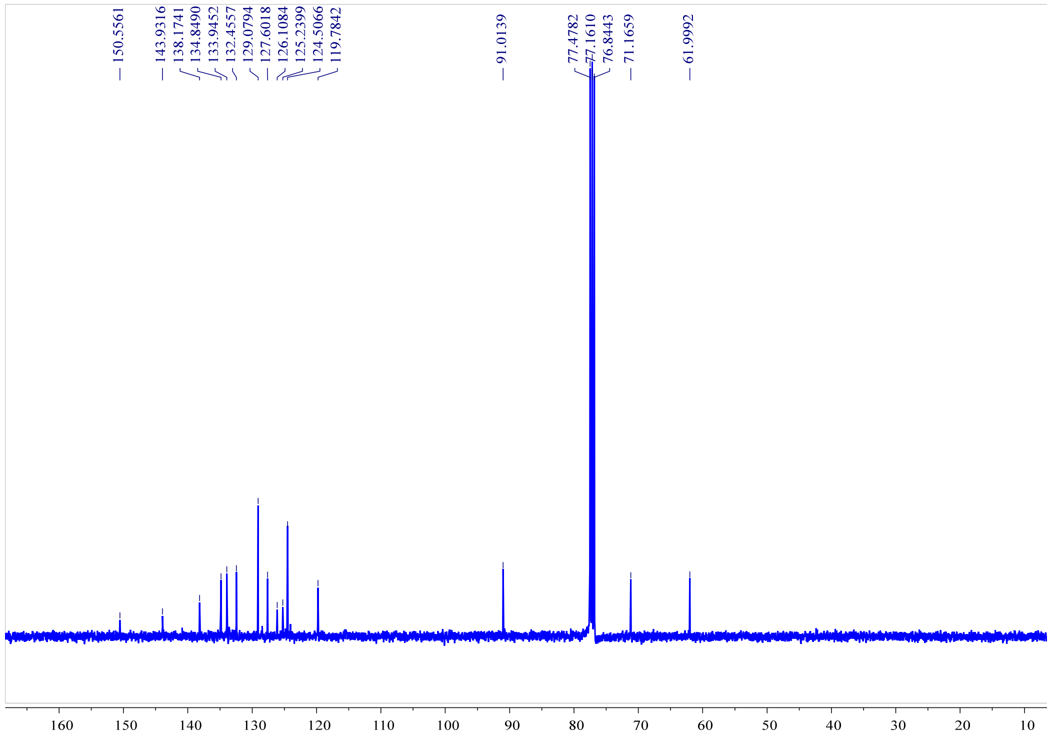
150.5561
143.9316
138.1741
134.8490
133.9452
132.4557
129.0794
127.6018
126.1084
125.2399
124.5066
119.7842
91.0139
77.4782
77.1610
76.8443
71.1659
61.9992
160
150
140
130
120
110
100
90
80
70
60
50
40
30
20
10

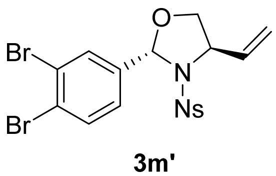
Br
Br
O
N
Ns

3m'

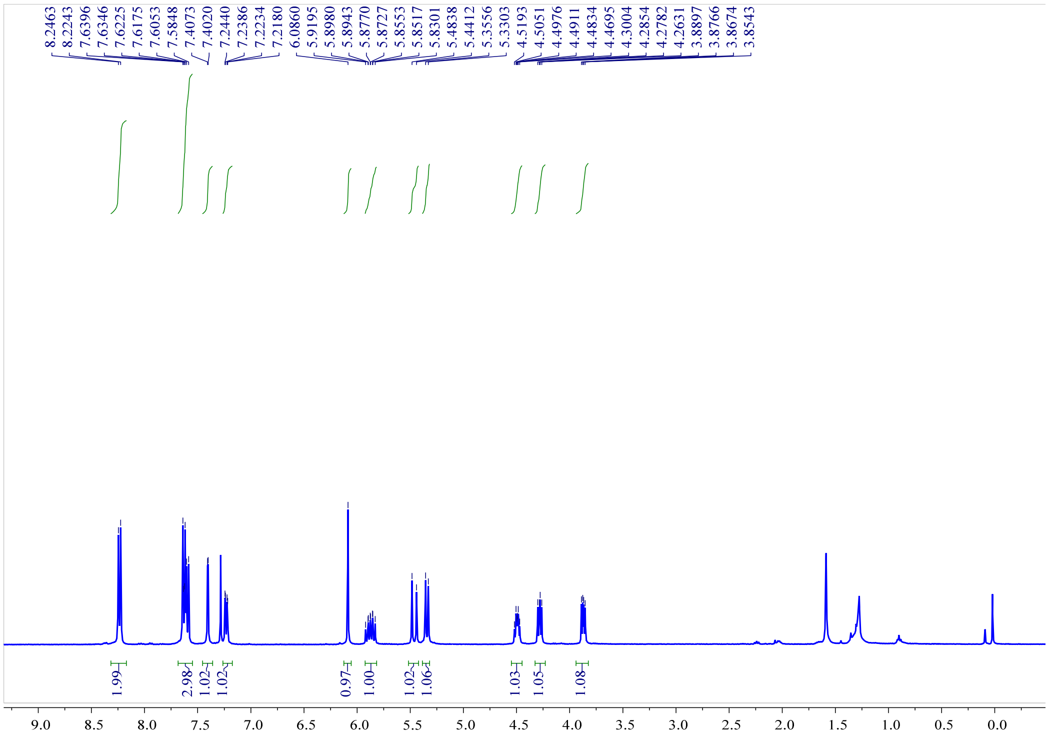

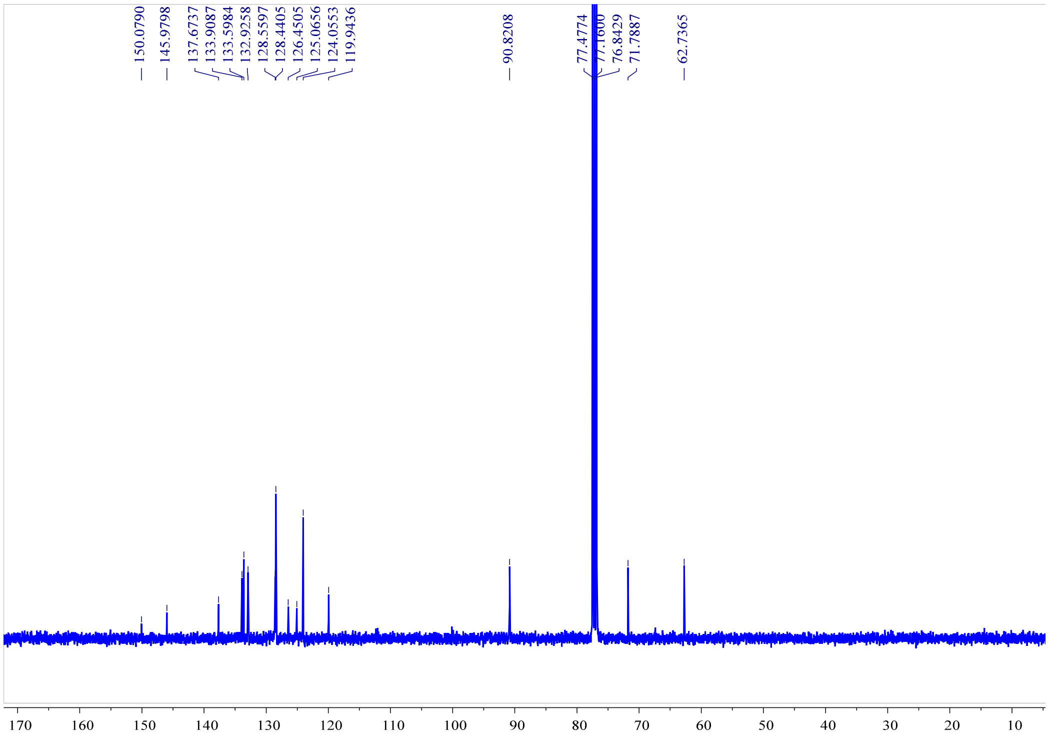

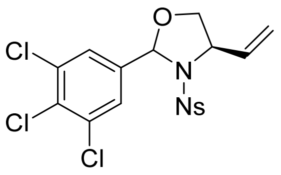

3n

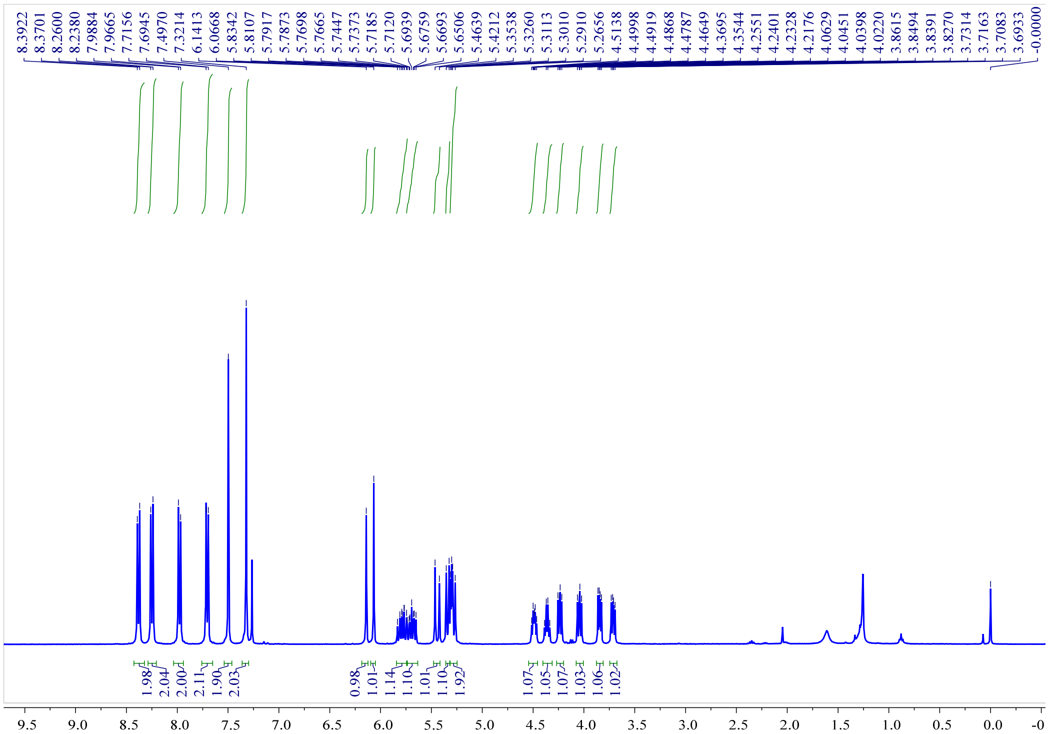
8.3922
8.3701
8.2600
8.2380
7.9884
7.9665
7.7156
7.6945
7.4970
7.3214
6.1413
6.0668
5.8342
5.8107
5.7917
5.7873
5.7698
5.7665
5.7447
5.7373
5.7185
5.7120
5.6939
5.6759
5.6693
5.6506
5.4639
5.4212
5.3538
5.3260
5.3113
5.3010
5.2910
5.2656
4.5138
4.4998
4.4919
4.4868
4.4787
4.4649
4.3695
4.3544
4.2551
4.2401
4.2328
4.2176
4.0629
4.0451
4.0398
4.0220
3.8615
3.8494
3.8391
3.8270
3.7314
3.7163
3.7083
3.6933
-0.0000
1.98
2.04
2.00
2.11
1.90
2.03
0.98
1.01
1.14
1.10
1.01
1.10
1.92
1.07
1.05
1.07
1.03
1.06
1.02
9.5 9.0 8.5 8.0 7.5 7.0 6.5 6.0 5.5 5.0 4.5 4.0 3.5 3.0 2.5 2.0 1.5 1.0 0.5 0.0 -0

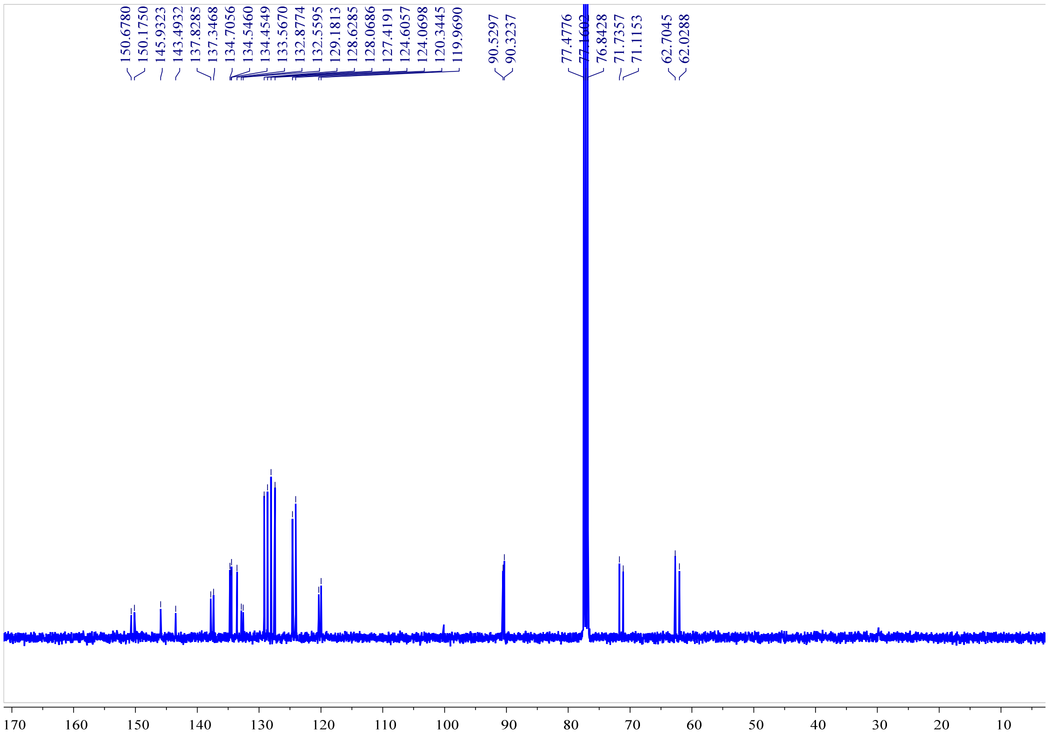
150.6780
150.1750
145.9323
143.4932
137.8285
137.3468
134.7056
134.5460
134.4549
133.5670
132.8774
132.5595
129.1813
128.6285
128.0686
127.4191
124.6057
124.0698
120.3445
119.9690
90.5297
90.3237
77.4776
76.8428
71.7357
71.1153
62.7045
62.0288
170 160 150 140 130 120 110 100 90 80 70 60 50 40 30 20 10

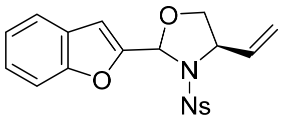
O
N
Ns
O

3o

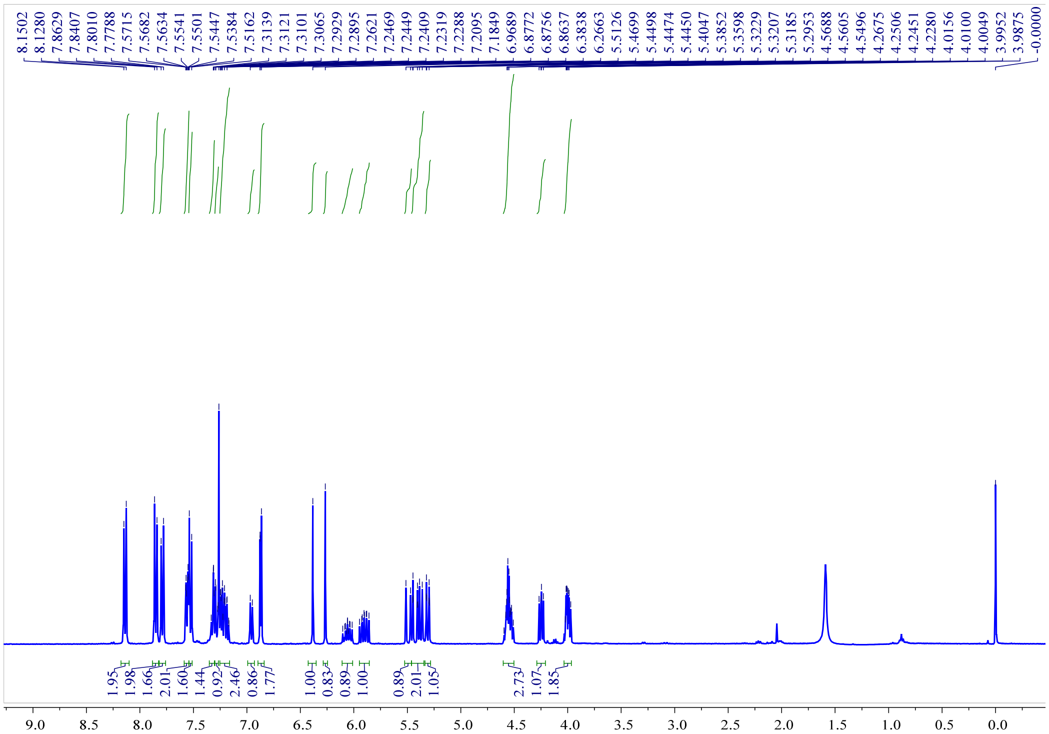
8.1502
8.1280
7.8629
7.8407
7.8010
7.7788
7.5715
7.5682
7.5634
7.5541
7.5501
7.5447
7.5384
7.5162
7.3139
7.3121
7.3101
7.3065
7.2929
7.2895
7.2621
7.2469
7.2449
7.2409
7.2319
7.2288
7.2095
7.1849
6.9689
6.8772
6.8756
6.8637
6.3838
6.2663
5.5126
5.4699
5.4498
5.4474
5.4450
5.4047
5.3852
5.3598
5.3229
5.3207
5.3185
5.2953
4.5688
4.5605
4.5496
4.2675
4.2506
4.2451
4.2280
4.0156
4.0100
4.0049
3.9952
3.9875
-0.0000
1.95
1.98
1.66
2.01
1.60
1.44
0.92
2.46
0.86
1.77
1.00
0.83
0.89
1.00
0.89
2.01
1.05
2.73
1.07
1.85
9.0 8.5 8.0 7.5 7.0 6.5 6.0 5.5 5.0 4.5 4.0 3.5 3.0 2.5 2.0 1.5 1.0 0.5 0.0

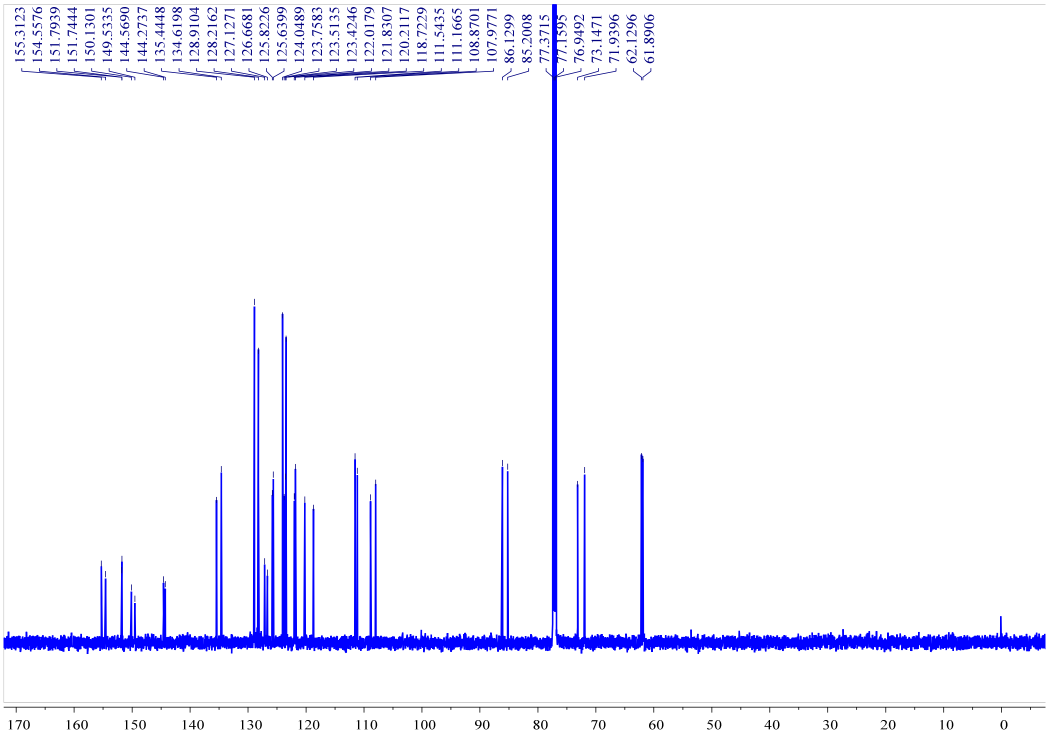
155.3123
154.5576
151.7939
151.7444
150.1301
149.5335
144.5690
144.2737
135.4448
134.6198
128.9104
128.2162
127.1271
126.6681
125.8226
125.6399
124.0489
123.7583
123.5135
123.4246
122.0179
121.8307
120.2117
118.7229
111.5435
111.1665
108.8701
107.9771
86.1299
85.2008
77.3715
77.1595
76.9492
73.1471
71.9396
62.1296
61.8906
170 160 150 140 130 120 110 100 90 80 70 60 50 40 30 20 10 0

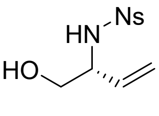
HN
Ns
HO

4

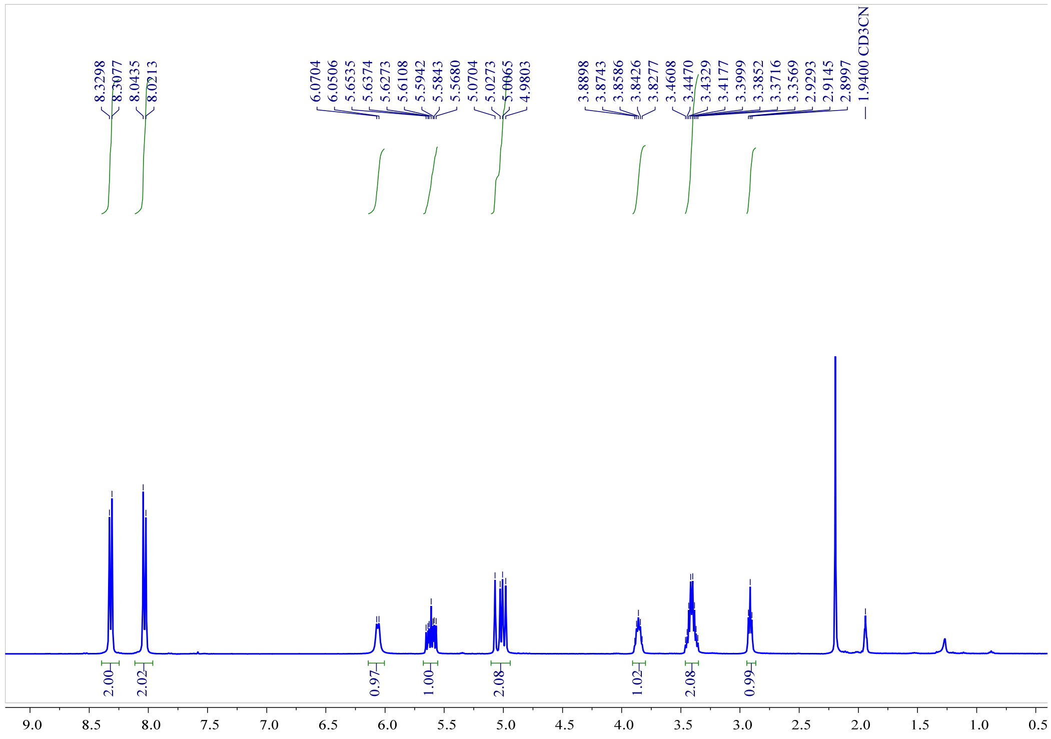
8.3298
8.3077
8.0435
8.0213
6.0704
6.0506
5.6535
5.6374
5.6273
5.6108
5.5942
5.5843
5.5680
5.0704
5.0273
5.0065
4.9803
3.8898
3.8743
3.8586
3.8426
3.8277
3.4608
3.4470
3.4329
3.4177
3.3999
3.3852
3.3716
3.3569
2.9293
2.9145
2.8997
1.9400 CD3CN
2.00
2.02
0.97
1.00
2.08
1.02
2.08
0.99
9.0 8.5 8.0 7.5 7.0 6.5 6.0 5.5 5.0 4.5 4.0 3.5 3.0 2.5 2.0 1.5 1.0 0.5

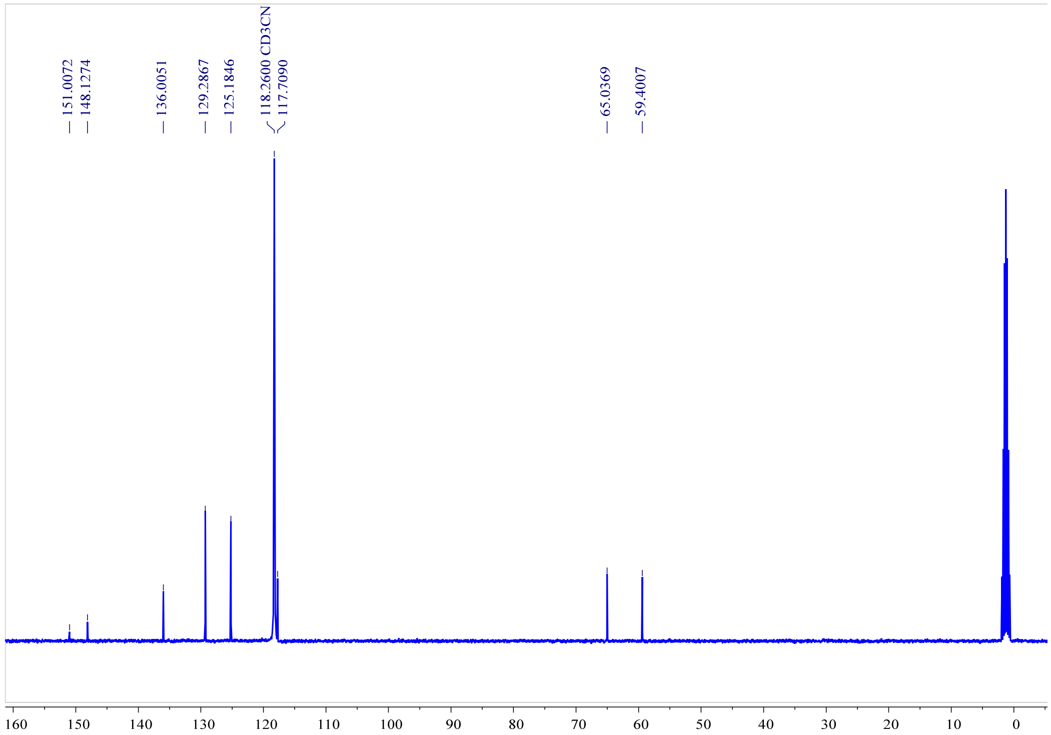
151.0072
148.1274
136.0051
129.2867
125.1846
118.2600 CD3CN
117.7090
65.0369
59.4007
160 150 140 130 120 110 100 90 80 70 60 50 40 30 20 10 0

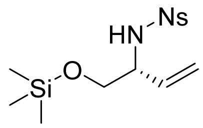
HN
Ns
O
Si

5

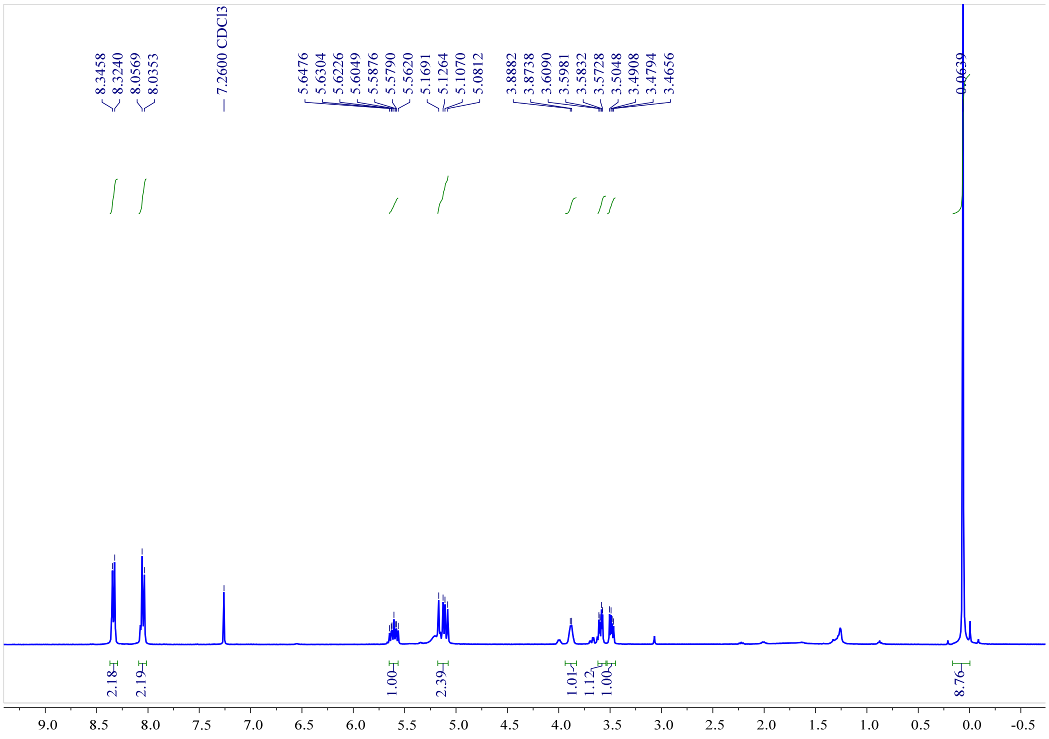

8.3458
8.3240
8.0569
8.0353
7.2600 CDCl3
5.6476
5.6304
5.6226
5.6049
5.5876
5.5790
5.5620
5.1691
5.1264
5.1070
5.0812
3.8882
3.8738
3.6090
3.5981
3.5832
3.5728
3.5048
3.4908
3.4794
3.4656
0.0639
2.18
2.19
1.00
2.39
1.01
1.12
1.00
8.76
9.0 8.5 8.0 7.5 7.0 6.5 6.0 5.5 5.0 4.5 4.0 3.5 3.0 2.5 2.0 1.5 1.0 0.5 0.0 -0.5

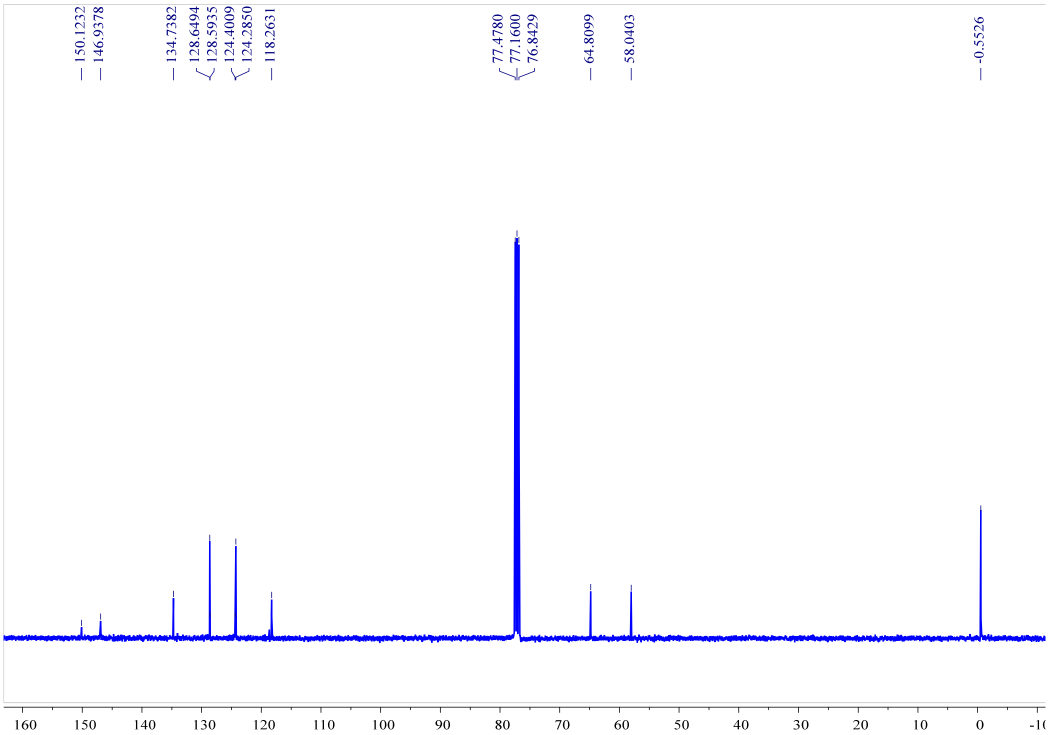

150.1232
146.9378
134.7382
128.6494
128.5935
124.4009
124.2850
118.2631
77.4780
77.1600
76.8429
64.8099
58.0403
-0.5526
160 150 140 130 120 110 100 90 80 70 60 50 40 30 20 10 0

8. HPLC Spectra of compounds 3 / 3′, 4 and 5

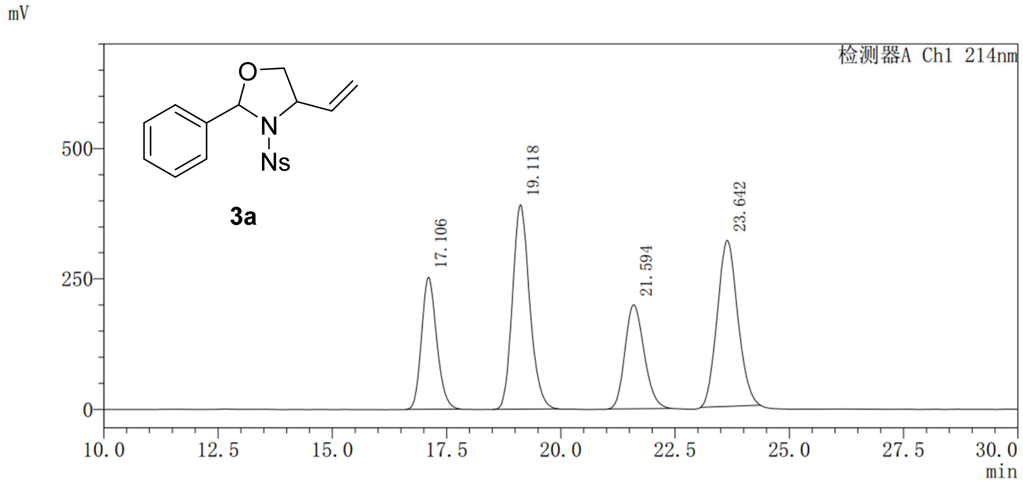

Peak No.	R. Time (min)	Peak Area (mV*min)	Percent (%)
1	17.106	5836701	18.343
2	19.118	10267306	32.267
3	21.594	5807382	18.251
4	23.642	9908455	31.139
**Total**		31819844	100.000

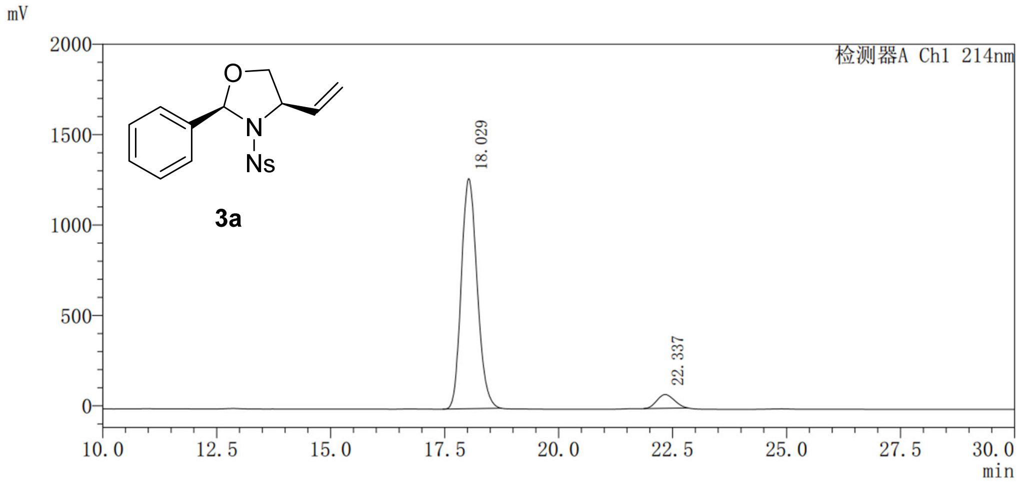

Peak No.	R. Time (min)	Peak Area (mV*min)	Percent (%)
1	18.029	30932429	93.854
2	22.337	2025483	6.146
**Total**		32957912	100.000

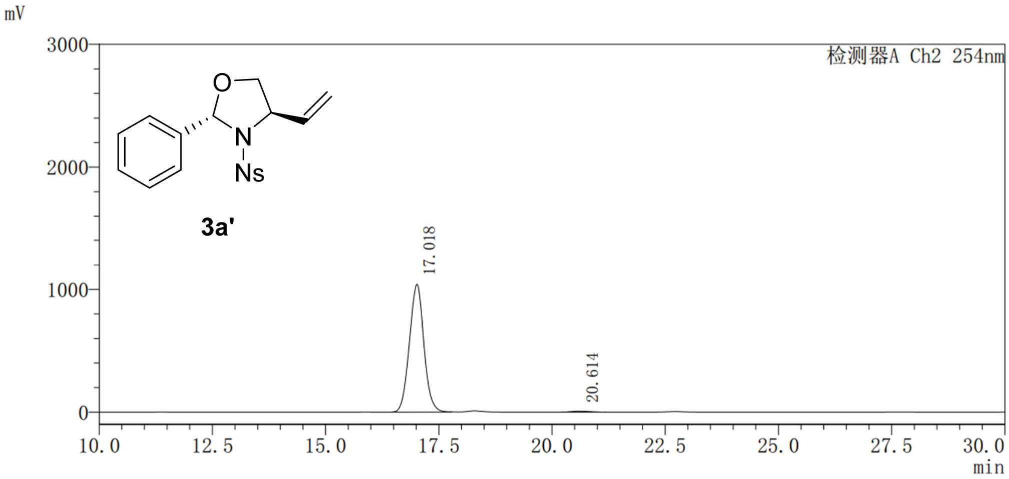

Peak No.	R. Time (min)	Peak Area (mV*min)	Percent (%)
1	17.016	42570141	99.232
2	20.610	329296	0.768
**Total**		42899437	100.000

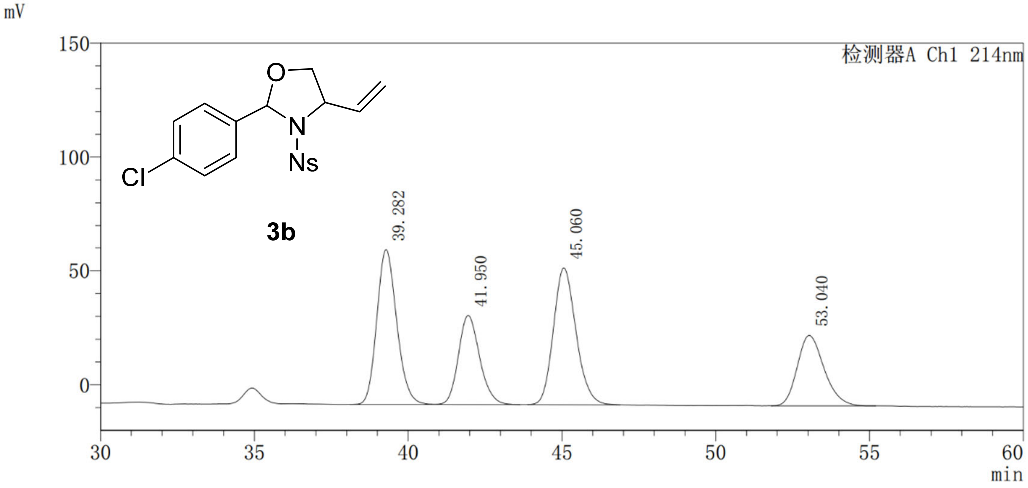

Peak No.	R. Time (min)	Peak Area (mV*min)	Percent (%)
1	39.282	3056421	30.884
2	41.950	1881503	19.012
3	45.060	3075031	31.072
4	53.040	1883632	19.033
**Total**		9896586	100.000

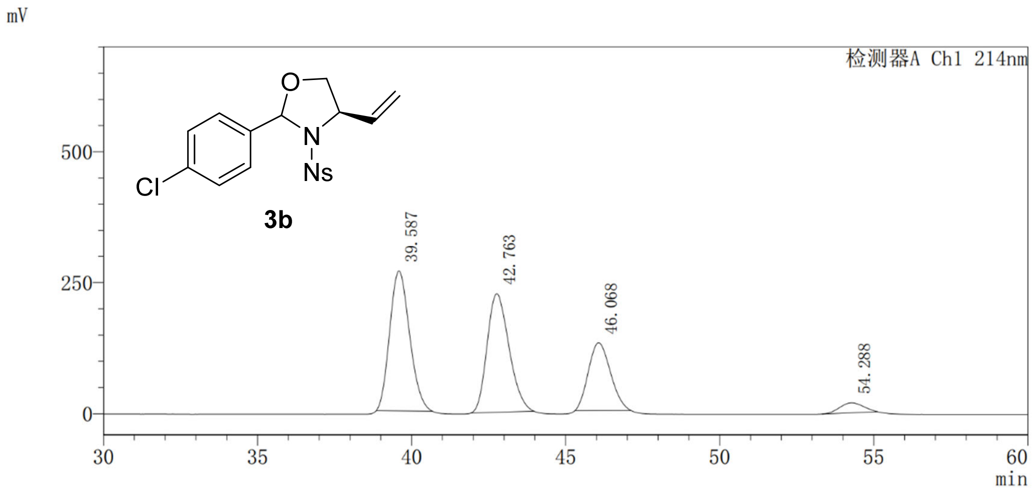

Peak No.	R. Time (min)	Peak Area (mV*min)	Percent (%)
1	39.587	11954001	39.080
2	42.763	11164872	36.500
3	46.068	6459749	21.118
4	54.288	1009853	3.301
**Total**		30588474	100.000

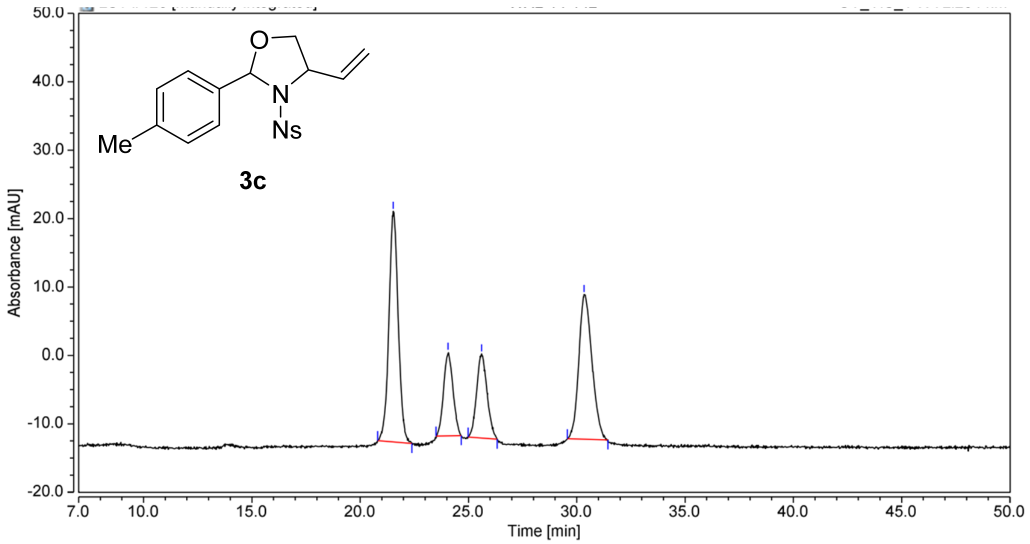

Peak No.	R. Time (min)	Peak Area (mAU*min)	Percent (%)
1	21.524	16.755	36.60
2	24.057	6.130	13.75
3	25.607	6.848	15.37
4	30.340	14.834	34.29
**Total**		44.567	100.000

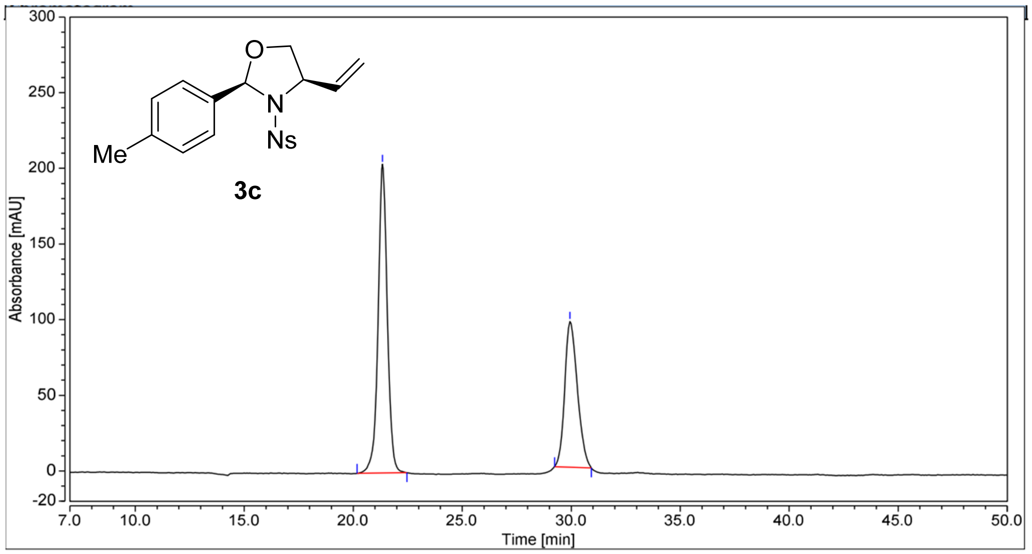

Peak No.	R. Time (min)	Peak Area (mAU*min)	Percent (%)
1	21.337	100.227	60.74
2	29.945	64.786	39.26
**Total**		165.013	100

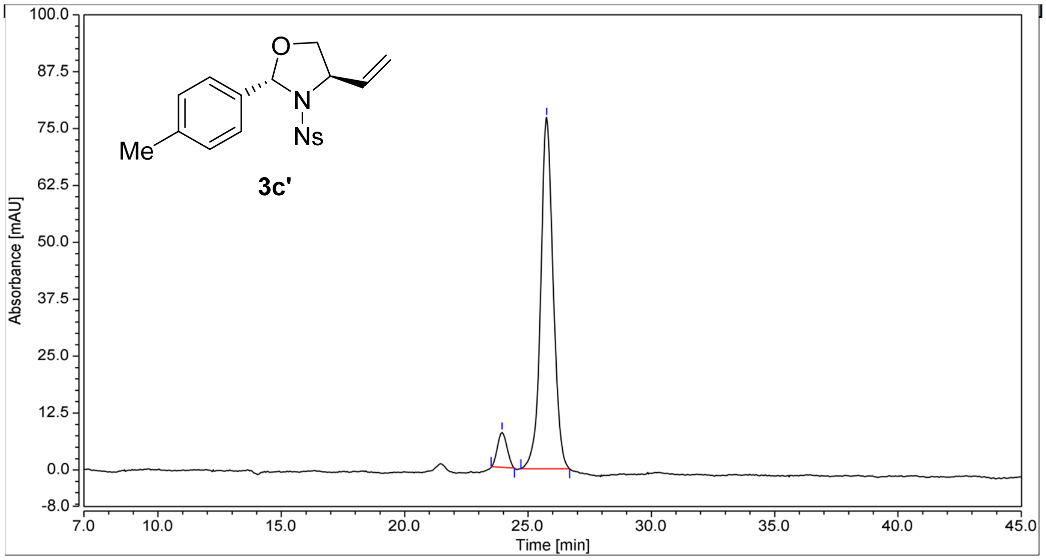

Peak No.	R. Time (min)	Peak Area (mAU*min)	Percent (%)
1	23.945	3.478	9.20
2	25.753	45.127	90.80
**Total**		48.605	100

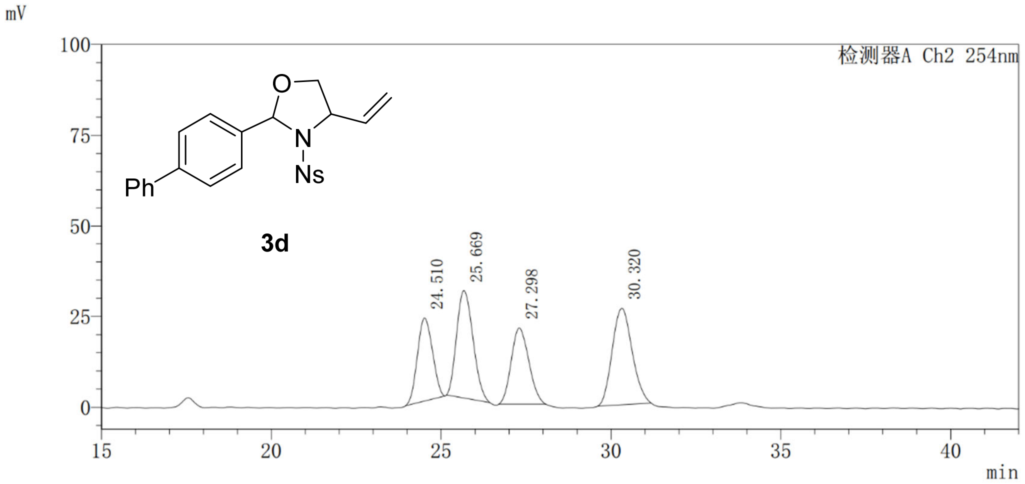

Peak No.	R. Time (min)	Peak Area (mV*min)	Percent (%)
1	24.510	704199	19.900
2	25.669	994826	28.112
3	27.298	755426	21.347
4	30.320	1084290	30.641
**Total**		3538742	100.000

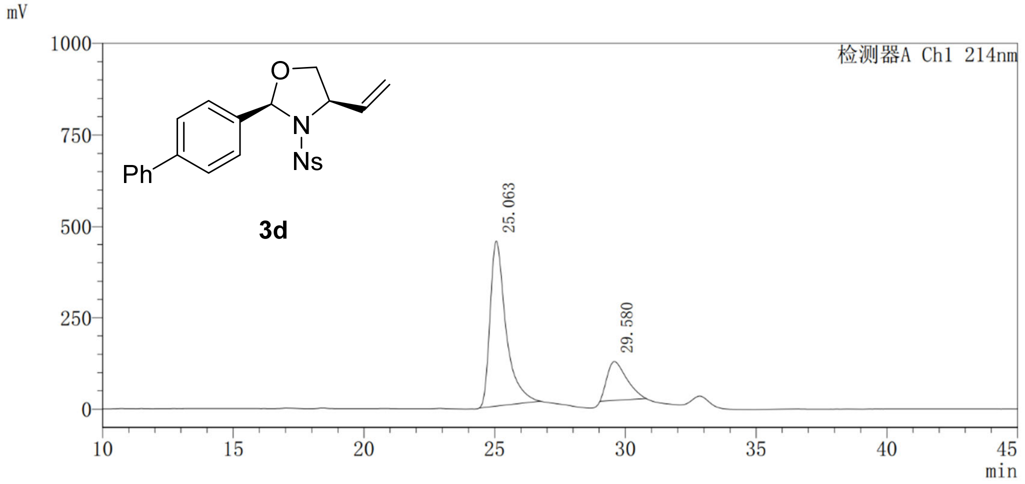

Peak No.	R. Time (min)	Peak Area (mV*min)	Percent (%)
1	25.063	19100735	77.582
2	29.580	5519236	22.418
**Total**		24619971	100.000

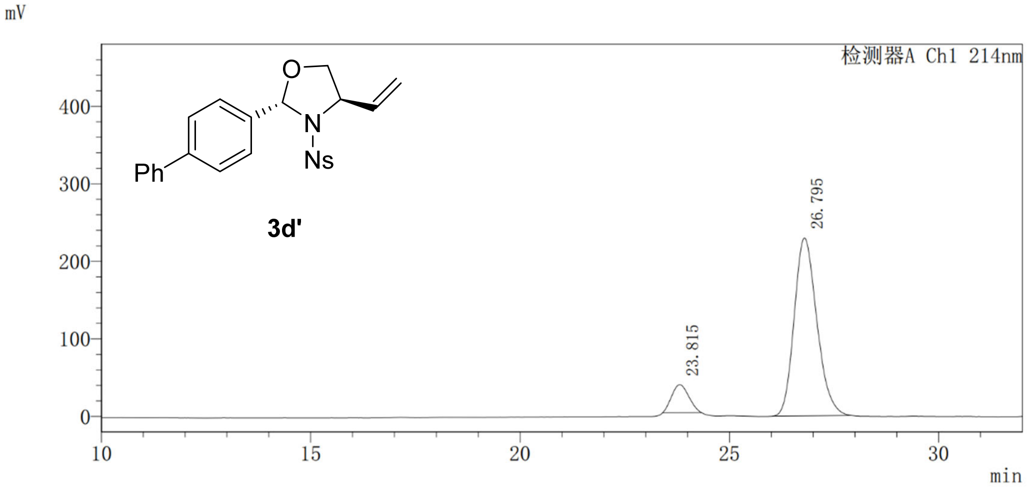

Peak No.	R. Time (min)	Peak Area (mV*min)	Percent (%)
1	23.815	975158	9.981
2	26.795	8794048	90.018
Total		9769206	100.000

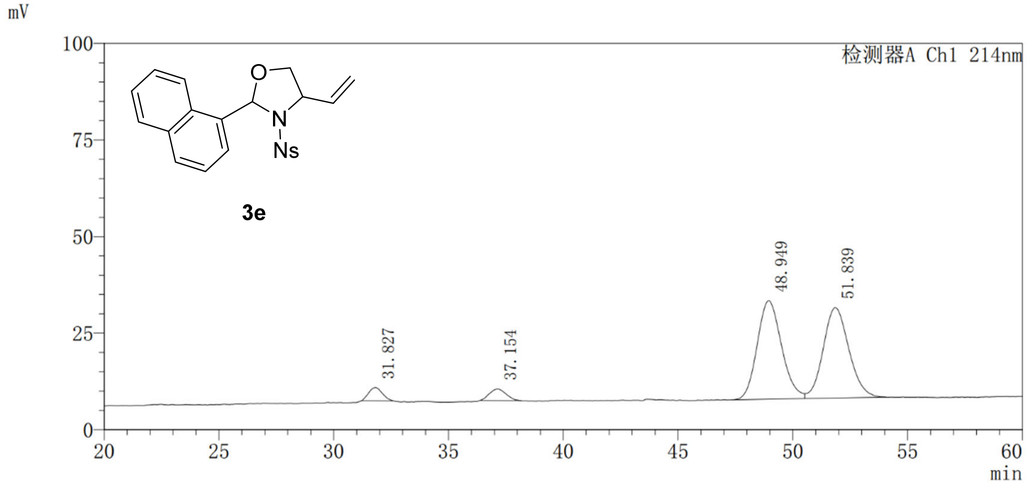

Peak No.	R. Time (min)	Peak Area (mV*min)	Percent (%)
1	31.827	142559	3.561
2	37.154	150352	3.756
3	48.949	1884274	47.074
4	51.839	1825607	45.608
Total		4002792	100.000

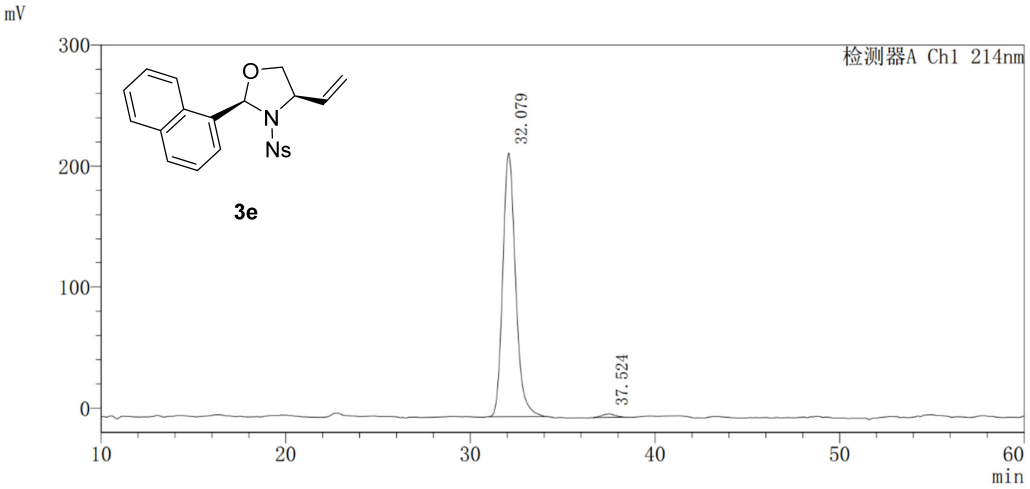

Peak No.	R. Time (min)	Peak Area (mV*min)	Percent (%)
1	32.079	10530438	98.747
2	37.524	133640	1.253
**Total**		10664077	100.000

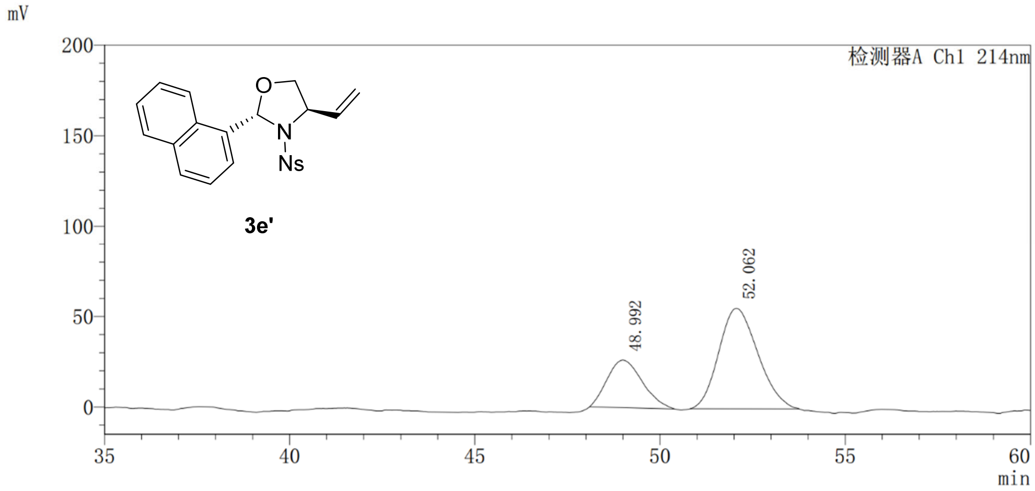

Peak No.	R. Time (min)	Peak Area (mV*min)	Percent (%)
1	48.992	1731676	29.833
2	52.062	4072936	70.167
**Total**		5804612	100.000

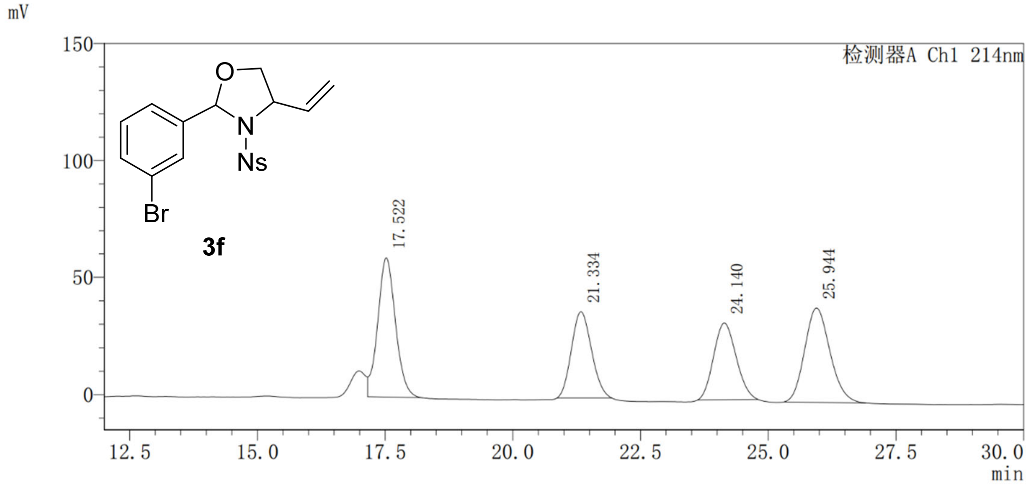

Peak No.	R. Time (min)	Peak Area (mV*min)	Percent (%)
1	**17.522**	1435080	29.716
2	21.334	1004127	20.792
3	24.140	1003009	20.769
4	25.944	1387090	28.722
**Total**		4829306	100.000

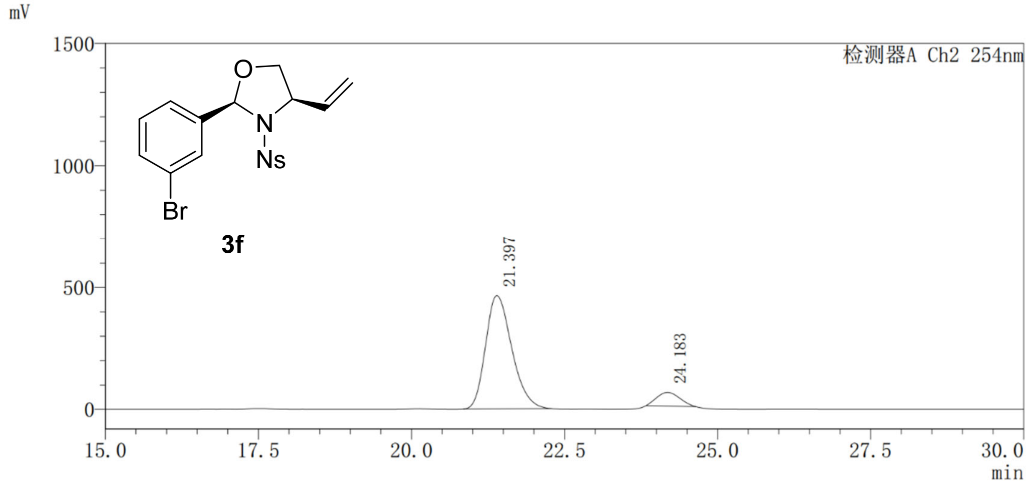

Peak No.	R. Time (min)	Peak Area (mV*min)	Percent (%)
1	21.397	13839602	90.482
2	24.183	1455838	9.518
**Total**		15295440	100.000

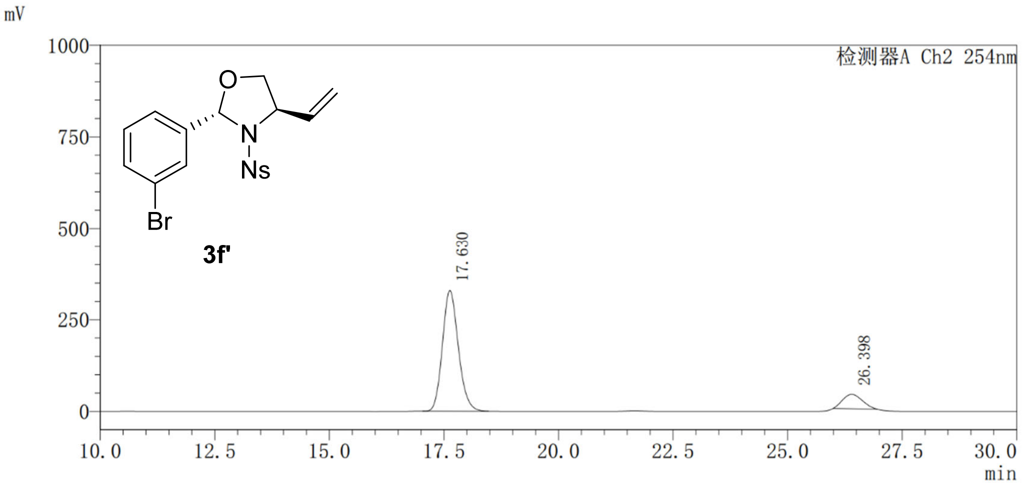

Peak No.	R. Time (min)	Peak Area (mV*min)	Percent (%)
1	17.630	7960221	87.138
2	26.398	1174972	12.862
**Total**		9135193	100.000

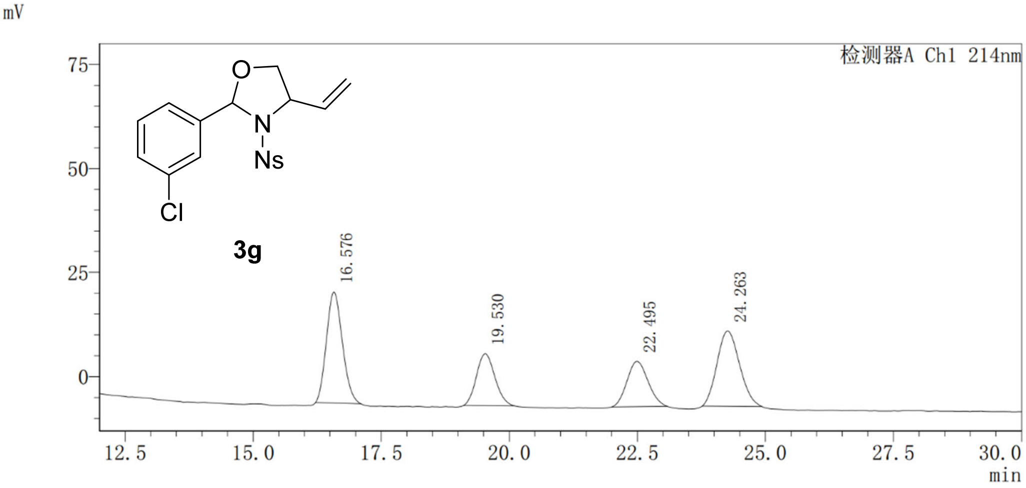

Peak No.	R. Time (min)	Peak Area (mV*min)	Percent (%)
1	16.576	576484	32.894
2	19.530	312817	17.849
3	22.495	310431	17.713
4	24.263	552821	31.544
**Total**		1752554	100.000

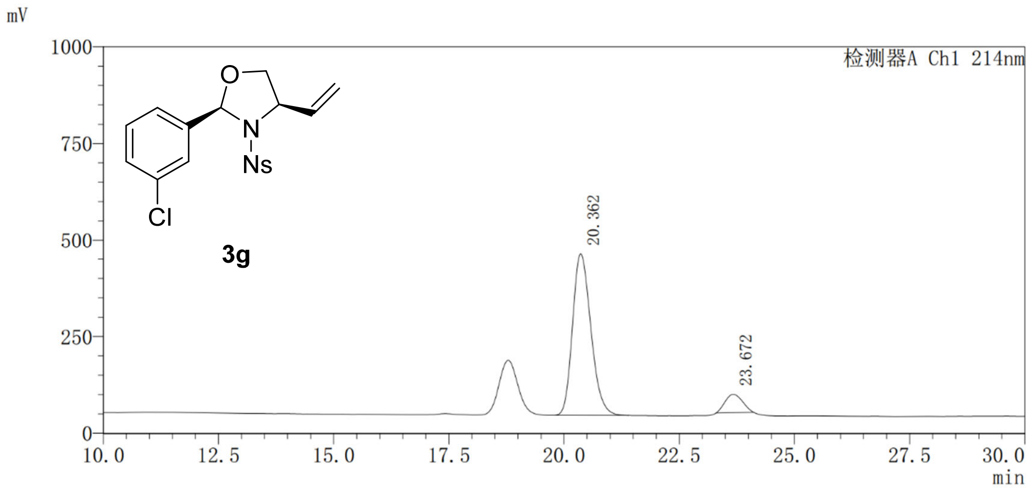

Peak No.	R. Time (min)	Peak Area (mV*min)	Percent (%)
1	20.362	11548122	90.231
2	23.672	1250333	9.769
**Total**		12798455	100.000

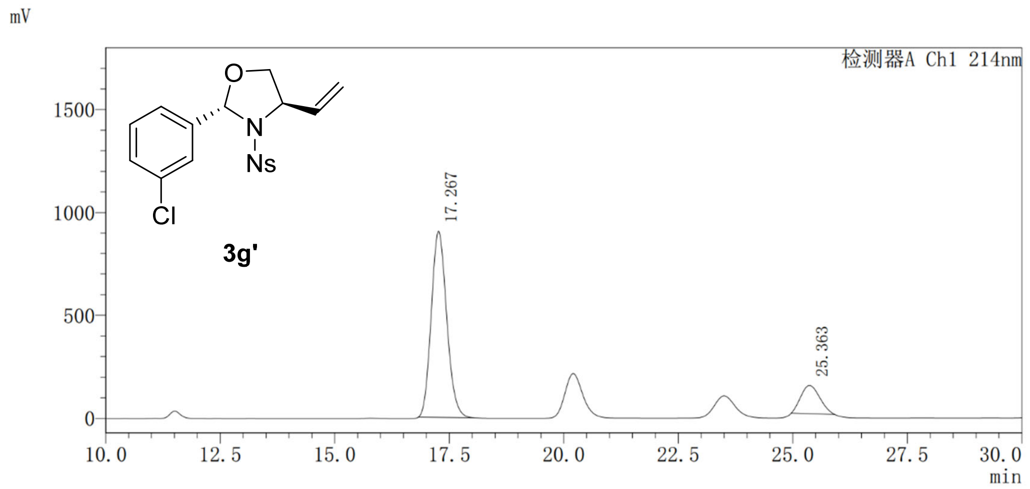

Peak No.	R. Time (min)	Peak Area (mV*min)	Percent (%)
1	17.267	21150588	84.228
2	25.363	3960574	15.772
**Total**		25111163	100.000

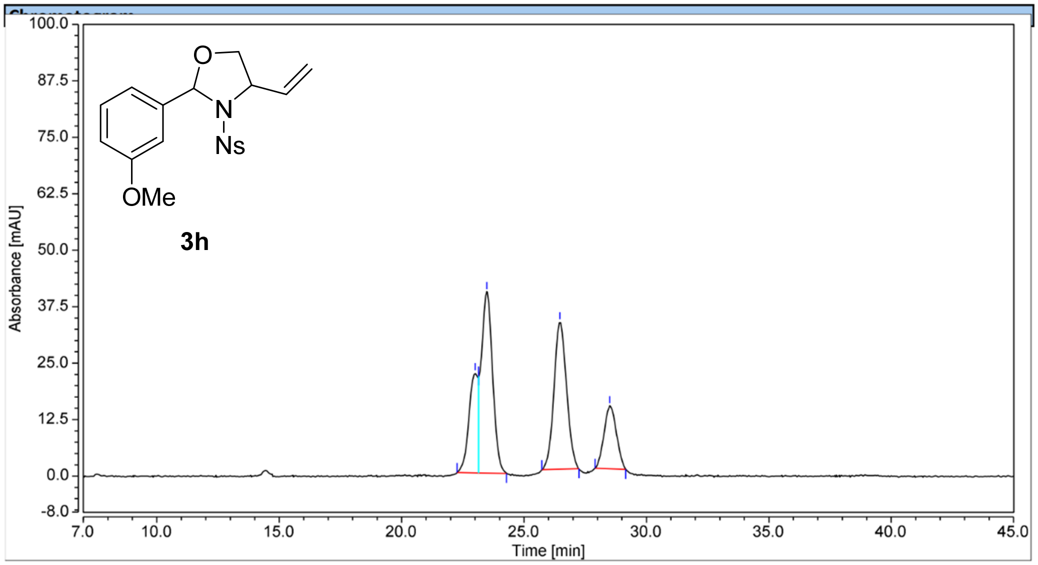

Peak No.	R. Time (min)	Peak Area (mAU*min)	Percent (%)
1	23.012	9.484	15.21
2	23.485	21.466	35.68
3	26.465	19.433	34.21
4	28.505	8.139	14.91
**Total**		58.521	100.000

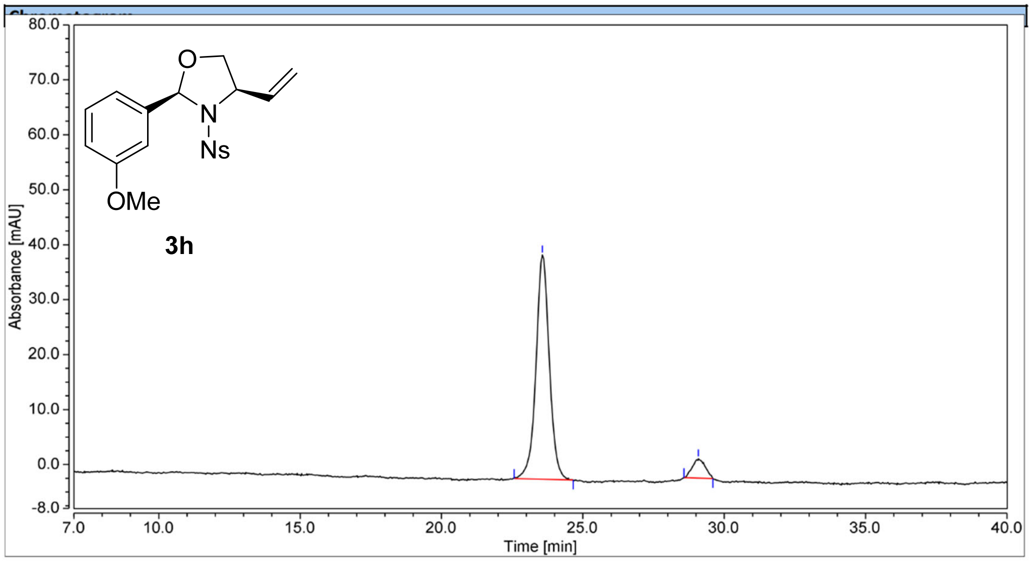

Peak No.	R. Time (min)	Peak Area (mAU*min)	Percent (%)
1	23.566	22.356	92.39
2	29.086	1.841	7.61
**Total**		24.197	100.000

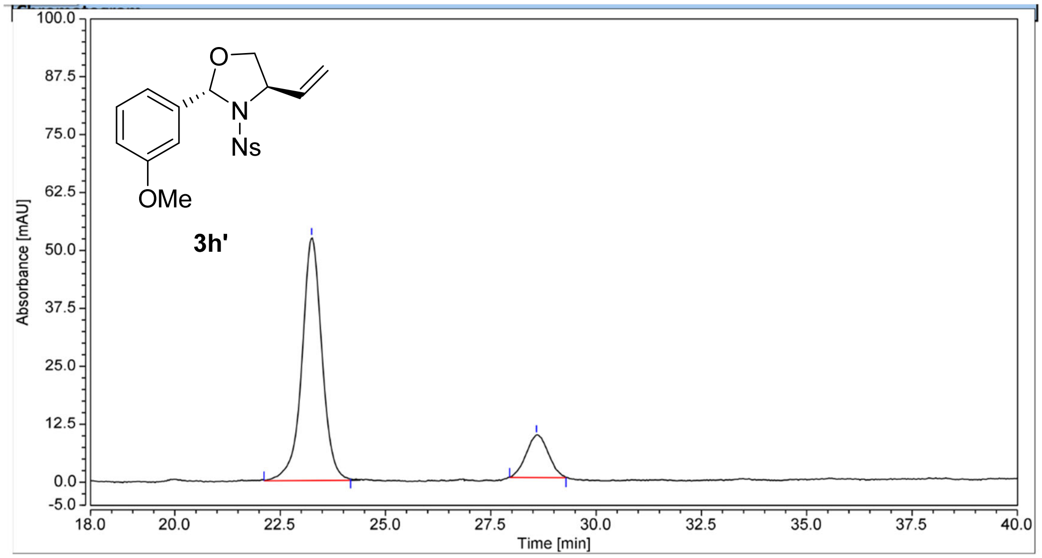

Peak No.	R. Time (min)	Peak Area (mAU*min)	Percent (%)
1	23.246	28.958	83.79
2	28.586	5.604	16.21
**Total**		34.562	100.000

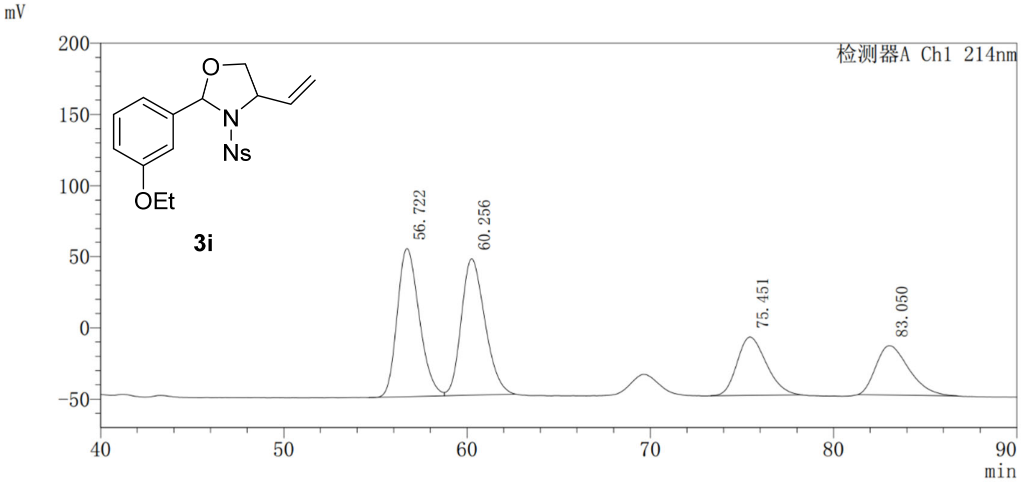

Peak No.	R. Time (min)	Peak Area (mV*min)	Percent (%)
1	56.722	8718047	33.238
2	60.256	8613710	32.840
3	75.451	4553943	17.362
4	83.050	4343843	16.561
**Total**		26229542	100.000

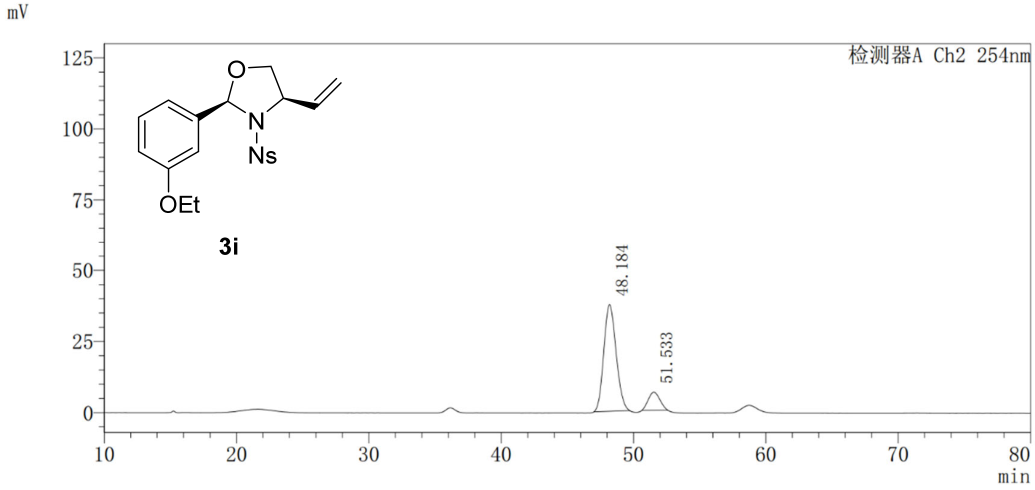

Peak No.	R. Time (min)	Peak Area (mV*min)	Percent (%)
1	48.184	2425553	86.347
2	51.533	383534	13.653
**Total**		2809087	100.000

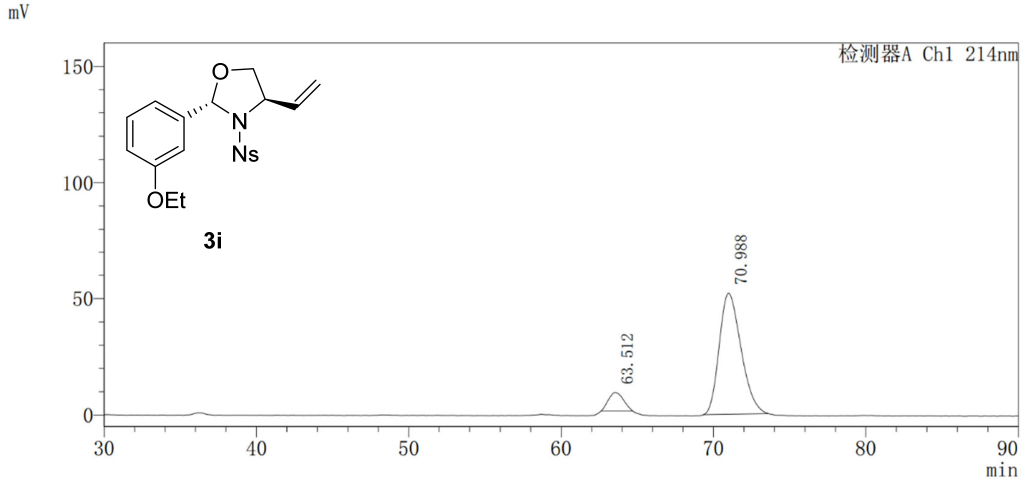

Peak No.	R. Time (min)	Peak Area (mV*min)	Percent (%)
1	63.512	551111	9.653
2	70.988	5158395	90.347
**Total**		5709507	100.000

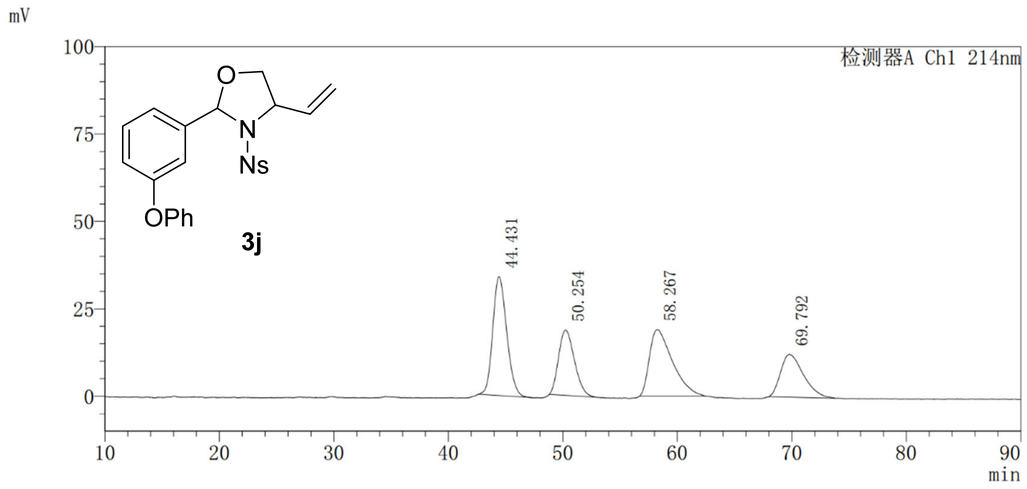

Peak No.	R. Time (min)	Peak Area (mV*min)	Percent (%)
1	44.431	2886227	31.910
2	50.254	1800761	19.909
3	58.267	2655459	29.359
4	69.792	1702331	18.821
**Total**		9044778	100.000

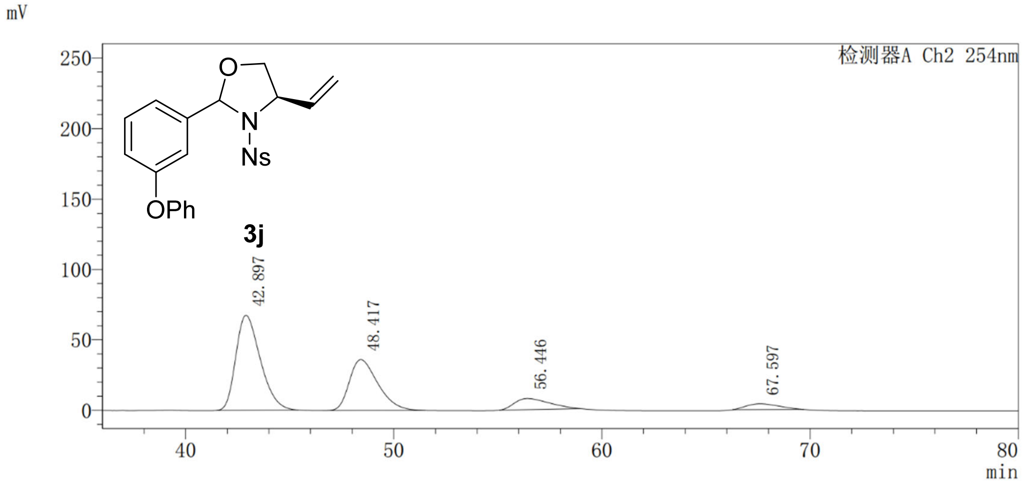

Peak No.	R. Time (min)	Peak Area (mAU*min)	Percent (%)
1	42.897	5519302	53.380
2	48.417	3445528	33.324
3	56.446	921351	8.911
4	67.597	453378	4.385
**Total**		10339559	100.000

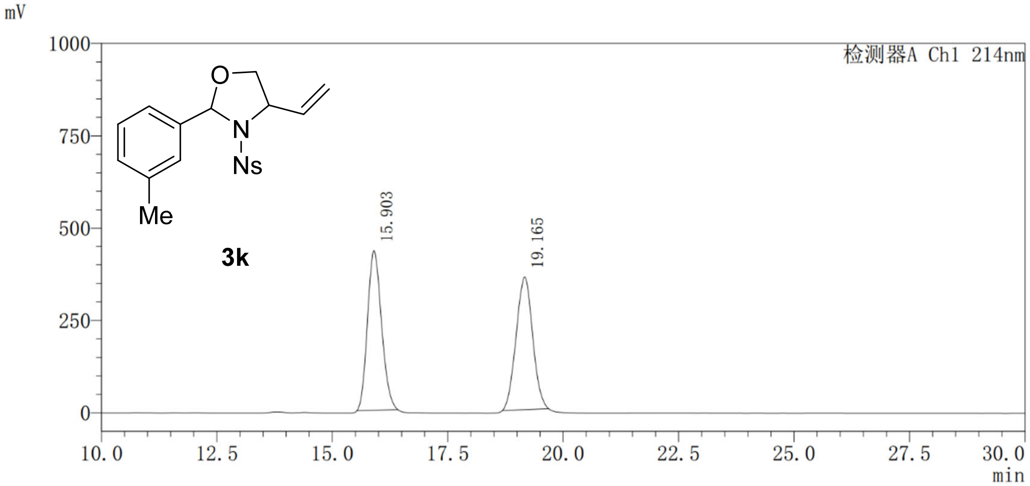

Peak No.	R. Time (min)	Peak Area (mV*min)	Percent (%)
1	15.903	9136961	50.489
2	19.165	8959816	49.511
**Total**		18096777	100.000

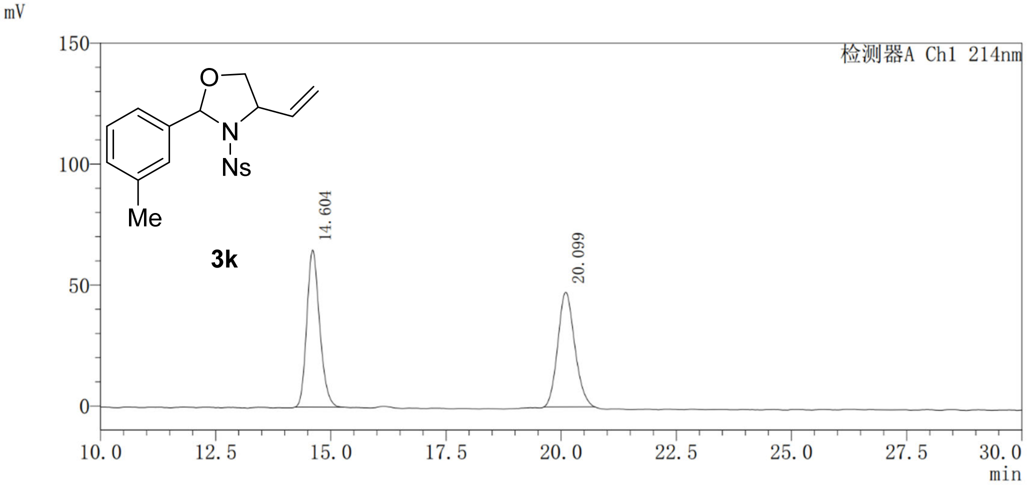

Peak No.	R. Time (min)	Peak Area (mV*min)	Percent (%)
1	14.604	1264614	50.954
2	20.099	1217248	49.046
**Total**		2481861	100.000

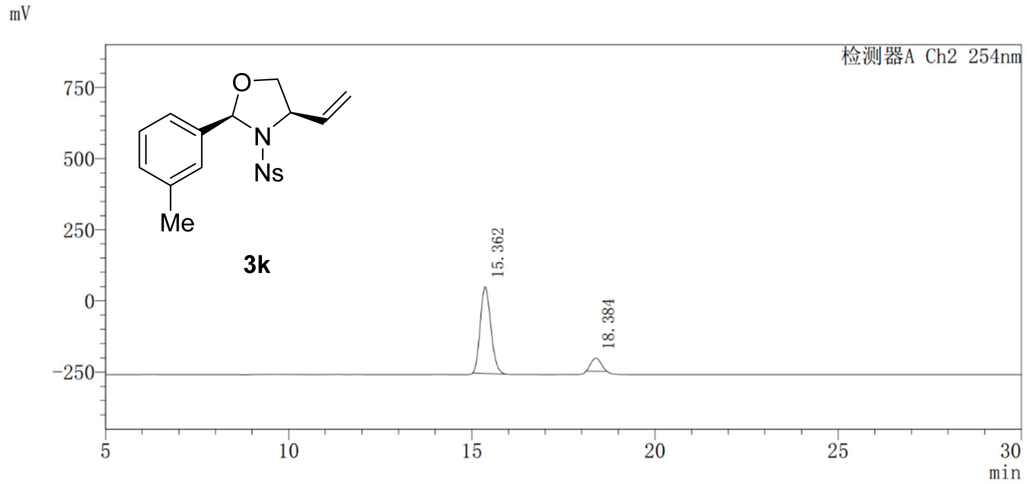

Peak No.	R. Time (min)	Peak Area (mV*min)	Percent (%)
1	15.362	6113905	87.668
2	18.384	860035	12.332
Total		6973940	100.000

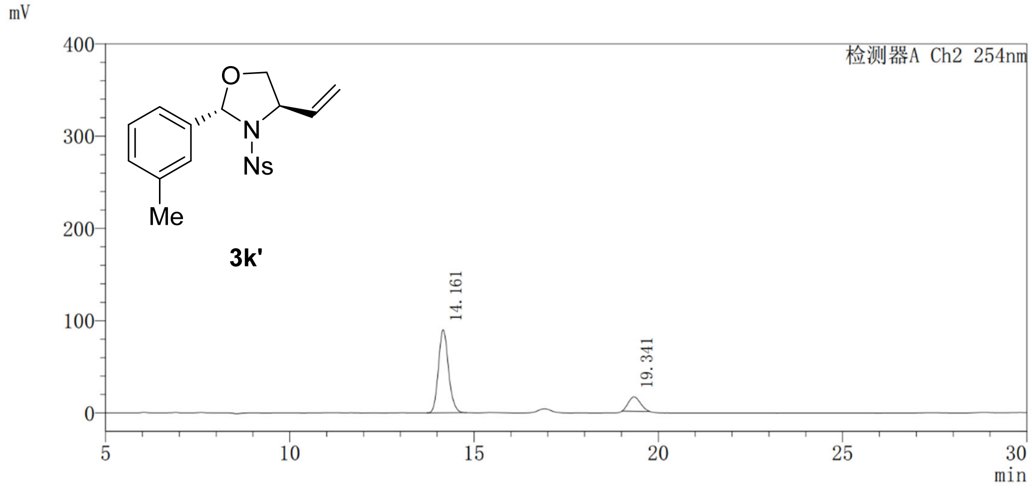

Peak No.	R. Time (min)	Peak Area (mV*min)	Percent (%)
1	14.161	1713244	83.017
2	19.341	350476	16.983
Total		2063720	100.000

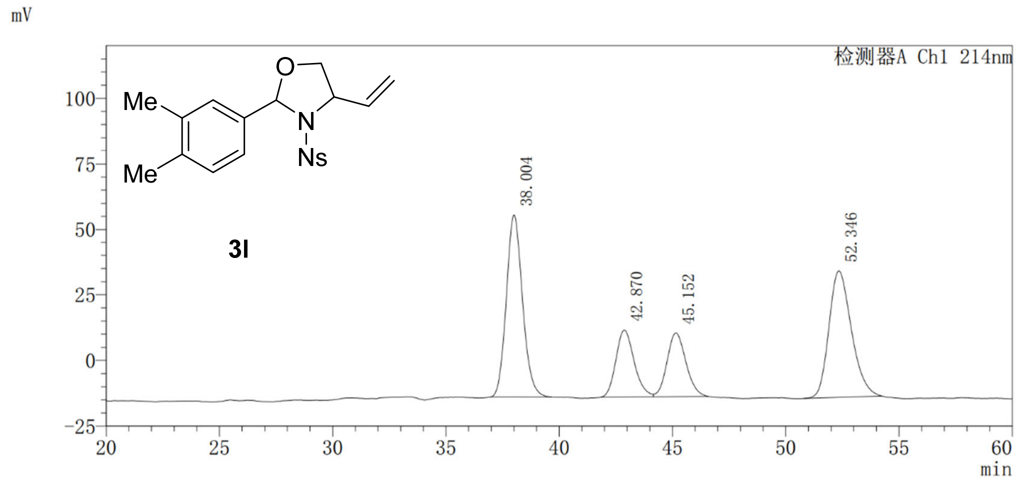

Peak No.	R. Time (min)	Peak Area (mAU*min)	Percent (%)
1	38.004	3449617	35.892
2	42.870	1429065	14.869
3	45.152	1406736	14.636
4	52.346	3325763	34.603
**Total**		9611181	100.000

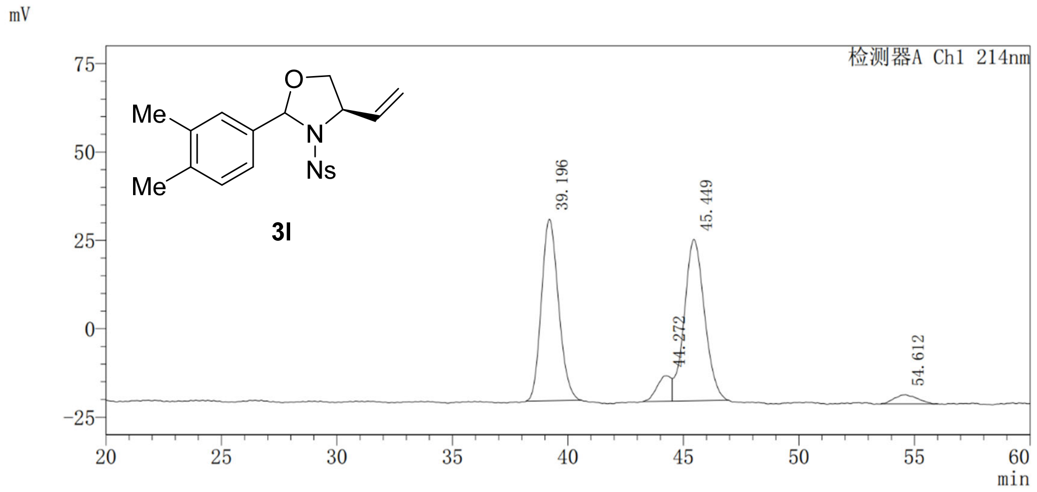

Peak No.	R. Time (min)	Peak Area (mV*min)	Percent (%)
1	39.196	2643867	44.656
2	44.272	306555	5.178
3	45.449	2793066	47.176
4	54.612	177080	2.991
**Total**		5920568	100.000

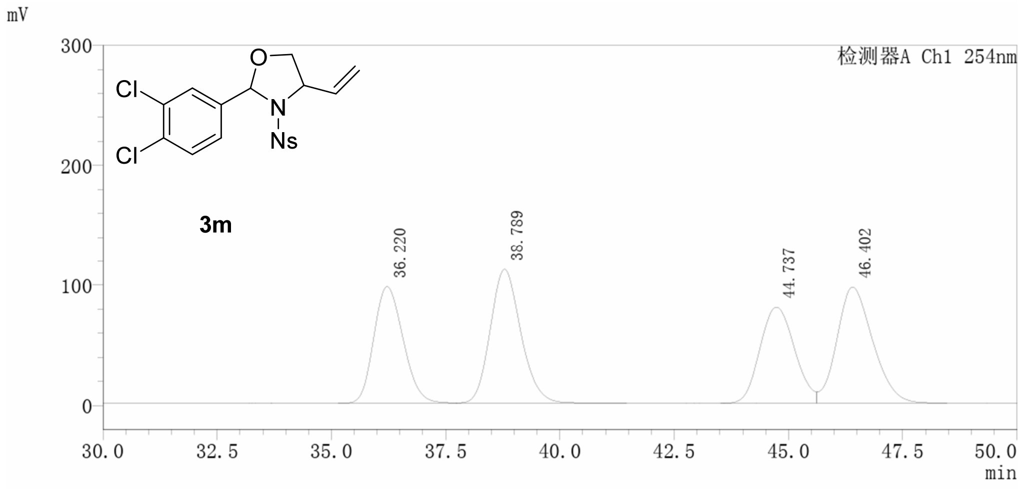

Peak No.	R. Time (min)	Peak Area (mV*min)	Percent (%)
1	36.220	4302473	22.744
2	38.789	5167317	27.316
3	44.737	4199722	22.201
4	46.402	5247190	27.738
**Total**		18916701	100.000

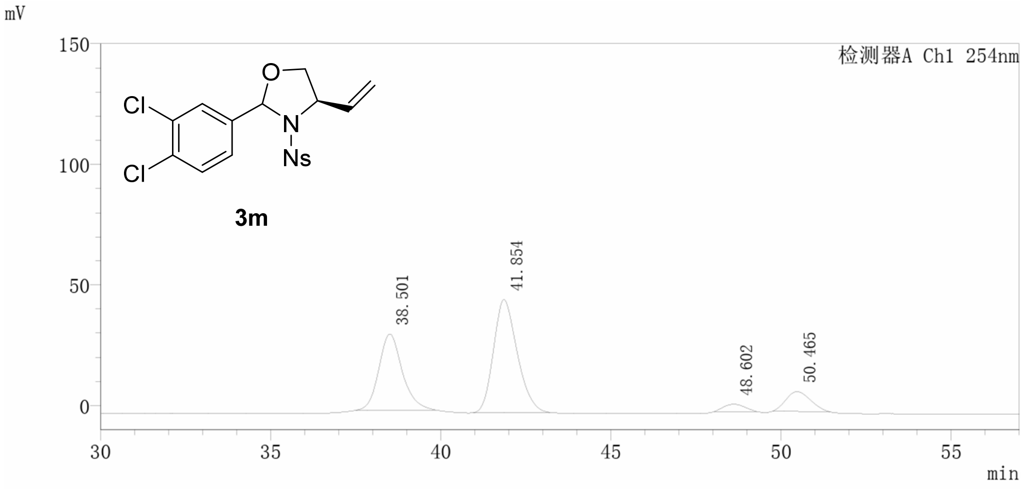

Peak No.	R. Time (min)	Peak Area (mV*min)	Percent (%)
1	38.501	1504615	34.933
2	41.854	2262281	52.523
3	48.602	129457	3.006
4	50.465	410846	9.539
**Total**		4307200	100.000

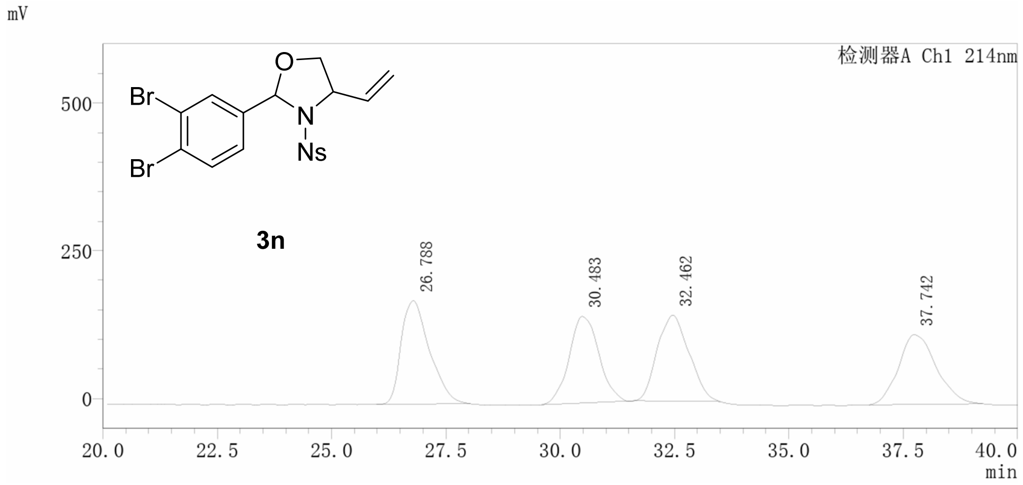

Peak No.	R. Time (min)	Peak Area (mV*min)	Percent (%)
1	26.788	7500028	26.962
2	30.483	6589142	23.688
3	32.462	6990842	25.132
4	37.742	6736615	24.218
**Total**		27816627	100.000

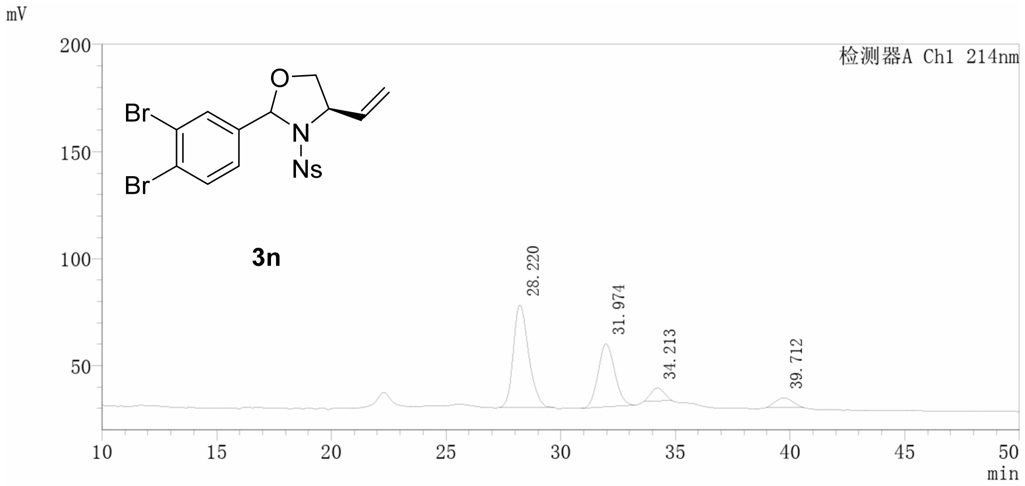

Peak No.	R. Time (min)	Peak Area (mV*min)	Percent (%)
1	28.220	2215831	53.194
2	31.974	1472320	35.345
3	34.213	242395	5.819
4	39.712	234984	5.641
**Total**		4165530	100.000

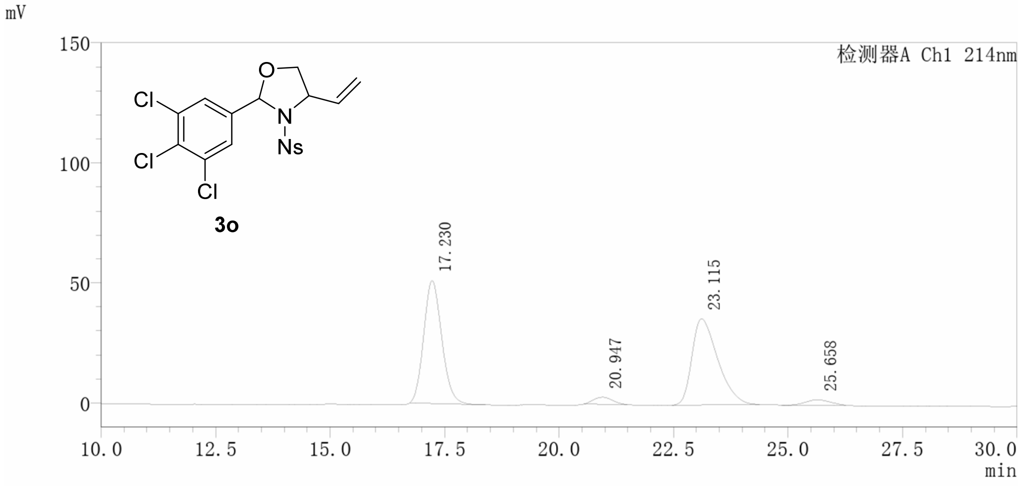

Peak No.	R. Time (min)	Peak Area (mV*min)	Percent (%)
1	17.230	1392884	47.040
2	20.947	85491	2.887
3	23.115	1403079	47.385
4	25.658	79585	2.688
**Total**		2961039	100.000

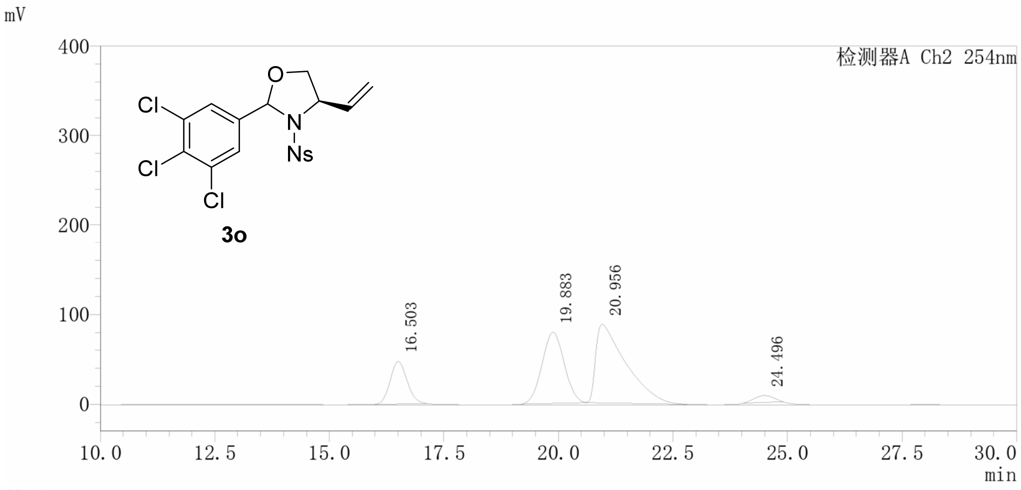

Peak No.	R. Time (min)	Peak Area (mV*min)	Percent (%)
1	16.503	1207558	14.961
2	19.883	2664472	33.011
3	20.956	3976378	49.264
4	24.496	223123	2.764
**Total**		8071531	100.000

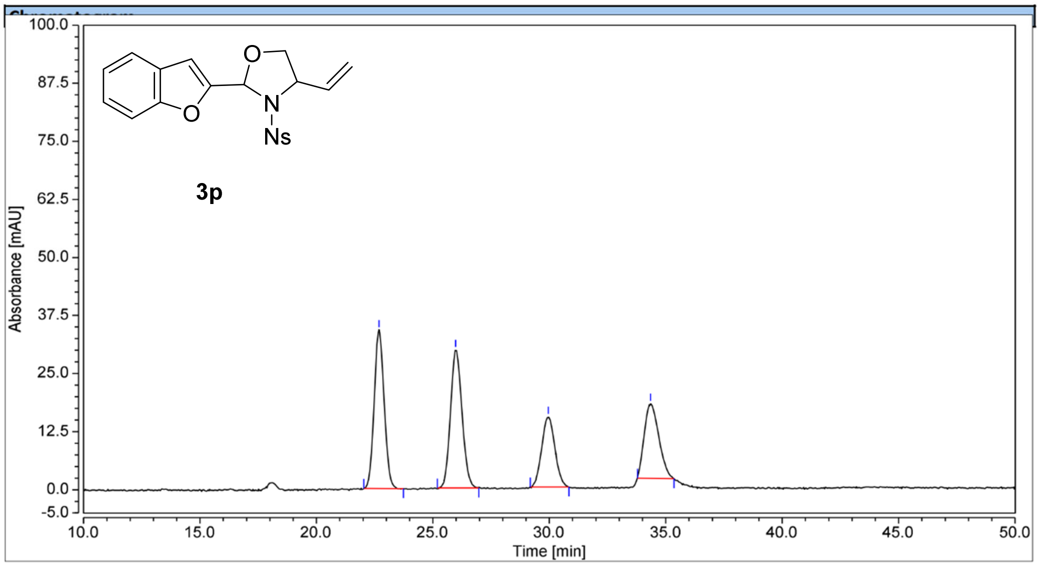

Peak No.	R. Time (min)	Peak Area (mAU*min)	Percent (%)
1	22.685	16.958	30.50
2	25.979	17.106	30.76
3	29.952	9.891	17.79
4	34.345	11.653	20.96
**Total**		55.608	100.000

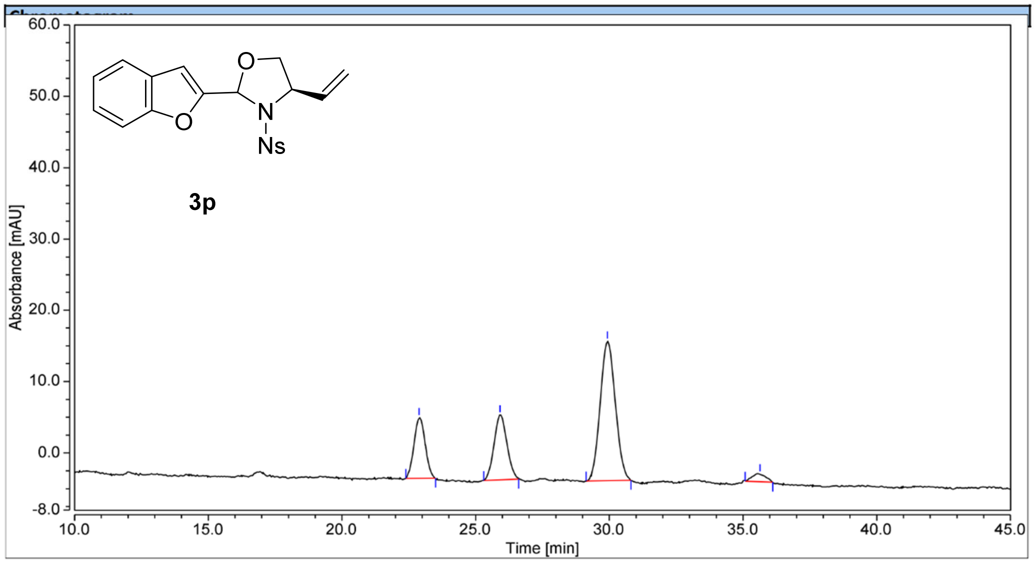

Peak No.	R. Time (min)	Peak Area (mAU*min)	Percent (%)
1	22.886	4.089	18.06
2	25.913	5.111	22.58
3	29.926	12.791	56.51
4	34.639	0.645	2.85

**Total**		22.635	100.000

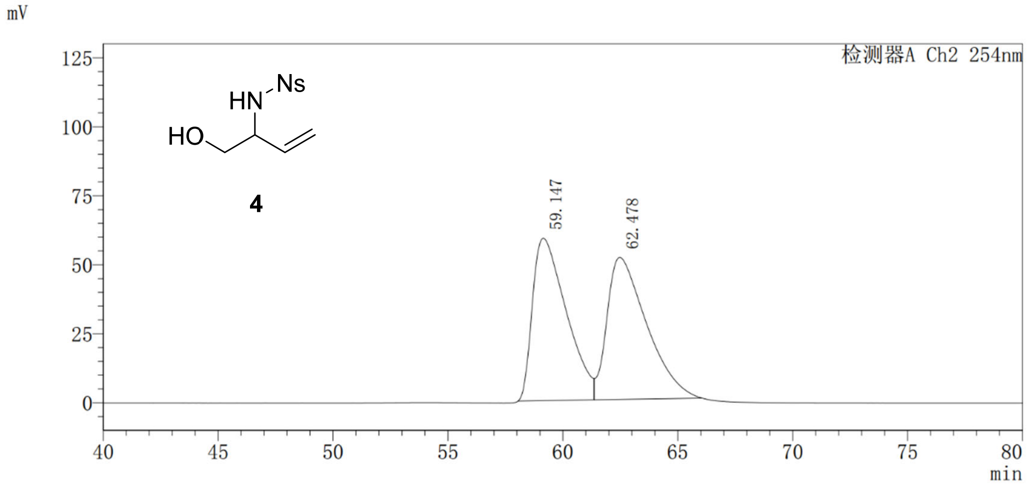

Peak No.	R. Time (min)	Peak Area (mV *min)	Percent (%)
1	59.147	6009579	49.280
2	62.478	6185224	50.720
**Total**		12194803	100.000

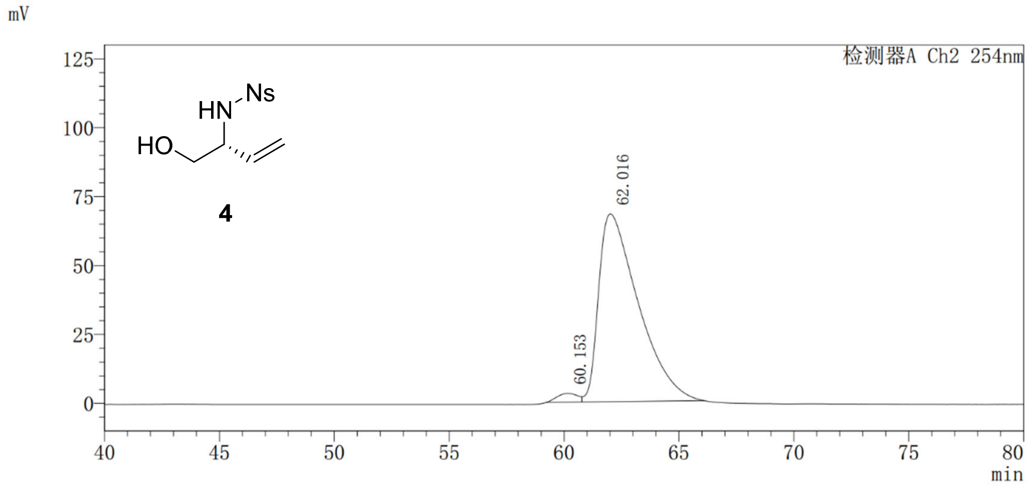

Peak No.	R. Time (min)	Peak Area (mV *min)	Percent (%)
1	60.153	200264	2.386
2	62.016	8193184	97.614
**Total**		8393448	100.000

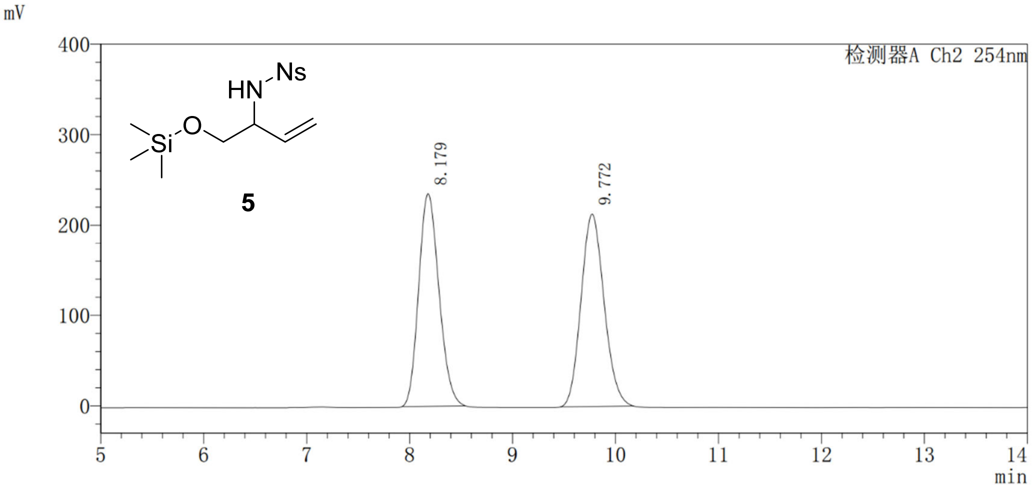

Peak No.	R. Time (min)	Peak Area (mV *min)	Percent (%)
1	8.179	3218288	49.307
2	9.772	3308794	50.693
**Total**		6527082	100.000

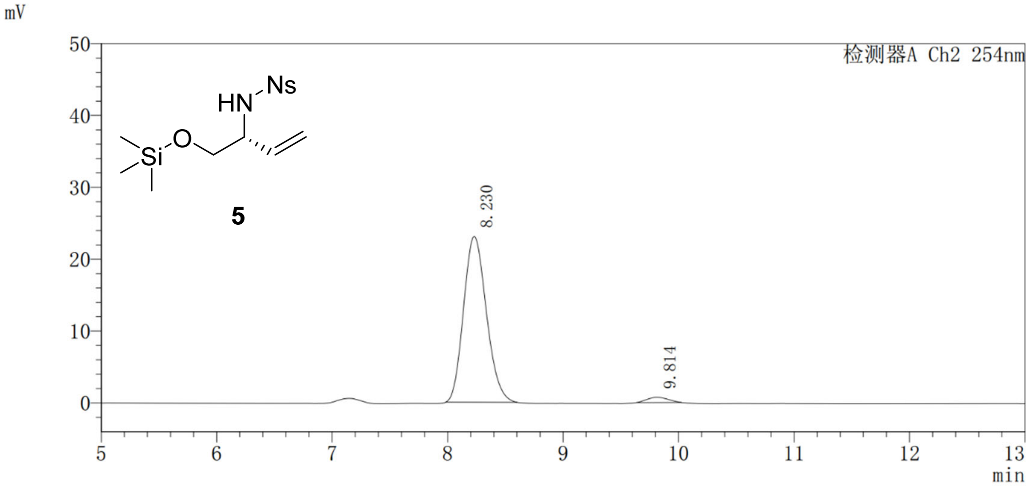

Peak No.	R. Time (min)	Peak Area (mV *min)	Percent (%)
1	8.230	8.230	97.144
2	9.814	9.814	2.856
**Total**		8.230	100.000

9. References

[1] (a) N. Gao, X.-W. Guo, S.-C. Zheng, W.-K. Yang, X.-M. Zhao, *Tetrahedron* **2012**, *68*, 9413. (b) M. Zhang, M. Zhao, P. Zheng, H. Zhang, X. Zhao, *J. Fluorine Chem.* **2016**, *189*, 13.
[2] L. Marin, S. Jerhaoui, E. Kolodziej, R. Guillot, V. Gandon, F. Colobert, E. Schulz, J.

Wencel-Delord, D. Lebœuf, *Adv. Synth. Catal.* **2021**, *363*, 4277.
[3] N. Bodineau, J.-M. Mattali, V. Thimokhin, K. Handoo, J. C. Négrel, M. Chanon, *Org. Lett.* **2000**, *2*, 2303.
[4] F. Tato, V. Reboul, P. Metzner. *J. Org. Chem.* **2008**, *73*, 7837.
